# Isolation and structural characterization of polycyclic diazepine alkaloids from *Pandanus yvanii*

**DOI:** 10.1007/s13659-026-00632-0

**Published:** 2026-07-13

**Authors:** Jin-Ning Chu, Premanand Krishnan, Kien-Thai Yong, Yun-Yee Low, Kuan-Hon Lim

**Affiliations:** 1 School of Pharmacy, University of Nottingham Malaysia, Jalan Broga, 43500 Semenyih, Selangor Malaysia; 2 Department of Chemistry, Faculty of Science, Universiti Malaya, 50603 Kuala Lumpur, Malaysia; 3 Institute of Biological Sciences, Faculty of Science, Universiti Malaya, 50603 Kuala Lumpur, Malaysia

**Keywords:** *Pandanus yvanii*, Pandanaceae, Polycyclic, 1,3-Diazepine, Indolizidine, Pandazepines A–D

## Abstract

Four unprecedented polycyclic alkaloids, designated pandazepines A–D (**1**–**4**), featuring rare diazepine-containing frameworks, were isolated from the leaves of *Pandanus yvanii*. Their structures were elucidated through comprehensive spectroscopic analyses. Pandazepines A and B (**1** and **2**) represent the first examples of tetracyclic pyrrolidinoindoline-1,3-diazepine alkaloids, with their absolute configurations established by comparison of experimental and TDDFT-calculated ECD spectra. Pandazepines C and D (**3** and **4**) possess unprecedented pentacyclic fused-ring systems comprising linearly fused 1,3-diazepine, γ-lactam, and indolizidine units. The relative configurations of **3** and **4** were determined through GIAO NMR chemical shift calculations in combination with DP4+ probability analysis. A putative biosynthetic pathway for **1**–**4**, originating from putrescine is proposed. Collectively, these findings expand the structural diversity of diazepine-containing natural products and provide new insights into the biosynthetic capabilities of the *Pandanus* genus.

**Graphical Abstract:**

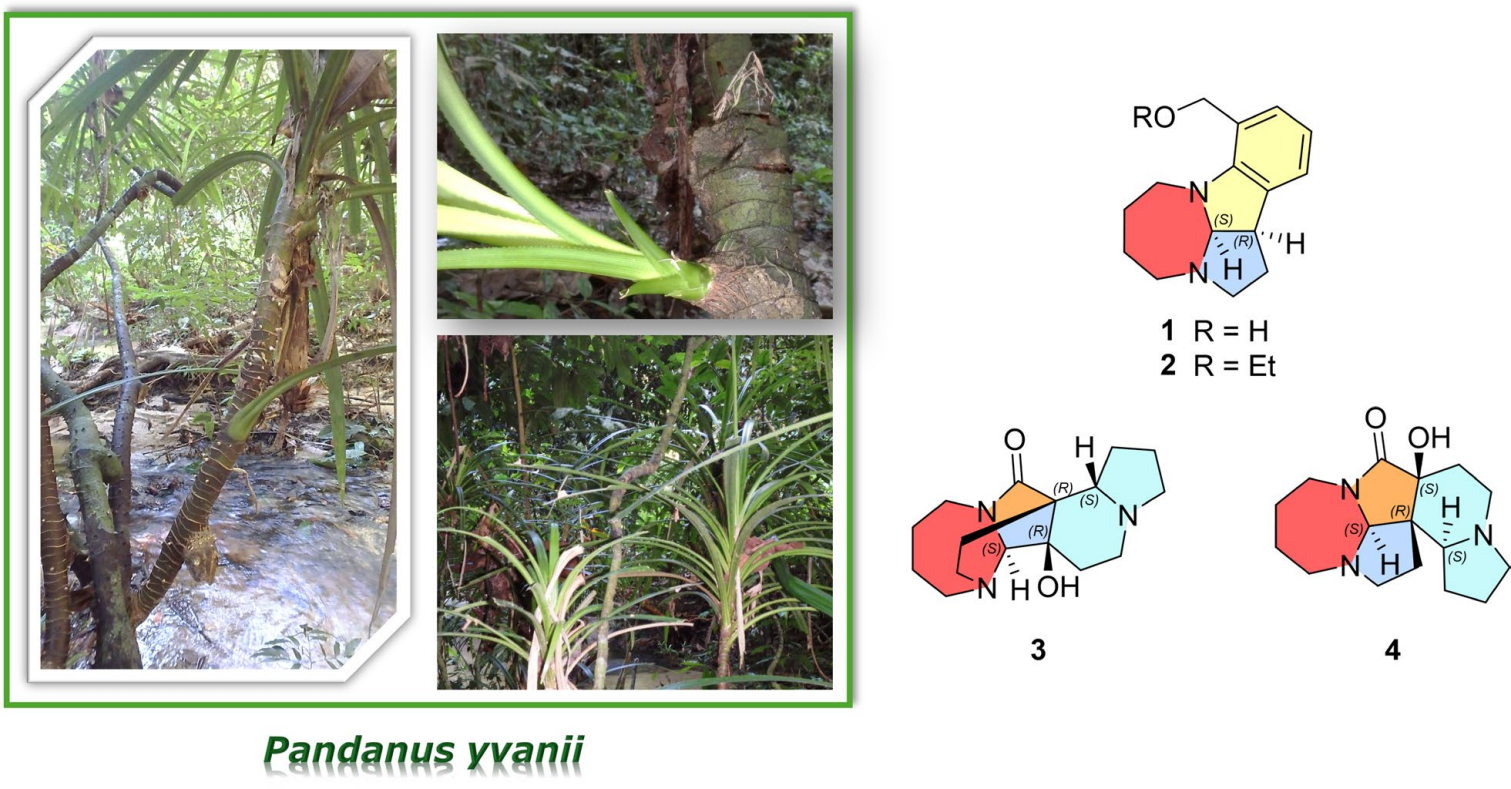

**Supplementary Information:**

The online version contains supplementary material available at 10.1007/s13659-026-00632-0.

## Introduction

**Fig. 1:**
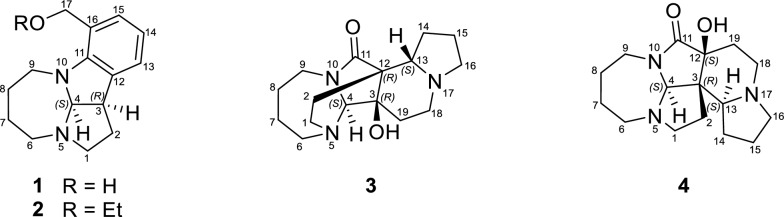
Chemical structures of **1**–**4**

The Pandanaceae is a pantropical plant family from which several *Pandanus* species have yielded structurally unique alkaloids with diverse biological activities. *Pandanus yvanii* Solms, locally known as *Mengkuang Tikus* in Malay, is distributed across the Malay Peninsula, southern Thailand, Sumatra, Bangka Island, and Borneo, typically inhabiting riverbanks, freshwater swamps, and peat swamp forests. This slender, clustering species forms dense thickets supported by short prop roots [1]. Despite its distinctive morphology and broad distribution, *P. yvanii* remains phytochemically unexplored, with existing literature limited to taxonomic descriptions and rediscovery reports. In contrast, the phytochemistry of other *Pandanus* species has revealed a chemically distinctive alkaloid profile, prompting increasing scientific interest in recent years. These alkaloids are predominantly characterized by 9- or 18-carbon skeletons bearing a terminal γ-alkylidene α,β-unsaturated γ-lactone or γ-lactam moiety, with some further incorporating pyrrolidine, piperidine, or indolizidine rings. The structural similarities among these alkaloids suggest a common biogenetic origin, with glutamic acid and leucine proposed as biosynthetic precursors [2–10]. Herein, we report the first phytochemical investigation of *P. yvanii*, leading to the isolation of four novel 1,3-diazepine-containing alkaloids, designated as pandazepines A–D (**1**–**4**) (Fig. 1).

## Results and discussion

Pandazepine A (**1**) was isolated in trace amounts as a brown amorphous, with $[\alpha]^{25}_{\mathrm{D}}-12$ (*c* 0.16, CHCl_3_). Its UV spectrum exhibited absorption maxima at 212, 264, and 312 nm, characteristic of an indoline chromophore. The IR spectrum displayed an absorption band attributable to a hydroxyl group at 3362 cm^-1^. HRDARTMS analysis revealed an [M + H]^+^ peak at *m/z* 245.1663, consistent with the molecular formula C_15_H_20_N_2_O. The ^13^C NMR spectrum (Table 1) accounted for 15 carbon signals, comprising six aromatic carbons (including two quaternary and one nitrogen-substituted), seven methylene carbons (including three aminomethylenes and one oxymethylene), and two aliphatic methine carbons. Notably, one aliphatic methine carbon exhibited a distinctly downfield resonance at *δ*_C_ 87.7 (C-4), suggesting a diazomethine functionality. Analysis of the ^1^H NMR spectrum (Table 1), supported by HSQC correlations, identified 19 proton signals, including three aromatic protons at *δ*_H_ 6.85 (H-13), *δ*_H_ 6.51 (H-14), and *δ*_H_ 6.88 (H-15), consistent with a trisubstituted aromatic ring. The significantly deshielded diazomethine proton appeared at *δ*_H_ 4.62 (H-4). Of the seven methylene groups, three showed diastereotopic proton pairs at *δ*_H_ 2.41/2.59 (CH_2_-1), *δ*_H_ 2.70/2.71 (CH_2_-6), and *δ*_H_ 3.29/3.86 (CH_2_-9) were assigned to aminomethylene groups, while an AB doublet pair at *δ*_H_ 4.45/4.51 corresponded to the oxymethylene group (CH_2_-17). The molecular formula required one additional proton not observed in the ^1^H NMR spectrum, which was assigned to a hydroxyl group, consistent with the IR data.

**Table 1:** ^1^H and ^13^C NMR data of **1** (CD_3_OD) and **2** (CDCl_3_) (*δ* in ppm)^a^

**No**	**1**^b^		**2**^b^	
	*δ*_C_, type	*δ*_H_, mult. (*J* in Hz)	*δ*_C_, type	*δ*_H_, mult. (*J* in Hz)
1	51.9, CH_2_	2.41, ddd (9.3, 7.7, 6.3) (β)	52.3, CH_2_	2.51, m (β)
		2.59, ddd (9.3, 6.6, 5.2) (α)		3.04, m (α)
2	31.9, CH_2_	1.74, m (β)	32.2, CH_2_	1.99, m (β)
		2.26, m (α)		2.50, m (α)
3	44.8, CH	3.86, m (α)	45.3, CH	4.06, m (α)
4	87.7, CH	4.62, d (8.5) (pseudo-ax, α)	88.9, CH	5.05, m (pseudo-ax, α)
6	52.7, CH_2_	2.70, m (pseudo-ax, β)	52.5, CH_2_	2.76, ddd (12.2, 8.1, 2.2) (pseudo-ax, β)
		2.71, m (pseudo-eq, α)		3.18, m (pseudo-eq, α)
7	26.8, CH_2_	1.77, m (pseudo-eq, β)	26.1, CH_2_	1.97, m (pseudo-eq, β)
		1.80, m (pseudo-ax, α)		1.98, m (pseudo-ax, α)
8	29.3, CH_2_	1.81, m (pseudo-ax, β)	30.7, CH_2_	1.87, tq (10.5, 6.1) (pseudo-ax, β)
		1.82, m (pseudo-eq, α)		2.01, m (pseudo-eq, α)
9	49.9, CH_2_	3.29, ddd (12.6, 7.4, 4.7) (pseudo-ax, α)	50.3, CH_2_	3.26, ddd (13.5, 10.6, 2.1) (pseudo-ax, α)
		3.86, m (pseudo-eq, β)		4.10, m (pseudo-eq, β)
11	148.9, C	–	149.5, C	–
12	132.7, C	–	124.2, C	–
13	123.4, CH	6.85, d (7.4)	124.2, CH	6.99, d (7.3)
14	117.4, CH	6.51, t (7.4)	118.3, CH	6.67, t (7.3)
15	130.3, CH	6.88, d (7.4)	131.8, CH	7.00, d (7.3)
16	120.1, C	–	117.6, C	–
17	61.5, CH_2_	4.45, d (11.9)	70.7, CH_2_	4.39, d (11.2)
		4.51, d (11.9)		4.49, d (11.2)
18	–	–	65.2, CH_2_	3.51, dq (8.7, 7.0)
		–		3.55, dq (8.7, 7.0)
19	–	–	15.3, CH_3_	1.24, t (7.0)

^a^Measured at 600 and 150 MHz

^b^Assignments based on the ^1^H-^1^H COSY, HSQC, and HMBC spectra

**Fig. 2:**
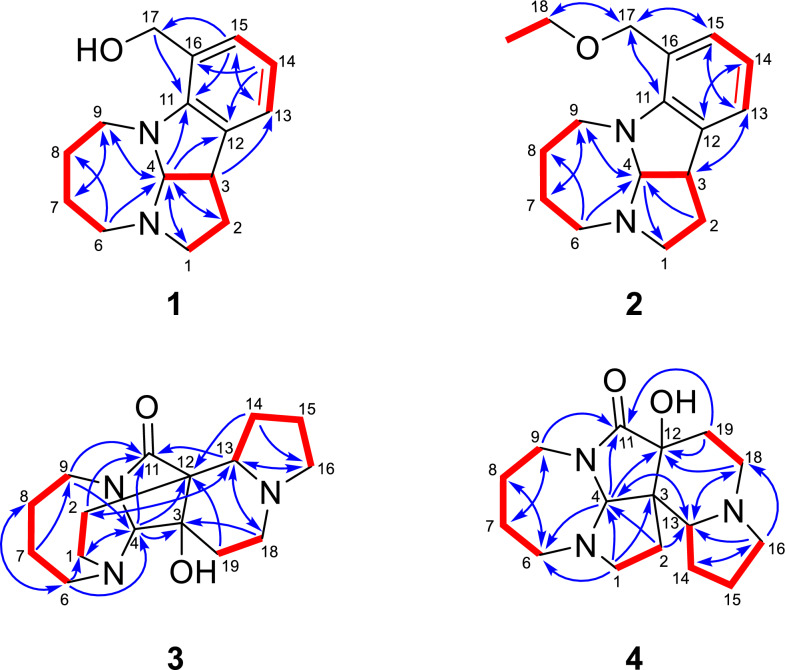
^1^H-.^1^H COSY (red, bold) and selected HMBC (blue, arrows) correlations of **1**–**4**

The planar structure of **1** was elucidated by combining ^1^H-^1^H COSY and HMBC correlations (Fig. 2). The ^1^H-^1^H COSY spectrum revealed three spin systems. The first comprised three contiguous aromatic protons corresponding to the CH-13–CH-14–CH-15 fragment, corroborated by characteristic aromatic coupling constants for H-13 (d, *J* = 7.4 Hz), H-14 (t, *J* = 7.4 Hz), and H-15 (d, *J* = 7.4 Hz). Three-bond HMBC correlations from H-14 to C-12 and C-16, and from H-15 to C-11 and C-13, enabled the assignment of the aromatic carbons at *δ*_C_ 148.9 (C-11), 132.7 (C-12), 123.4 (C-13), 117.4 (C-14), 130.3 (C-15), and 120.1 (C-16). The placement of a hydroxymethyl substituent at C-16 was supported by HMBC correlations from the oxymethylene protons (CH_2_-17) to C-11, and from H-15 to C-17.

The second spin system, CH_2_-1–CH_2_-2–CH-3–CH-4, identified in the ^1^H-^1^H COSY spectrum, was connected to the aromatic ring based on key HMBC correlations from H-3 to C-13 and from H-4 to C-11 and C-12. These data indicate that C-3 is directly bonded to the aromatic carbon C-12, while C-4 is linked to C-11 via a nitrogen atom, thereby forming a pyrrolidine ring and establishing an indoline scaffold consistent with the UV absorption profile. Further HMBC correlations from H-4 to C-1 and C-2 support the fusion of an additional pyrrolidine ring to the indoline unit, establishing a pyrrolidinoindoline core. This assignment was corroborated by the strongly deshielded chemical shifts of the diazomethine group at C-4 (*δ*_C_ 87.7, *δ*_H_ 4.62), which are characteristic of pyrrolidinoindoline alkaloids [11, 12]. The third spin system, CH₂-6–CH₂-7–CH₂-8–CH₂-9, was connected to both nitrogen atoms of the pyrrolidinoindoline core through three-bond HMBC correlations from H_2_-6 and H_2_-9 to C-4, thereby establishing a seven-membered 1,3-diazepine ring. Taken together, the comprehensive 2D NMR data established the structure of **1** as a novel tetracyclic pyrrolidinoindoline-1,3-diazepine alkaloid (Fig. 2).

**Fig. 3:**
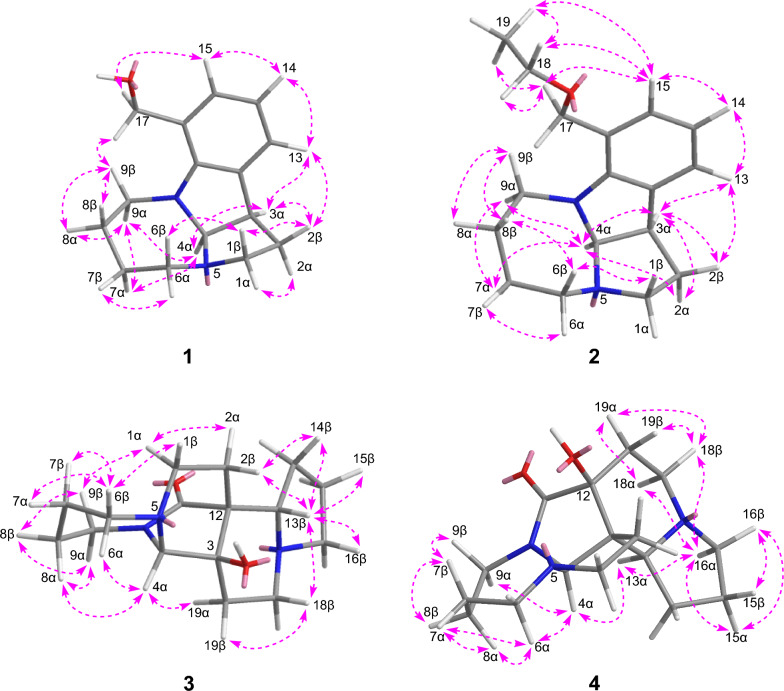
Selected NOESY correlations of **1**–**4**

The relative configuration at the two chiral centres, C-3 and C-4 in **1**, was subsequently determined through coupling constant analysis and NOESY data (Fig. 3). A key NOESY correlation between H-3 (*δ*_H_ 3.86) and H-4 (*δ*_H_ 4.62), along with the large coupling constant observed for H-4 (d, *J* = 8.5 Hz), indicated a *cis* (α-oriented) relationship between these protons at the C-3/C-4 ring junction of the pyrrolidinoindoline system. Further support for this stereochemical assignment was provided by additional NOESY correlations: H-3α/H-2α, H-3α/H-13, H-13/H-2β, H-2α/H-1α, and H-2β/H-1β.

**Fig. 4:**
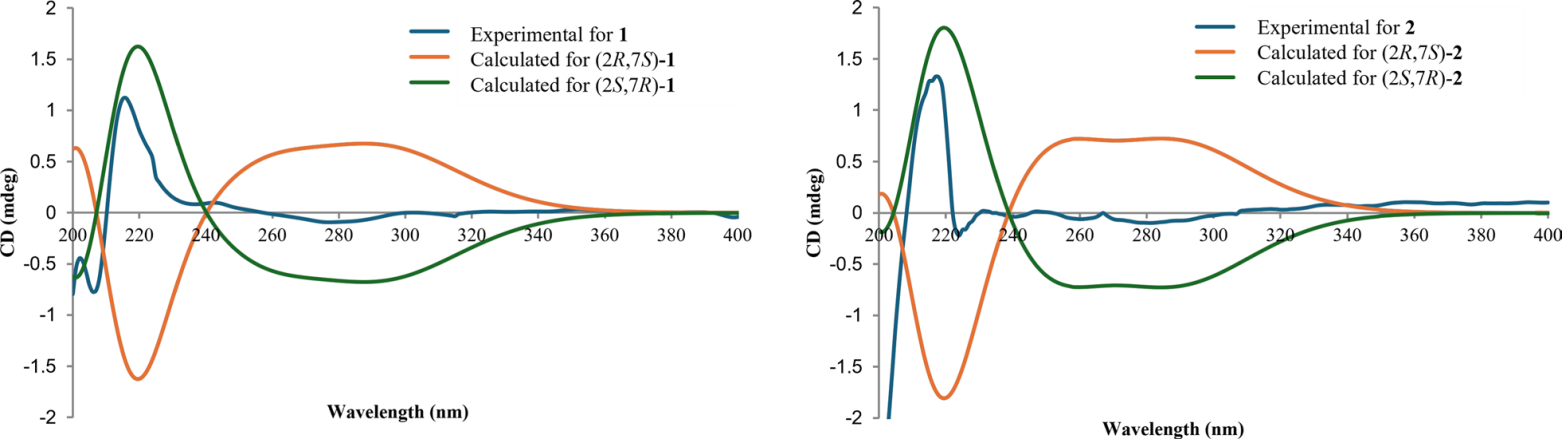
Experimental and calculated TDDFT-ECD spectra of **1** and **2** in CH_3_CN

Within the 1,3-diazepine ring, NOESY correlations among H-4 (*δ*_H_ 4.62), H-7 (*δ*_H_ 1.80), and H-9 (*δ*_H_ 3.29) indicated their pseudo-axial (α) orientation, consistent with a twist-chair conformation, in which the *N*-5 lone pair is oriented *cis* to H-4. This assignment was further supported by additional NOESY correlations, including H-6α/H-7β, H-6β/H-1β, H-8β/H-9β, H-8α/H-9α, and H-8α/H-9β. Collectively, these data established the relative configuration of **1** as 3R*,4S*. Moreover, the experimental ECD spectrum of **1** showed good agreement with the calculated TDDFT-ECD spectrum for the 3*R*,4*S* enantiomer, thereby confirming its absolute configuration (Fig. 4).

Pandazepine B (**2**) was isolated in trace amounts as a brown amorphous, with $[\alpha]^{25}_{\mathrm{D}}-5$ (*c* 0.15, CHCl_3_). Similar to **1**, its UV absorption maxima at 210, 264, and 313 nm indicated the presence of an indoline chromophore. However, unlike **1**, the IR spectrum of **2** did not exhibit a hydroxyl absorption band. The molecular formula was established as C_17_H_24_N_2_O by HRDARTMS, which revealed an [M + H]^+^ peak at *m/z* 273.1960, consistent with the addition of an ethyl unit relative to **1**. Comparison of the ^1^H and ^13^C NMR data of **2** with those of **1** (Table 1) revealed close structural similarity, with **2** displaying additional resonances corresponding to an ethoxy group (OCH_2_-18, *δ*_H_ 3.51 and 3.55; CH_3_-19, *δ*_H_ 1.24). Comprehensive analysis of ^1^H-^1^H COSY and HMBC correlations (Fig. 2) confirmed that **2** possesses the same pyrrolidinoindoline-1,3-diazepine tetracyclic core as **1**. The key structural difference resides at C-17, where the hydroxyl group in **1** is replaced by an ethoxy substituent in **2**, supported by HMBC correlations from H_2_-17 to C-18. The relative configuration at C-3 and C-4 in **2** was determined through analysis of NOESY data (Fig. 3). The close similarity in chemical shifts and NOESY correlations between **2** and **1** indicated that both compounds share the same stereochemistry at these positions. The experimental ECD spectrum of **2** closely resembled that of **1** and matched the calculated TDDFT-ECD spectrum for 3*R*,4*S* (Fig. 4), thereby establishing the absolute configuration*.* Collectively, these data established **2** as the *O*-ethyl derivative of **1** (Fig. 2).

Pandazepine C (**3**) was isolated as a UV-inactive brown amorphous, with $[\alpha]^{25}_{\mathrm{D}}-9$ (*c* 0.14, CHCl_3_). Its IR spectrum displayed absorption bands at 1698 and 3385 cm⁻^1^, attributable to a lactam carbonyl and a hydroxyl group, respectively. HRDARTMS revealed an [M + H]^+^ peak at *m/z* 292.2039, consistent with the molecular formula C_16_H_25_N_3_O_2_. The ^13^C NMR spectrum (Table 2) showed 16 carbon resonances comprising 11 methylenes (including five aminomethylenes), two aliphatic methines, and three non-protonated carbons: a carbonyl carbon at *δ*_C_ 175.9 (C-11), an oxygenated tertiary carbon at *δ*_C_ 70.5 (C-3), and a quaternary carbon at *δ*_C_ 48.8 (C-12). The ^1^H NMR and HSQC spectra (Table 2) identified 25 proton signals arising from 11 methylene groups (including five aminomethylenes), an aminomethine at *δ*_H_ 1.97 (H-13), a notably deshielded diazomethine at *δ*_H_ 4.03 (H-4), and a hydroxyl proton at *δ*_H_ 3.78.

**Table 2:** ^1^H and ^13^C NMR data of **3** and **4** (CDCl_3_, *δ* in ppm)^a^

**No**	**3**^b^		**4**^b^	
	*δ*_C_, type	*δ*_H_, mult. (*J* in Hz)	*δ*_C_, type	*δ*_H_, mult. (*J* in Hz)
1	41.5, CH_2_	2.49, m (eq, β)	54.7, CH_2_	2.43, q (9.3) (ax)
		2.53, td (12.3, 5.5) (ax, α)		3.24, t (9.3) (eq)
2	24.6, CH_2_	1.58, dd (13.2, 5.3) (eq, α)	19.8, CH_2_	1.79, m (ax)
		1.85, m (ax, β)		2.26, m (eq)
3	70.5, C	–	54.4, C	–
4	76.4, CH	4.03, s (pseudo-ax, α)	82.2, CH	3.57, s (pseudo-ax, α)
6	54.8, CH_2_	2.64, ddd (15.0, 12.6, 2.9) (pseudo-ax, α)	55.1, CH_2_	2.00, m (pseudo-ax, α)
		2.89, dt (14.8, 2.9) (pseudo-eq, β)		3.10, ddd (11.9, 4.6, 2.7) (pseudo-eq, β)
7	24.9, CH_2_	1.65, m (pseudo-eq, α)	27.3, CH_2_	1.56, m (pseudo-ax, β)
		1.85, m (pseudo-ax, β)		1.91, m (pseudo-eq, α)
8	26.3, CH_2_	1.67, m (pseudo-ax, α)	26.4, CH_2_	1.67, m (pseudo-ax, α)
		2.01, m (pseudo-eq, β)		2.10, m (pseudo-eq, β)
9	42.7, CH_2_	3.24, ddd (13.6, 3.2, 2.2) (pseudo-ax, α)	42.6, CH_2_	3.27, dd (13.6, 4.9) (pseudo-ax, α)
		3.57, td (13.6, 4.1) (pseudo-eq, β)		3.66, td (13.6, 5.1) (pseudo-eq, β)
11	175.9, C	–	175.9, C	–
12	48.8, C	–	77.1, C	–
13	63.8, CH	1.97, m (ax, β)	65.7, CH	1.76, m (ax, α)
14	22.6, CH_2_	1.64, m (β)	25.4, CH_2_	1.72, m (β)
		2.76, m (α)		1.84, m (α)
15	21.0, CH_2_	1.65, m (β)	22.0, CH_2_	1.74, m (α)
		1.83, m (α)		1.84, m (β)
16	54.7, CH_2_	2.08, q (8.9) (β)	54.9, CH_2_	1.98, m (α)
		3.10, td (8.5, 1.5) (α)		3.04, m (β)
18	49.2, CH_2_	2.26, ddd (13.6, 11.0, 2.9) (ax, β)	50.1, CH_2_	1.87, m (ax, α)
		2.99, ddd (11.0, 5.1, 2.2) (eq, α)		3.01, ddd (11.4, 5.7, 2.1) (eq, β)
19	32.3, CH_2_	1.72, m (eq, β)	27.6, CH_2_	1.70, m (ax, β)
		2.01, m (ax, α)		2.24, dt (13.6, 2.5) (eq, α)
OH	–	3.78, br s	–	4.51, br s

^a^Measured at 600 and 150 MHz

^b^Assignments based on the ^1^H-^1^H COSY, HSQC, and HMBC spectra

^1^H-^1^H COSY analysis (Fig. 2) revealed four spin systems: CH_2_-6–CH_2_-7–CH_2_-8–CH_2_-9, CH-13–CH_2_-14–CH_2_-15–CH_2_-16, and two CH_2_CH_2_ fragments (C-1–C-2 and C-18–C-19). The downfield aminomethylene signals for CH_2_-6 (*δ*_H_ 2.64 and 2.89) and CH_2_-9 (*δ*_H_ 3.24 and 3.57), coupled with three-bond HMBC correlations from these protons to the diazomethine carbon (C-4), established a 1,3-diazepine ring analogous to those in **1** and **2**. Additional HMBC correlations from H_2_-9 to C-11, and from H-4 to C-3, C-11, and C-12 indicated fusion of a γ-lactam ring to the 1,3-diazepine ring at *N*-10 and C-4. The chemical shifts and coupling constants of the CH-13–CH_2_-14–CH_2_-15–CH_2_-16 and CH_2_-18–CH_2_-19 fragments were characteristic of an indolizidine moiety [13], further supported by HMBC correlations from H-13 to C-16 and C-18. HMBC correlations from H_2_-14 and H_2_-19 to C-12; from H-13 to C-11; and from H_2_-18 to C-3 established fusion of the indolizidine unit to the lactam-diazepine core at the oxygenated tertiary carbon C-3 and quaternary carbon C-12. Final structural connections were secured by HMBC correlations from H_2_-2 to C-11 and C-13; from H_2_-6 to C-1; and from H_2_-1 to C-4, revealing that the remaining CH_2_-1–CH_2_-2 fragment bridged *N*-5 with C-12 to form a piperidine ring. The fully assembled planar structure proposed of **3** was entirely consistent with the combined HSQC, ^1^H-^1^H COSY, and HMBC data (Fig. 2).

The relative configuration at C-3, C-4, C-12, and C-13 in **3** was determined through NOESY analysis (Fig. 3). Owing to the rigidity of the pentacyclic fused-ring system, the stereochemistry at C-4 and C-12 was constrained to be S* and R*, respectively, with the *N*-5 lone pair adopting an *exo* orientation. Key NOESY correlations for H-4α/H-6α, H-4α/H-8α, and H-8α/H-9α established their pseudo-axial orientations, supporting a twist-chair conformation of the 1,3-diazepine ring. This assignment was further reinforced by additional NOESY correlations, including for H-8β/H-9α, H-8β/H-9β, H-6β/H-7α, and H-6β/H-7β. Within the indolizidine moiety, NOESY correlations for H-14β/H-2β and H-13/H-2β indicated that H-13 adopts an axial (β) orientation, consistent with a 13S* configuration. Furthermore, NOESY correlations among H-13, H-18β, and H-16β demonstrated that these protons are axially oriented, confirming a *trans*-fused indolizidine ring junction. This assignment is in agreement with the characteristic Wenkert-Bohlmann bands observed at 2802 and 2877 cm⁻^1^ in the IR spectrum [14, 15].

**Fig. 5:**
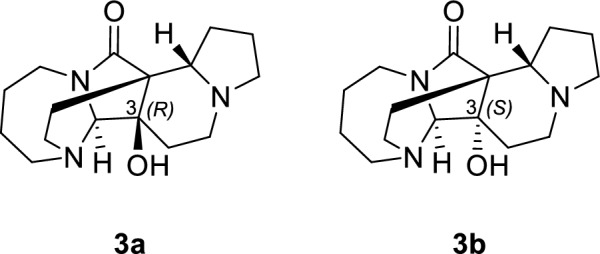
Two possible diastereomers of **3** (**3a** and **3b**) used in GIAO NMR calculations

The absence of NOESY correlations between H-2 and both H-18β and H-19β suggested that the hydroxyl group at C-3 is β-oriented (axial). To confirm this assignment and unambiguously distinguish between the two possible diastereomers, **3a** (3*R*,4*S*,12*R*,13*S*) and **3b** (3*S*,4*S*,12*R*,13*S*) (Fig. 5), gauge-including atomic orbital (GIAO) NMR chemical shift calculations were performed in combination with DP4 + probability analysis, a widely used computational approach for resolving relative configurations of diastereomeric structures [16]. Conformers within a 5 kcal/mol energy window of the global minimum were optimized, Boltzmann-weighted, and averaged. The experimental ^1^H and ^13^C NMR chemical shifts of **3** were then compared with the calculated values for both **3a** and **3b**, and mean absolute errors (MAEs) were obtained (Tables S5 and S6, Supplementary Material). The DP4 + algorithm statistically evaluated the probability for each stereoisomer by integrating deviations from both ^1^H and ^13^C datasets into a Bayesian model [16]. Diastereomer **3a** exhibited significantly lower MAE values (^1^H = 0.12; ^13^C = 2.60) than **3b** (^1^H = 0.28; ^13^C = 4.10). Consistent with these deviations, the DP4 + analysis assigned 100% probability (all data) to **3a** (Fig. S44, Supplementary Material), thereby establishing the relative configuration of **3** as 3R*,4S*,12R*,13S*. The absolute configuration of **3** could not be determined, however, due to the absence of a suitable chromophore for ECD analysis and insufficient materials for chemical derivatization.

Pandazepine D (**4**) was obtained as an UV-inactive brown amorphous, with $[\alpha]^{25}_{\mathrm{D}}-3$ (*c* 0.14, CHCl_3_). The IR spectrum showed absorptions for a lactam carbonyl (1687 cm^−1^; *δ*_C_ 175.9 for C-10) and a hydroxyl group (3408 cm^−1^), consistent with the functional groups observed in **3**. HRDARTMS displayed an [M + H]^+^ peak at *m*/*z* 292.2035, confirming the molecular formula C_16_H_25_N_3_O_2_ and establishing **4** as an isomer of **3**. The ^1^H and ^13^C NMR spectra of **4** (Table 2) closely resembled those of **3**, again showing 25 protons and 16 carbons. However, notable chemical shift differences at C-3 (*δ*_C_ 54.4), C-4 (*δ*_C_ 82.2, *δ*_H_ 3.57), and C-12 (*δ*_C_ 77.1) suggested structural variation around this region. ^1^H-^1^H COSY analysis (Fig. 2) revealed four spin systems identical to those observed in **3**: CH_2_-6–CH_2_-7–CH_2_-8–CH_2_-9, CH-13–CH_2_-14–CH_2_-15–CH_2_-16, and two CH_2_CH_2_ fragments (C-1–C-2 and C-18–C-19). The first partial structure, along with key HMBC correlations from H_2_-9 to C-11; and from H-4 to C-6, C-11 and C-12, established a fused γ-lactam-diazepine ring system analogous to that of **3**. The CH-13–CH_2_-14–CH_2_-15–CH_2_-16 and CH_2_-18–CH_2_-19 fragments were assigned to an indolizidine ring based on HMBC correlations from H_2_-16 to C-13 and C-18; and from H_2_-18 to C-13.

The primary structural difference between **4** and **3** involved the linkage between the indolizidine and lactam rings. HMBC correlations from H_2_-18 to C-12; from H-13 to C-4; and from H_2_-19 to C-11 enabled the assignment of the quaternary carbon at C-3 and the tertiary hydroxyl-bearing carbon at C-12. Attachment of the C-1–C-2 ethylene fragment to *N*-5 and C-3 was confirmed by HMBC correlations from H_2_-1 to C-3, C-4, and C-6; and from H_2_-2 to C-4 and C-13. These spectroscopic data established **4** as a structural isomer of **3**, with the proposed planar structure fully consistent with 2D NMR analysis (Fig. 2).

The relative configuration of **4** was determined through comprehensive NOESY analysis (Fig. 3). As in **3**, NOESY correlations among the pseudo-axial (α) protons of the 1,3-diazepine ring, namely H-4/H-6α, H-6α/H-8α, and H-4/H-9α, indicated a twist-chair conformation, with the *N*-5 lone pair adopting a pseudo-axial (β) orientation. This assignment was supported by additional NOESY correlations, including H-9β/H-7β, H-8β/H-7β, H-8α/H-7α, and H-7α/H-6α. Given the rigidity of the pentacyclic fused-ring system, the stereochemistry at C-4 and C-3 was constrained to S* and R*, respectively. A key NOESY correlation between H-4 and H-13 strongly supported an α-orientation for H-13, consistent with a 13S* configuration*.* Additional NOESY correlations, including H-13/H-16α, H-16α/H-15α, H-16α/H-18α, and H-18α/H-19 (*δ*_H_ 2.24, α), demonstrated that these protons occupy the same α-face of the indolizidine ring, with H-18α and H-13 in axial positions. This arrangement defined a *trans*-fused indolizidine junction, in agreement with the characteristic Wenkert-Bohlmann bands observed at 2798 and 2856 cm^−1^. These data collectively established the relative configuration of **4** as 3R*,4S*,13S*.

**Fig. 6:**
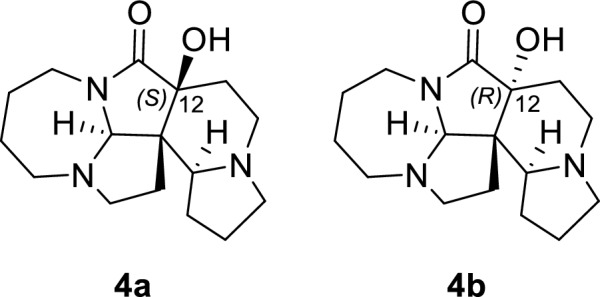
Two possible diastereomers of** 4** (**4a** and **4b**) used in GIAO NMR calculations

Similar to **3**, the relative configuration at C-12 in **4** could not be confidently assigned from NOESY data alone due to overlapping cross-peaks. To resolve this ambiguity, GIAO NMR calculations coupled with DP4 + probability analysis were employed to evaluate two plausible candidate diastereomers, **4a** (3*R,*4*S,*12*S,*13*S*) and **4b** (3*R,*4*S,*12*R,*13*S*) (Fig. 6). The calculated mean absolute errors (MAE) for **4a** (^1^H = 0.11; ^13^C = 2.40) were significantly lower than those for **4b** (^1^H = 0.42; ^13^C = 4.00), demonstrating a much stronger agreement between the experimental and computed NMR chemical shifts (Tables S9 and S10, Supplementary Material). Consistent with these findings, DP4 + analysis assigned 100% probability (all data) to **4a** (Fig. S45, Supplementary Material), thereby establishing the relative configuration of **4** as 3R*,4S*,12S*,13S*. As with **3**, the absolute configuration could not be determined due to insufficient material and the absence of a suitable chromophore for ECD analysis.

**Fig. 7:**
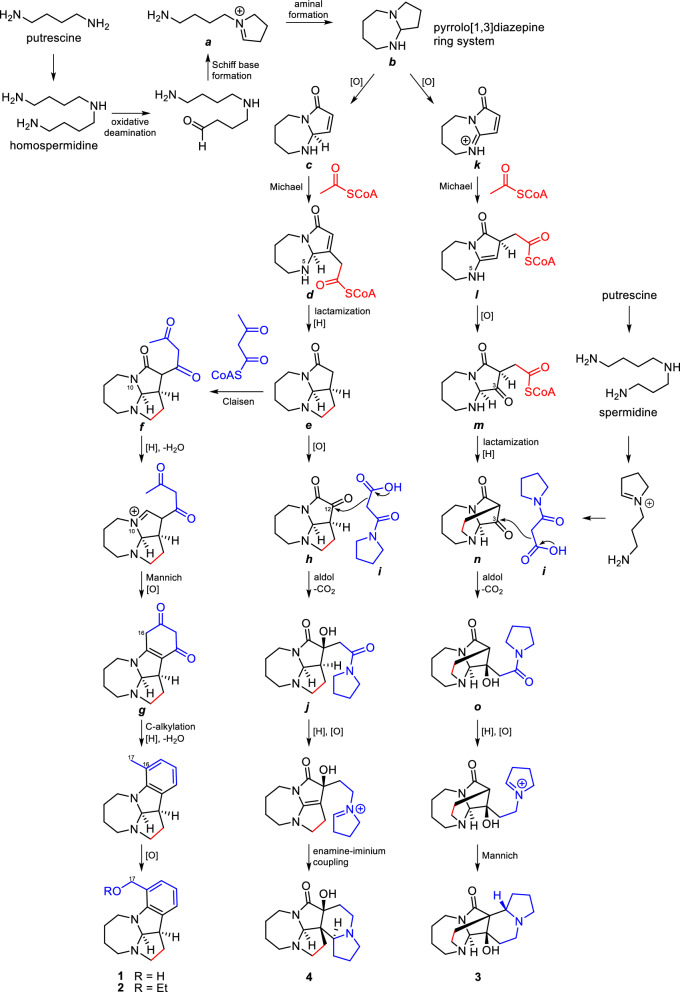
Plausible biosynthetic pathway for compounds **1**–**4**

Pandazepines A and B (**1** and **2**) represent the first examples of tetracyclic pyrrolidinoindoline-1,3-diazepine alkaloids, while pandazepines C and D (**3** and **4**) possess unusual pentacyclic fused-ring systems in which 1,3-diazepine, γ-lactam, and indolizidine units are linearly fused. These unprecedented structural architectures among natural products necessitate some speculation regarding their biosynthetic origin. The presence of pyrrolidine moieties and an N(CH_2_)_4_N fragment suggests that putrescine, spermidine, or related polyamine alkaloids may serve as plausible biosynthetic precursors (Fig. 7). Such polyamines are widely distributed in plants and other organisms [17].

A putative biosynthetic pathway may originate from putrescine, which is converted to homospermidine, followed by oxidative deamination and intramolecular Schiff-base formation to generate the pyrrolidine iminium ion ***a*** [18] (Fig. 7). Subsequent intramolecular aminal formation would furnish pyrrolo[1,3]diazepine ***b***. This bicyclic intermediate may then undergo a series of oxidative functionalization steps to generate the Michael acceptor intermediate ***c***. Nucleophilic conjugate addition of an acetyl-CoA unit to ***c*** would afford the Michael adduct ***d***.

Lactamization of ***d***, involving the newly installed acyl group and *N*-5, would give rise to the tricyclic intermediate ***e***, which may subsequently undergo a Claisen reaction with an acetoacetyl-CoA unit to furnish the 1,3-diketo intermediate ***f***. Conversion of the *N*-10 lactam function into an iminium ion would enable an intramolecular Mannich reaction, followed by oxidative dehydrogenation to afford the tetracyclic intermediate ***g***. Subsequent *C*-methylation at C-16 by *S*-adenosylmethionine (SAM), aromatization, and benzylic oxidation at C-17 would give rise to the core skeleton of compounds **1** and **2**.

Diverging from this pathway, oxidation of intermediate ***e*** may afford the tricyclic 12-oxo intermediate ***h***, which could undergo an aldol reaction with the pyrrolidine acid ***i*** to furnish the aldol adduct ***j***. The pyrrolidine acid ***i*** is postulated to arise from putrescine via a sequence of transformations involving oxidative deamination, intramolecular Schiff-base formation, iminium ion reduction, a second oxidative deamination, and final oxidation of the aldehyde to the corresponding carboxylic acid. Subsequent amide reduction and oxidative transformations of ***j*** would generate an enamine-iminium ion intermediate, which may undergo intramolecular enamine-iminium coupling to furnish compound **4**.

In a parallel branch of the pathway, oxidative functionalization of the bicyclic intermediate may instead generate the Michael acceptor ***k***. Nucleophilic conjugate addition of an acetyl-CoA unit to ***k*** would afford the Michael adduct ***l***, which may be oxidized to the 3-oxo intermediate ***m***. Subsequent lactamization and lactam reduction would furnish the tricyclic 3-oxo intermediate ***n***. An aldol reaction between ***n*** and the pyrrolidine acid ***i*** would yield the aldol adduct ***o***, which may undergo amide reduction and oxidation to generate an iminium ion intermediate. A final intramolecular Mannich reaction would then furnish compound **3**.

## Conclusion

Alkaloids bearing a diazepine ring as their core structural motif are exceedingly rare in plants [19]. In contrast, seven-membered nitrogen heterocycles in plant secondary metabolites are more commonly encountered as azepine rings embedded within indole- or pyrrole-derived frameworks [20, 21]. In this context, the discovery of pandazepines A–D (**1**–**4**), the first diazepine-containing alkaloids from *P. yvanii*, represents a chemically distinctive and biosynthetically intriguing discovery. These compounds feature unprecedented tetracyclic (**1** and **2**) and pentacyclic (**3** and **4**) fused-ring systems that depart fundamentally from the previously established *Pandanus* alkaloid chemotype. Alkaloids reported from this genus are predominantly characterized by 9- or 18-carbon skeletons bearing a terminal γ-alkylidene α,β-unsaturated γ-lactone or γ-lactam moiety, with some further incorporating pyrrolidine, piperidine, or indolizidine rings. In sharp contrast, the architectures of **1**–**4** establish a structurally distinct class of alkaloids within the genus. This pronounced structural divergence not only broadens the chemotaxonomic profile of *Pandanus* but also suggests that *P. yvanii* may employ distinct enzymatic pathways capable of assembling complex polycyclic nitrogen heterocycles via biosynthetic routes fundamentally distinct from those operating in other *Pandanus* species.

## Experimental

### General experimental procedures

^1^H and ^13^C NMR spectra were recorded in CDCl_3_ or CD_3_OD on a Bruker Avance III 600 MHz spectrometer using TMS as the internal standard. Analysis of spectra was carried out using MestReNova Software (v14.0.0-23239). HRDARTMS were acquired on a JEOL Accu TOF-DART mass spectrometer. IR spectra were recorded on a PerkinElmer Spectrum 400 FT-IR/FT-FIR spectrophotometer. UV spectra were measured on a PerkinElmer Lambda 35 UV/Vis spectrophotometer. Optical rotations were measured on a JASCO P-1020 automatic digital polarimeter. ECD spectra were recorded on a JASCO J-815 circular dichroism spectrometer.

### Plant material

The whole plants of *P. yvanii* were collected in June 2023 from Hulu Langat, Selangor, Malaysia, and identified by K. T. Yong (Institute of Biological Sciences, Universiti Malaya). A voucher specimen (KLU50160) was deposited in the Herbarium, Universiti Malaya.

### Extraction and isolation

The leaves were separated from the stems and dried overnight in an oven. The dried leaves (7 kg) were ground into a fine powder with a Retsch TM SM 100 rotor beater mill and macerated with 95% EtOH (40 L × 5, overnight). The solvent was removed in vacuo to give the crude extract. The concentrated ethanolic extract was added into 3% tartaric acid solution with vigorous stirring. The acidic solution was vacuum filtered through a Kieselguhr layer to remove insoluble non-alkaloidal material. The filtrate was basified to pH 10 with 28% w/v ammonia solution, and the liberated alkaloids were exhaustively extracted with CHCl_3_, washed with distilled water, dried over Na_2_SO_4_, and concentrated under reduced pressure. This partitioning process was repeated three times to furnish the crude alkaloid mixture (10.1 g).

The crude alkaloid (10.1 g) was fractionated by dry column vacuum chromatography elution (silica gel 60, Et_2_O/hexane 1:1 v/v and Et_2_O/MeOH 4:1 v/v, NH_3_-saturated) to furnish eleven main fractions (Fr. 1–11). Fraction 3 (632.2 mg) was re-chromatographed using centrifugal preparative thin layer chromatography (Chromatotron, silica gel 60, hexane/Et_2_O 1:1 v/v; Et_2_O/MeOH 4:1 v/v, NH_3_-saturated) to give five subfractions (Fr. 3a–3e). Repeated purification of Fr. 3c (20.6 mg) by centrifugal thin layer chromatography (hexane/Et_2_O 1:4 v/v; 100% Et_2_O, NH_3_-saturated) yielded **2** (3.4 mg). Compounds **3** (4.2 mg) and **4** (3.9 mg) were obtained by repeated centrifugal thin layer chromatography (hexane/CHCl_3_ 1:4 v/v; 100% CHCl_3_, NH_3_-saturated) of Fr. 3d (60.9 mg). Fraction 5 (483.5 mg) was separated by centrifugal thin layer chromatography (100% Et_2_O; Et_2_O/MeOH, 9:1 v/v, NH_3_-saturated) to give seven subfractions (Fr. 5a–5 g). Further purification of Fr. 5 g (22.2 mg) by semi-preparative reverse phase HPLC (Waters XBridge Prep C18 Column, 5 µm, 10 × 10 mm) under gradient elution [MeOH/H_2_O 30:70, 90:10 v/v (0.1% NH_3_), 3 mL/min for 30 min, 30 °C] provided **1** (5.5 mg).

### Spectroscopic data of compounds

#### Pandazepine A (1)

Brown amorphous; $[\alpha]^{25}_{\mathrm{D}}-12$ (*c* 0.16, CHCl_3_); UV (CH_3_CN) *λ*_max_ (log *ε*) 212 (4.16), 264 (3.79), 312 (3.36) nm; IR *ν*_max_ 3362 (br), 2929, 2849, 1663, 1627, 1450, 1414 cm^−1^; ^1^H NMR and ^13^C NMR data, see Table 1; ECD (CH_3_CN) λ_max_ (Δε) 206 (–0.9), 216 (+ 1.1), 285 (–0.1) nm; HRDARTMS *m/z* 245.1663 [M + H]^+^ (calcd for C_15_H_21_N_2_O^+^, 245.1654).

#### Pandazepine B (2)

Brown amorphous; $[\alpha]^{25}_{\mathrm{D}}-5$ (*c* 0.15, CHCl_3_); UV (CH_3_CN) *λ*_max_ (log *ε*) 210 (4.02), 264 (3.60), 313 (3.15) nm; IR *ν*_max_ 2932, 2809, 2134, 1974, 1734, 1716, 1473, 1456, 1250, 1200, 1176, 1066, 1037 cm^−1^; ^1^H NMR and ^13^C NMR data, see Table 1; ECD (CH_3_CN) λ_max_ (Δε) 200 (–2.7), 217 (+ 1.3), 281 (–0.1) nm; HRDARTMS *m/z* 273.1960 [M + H]^+^ (calcd for C_17_H_25_N_2_O^+^, 273.1967).

#### Pandazepine C (3)

Brown amorphous; $[\alpha]^{25}_{\mathrm{D}}-9$ (*c* 0.14, CHCl_3_); IR *ν*_max_ 3385 (br), 2931, 2877, 2802, 1698, 1432, 1280, 1253, 1221, 1183, 1139, 1023 cm^−1^; ^1^H NMR and ^13^C NMR data, see Table 2; HRDARTMS *m/z* 292.2039 [M + H]^+^ (calcd for C_16_H_26_N_3_O_2_^+^, 292.2025).

#### Pandazepine D (4)

Brown amorphous; $[\alpha]^{25}_{\mathrm{D}}-3$ (*c* 0.14, CHCl_3_); IR *ν*_max_ 3408 (br), 2955, 2924, 2856, 2798, 1687, 1131 cm^−1^; ^1^H NMR and ^13^C NMR data, see Table 2; HRDARTMS *m/z* 292.2035 [M + H]^+^ (calcd for C_16_H_26_N_3_O_2_^+^, 292.2025).

### Computational methods

Structures of compounds **1**–**4** were initially built using GaussView6 and then optimized at the semi-empirical PM6 level of theory. Conformational searches were performed using Spartan’14 software with the MMFF94 force field. Conformers within a 5 kcal/mol energy window of the global minimum were subjected to DFT-level geometry optimization and frequency calculations at the B3LYP/6-31G(d) level using Gaussian 09 software. For compounds **1** and **2**, TDDFT-ECD calculations were performed on the optimized conformers at the ωB97X-D/def2-TZVP level (imported from the Basis Set Exchange) using a PCM solvation model for CH_3_CN. The ECD spectrum for each optimized conformer was weighted by Boltzmann distribution after UV correction, and the overall ECD spectra were simulated by SpecDis v1.71 software with applied Gaussian bandwidth of 0.12 − 0.40 eV and UV shifts of + 2 to 20 nm.

For compounds **3** and **4**, GIAO NMR calculations were performed on the optimised conformers at the mPW1PW91/6–31 + G(d,p) level with a PCM solvation model for CHCl_3_. The Boltzmann-averaged magnetic shielding tensors were converted to chemical shifts (ppm) using TMS as the reference standard (^1^H = 31.5572; ^13^C = 196.5384). Mean absolute errors (MAE) were calculated as the average deviation between experimental and calculated chemical shifts. DP4 + probability analysis was performed using the Excel spreadsheet provided by the Sarotti group [16].

## Supplementary Information

Additional file 1.

## Data Availability

The datasets generated during and/or analyzed during the current study are available from the corresponding author on reasonable request.

## References

[1] Beentjie HJ, Callmander MW. Pandanaceae. In: Kiew R, Chung RCK, Saw LG, editors. F Flora of Peninsular Malaysia. Series II: seed plants (Malayan Forest Records 49), vol. 10. Forest Research Institute Malaysia; 2023. p. 209–11.

[2] Nonato MG, Garson MJ, Truscott RJW, Carver JA. Structural characterization of piperidine alkaloids from *Pandanus amaryllifolius* by inverse-detected 2D NMR techniques. Phytochemistry. 1993. 10.1016/S0031-9422(00)90735-0.

[3] Takayama H, Ichikawa T, Kuwajima T, Kitajima M, Seki H, Aimi N, et al. Structure characterization, biomimetic total synthesis, and optical purity of two new pyrrolidine alkaloids, Pandamarilactonine-A and -B, isolated from *Pandanus amaryllifolius* Roxb. J Am Chem Soc. 2000. 10.1021/ja0009929.

[4] Takayama H, Ichikawa T, Kitajima M, Nonato M, Aimi N. Isolation and structure elucidation of two new alkaloids, Pandamarilactonine-C and -D, from *Pandanus amaryllifolius* and revision of relative stereochemistry of Pandamarilactonine-A and -B by total synthesis. Chem Pharm Bull (Tokyo). 2002. 10.1248/cpb.50.1303. 12237561 10.1248/cpb.50.1303

[5] Tan MA, Kitajima M, Kogure N, Nonato MG, Takayama H. New pyrrolidine alkaloids from the roots of *Pandanus amaryllifolius*. Tetrahedron Lett. 2010. 10.1016/j.tetlet.2010.05.150.

[6] Tan MA, Kitajima M, Kogure N, Nonato MG, Takayama H. Isolation of Pandamarilactonine-H from the roots of *Pandanus amaryllifolius* and synthesis of epi-Pandamarilactonine-H. J Nat Prod. 2010. 10.1021/np1003998. 20701299 10.1021/np1003998

[7] Tan MA, Kitajima M, Kogure N, Nonato MG, Takayama H. Isolation and total syntheses of two new alkaloids, Dubiusamines-A, and -B, from *Pandanus dubius*. Tetrahedron. 2010. 10.1016/j.tet.2010.02.073.

[8] Cheng YB, Tsai YH, Lo IW, Haung CC, Tsai YC, Beerhues L, et al. Pandalisines A and B, novel indolizidine alkaloids from the leaves of *Pandanus utilis*. Bioorg Med Chem Lett. 2015. 10.1016/j.bmcl.2015.07.041. 26277406 10.1016/j.bmcl.2015.07.041

[9] Cheng YB, Hu HC, Tsai YC, Chen SL, El-Shazly M, Nonato MG, et al. Isolation and absolute configuration determination of alkaloids from *Pandanus amaryllifolius*. Tetrahedron. 2017. 10.1016/j.tet.2017.05.002.

[10] Nonato MG, Takayama H, Garson MJ. Chapter 4: *Pandanus* alkaloids chemistry and biology. In: Cordell GA, editor. The alkaloids chemistry and biology, vol. 66. Academic Press: New York; 2008. p. 215–49. 10.1016/s1099-4831(08)00204-6 19025100

[11] Wang YH, Long CL, Yang FM, Wang X, Sun QY, Wang HS, et al. Pyrrolidinoindoline alkaloids from *Selaginella moellendorfii*. J Nat Prod. 2009. 10.1021/np9001515. 19422203 10.1021/np9001515

[12] De Carvalho AR, Vieira IJC, De Carvalho MG, Braz-Filho R, Lima MAS, Ferreira RO, et al. 13C-NMR spectral data of alkaloids isolated from *Psychotria* species (Rubiaceae). Molecules. 2017. 10.3390/molecules22010103. 28085077 10.3390/molecules22010103 PMC6155581

[13] Zhang J, Morris-Natschke SL, Ma D, Shang XF, Yang CJ, Liu YQ, et al. Biologically active indolizidine alkaloids. Med Res Rev. 2021. 10.1002/med.21747. 33128409 10.1002/med.21747

[14] Rosen WE. Rauwolfia alkaloids—XLII methyl reserpate, an isomer of methyl reserpate. Part 4. Infrared spectra and configuration at C-3. Tetrahedron Lett. 1961. 10.1016/S0040-4039(00)71758-8.

[15] Wenkert E, Guo M, Pestchanker MJ, Shi YJ, Vankar YD. Preparation and H(3) isomerization of C(15)-substituted deplancheine derivatives. Synthesis of geissoschizol and geissoschizine. J Org Chem. 1989. 10.1021/jo00266a032.

[16] Grimblat N, Zanardi MM, Sarotti AM. Beyond DP4: an improved probability for the stereochemical assignment of isomeric compounds using quantum chemical calculations of NMR shifts. J Org Chem. 2015. 10.1021/acs.joc.5b02396. 26580165 10.1021/acs.joc.5b02396

[17] Zhang L, Gu C, Liu J. Nature spermidine and spermine alkaloids: occurrence and pharmacological effects. Arab J Chem. 2022. 10.1016/j.arabjc.2022.104367.36189434

[18] Brauch S, Veldmate WS, Rutjes FPJT. Chapter 11: Ornithine and lysine alkaloids. In: Zografos AL, editor. From biosynthesis to total synthesis: strategies and tactics for natural products. Wiley: Hoboken; 2016. p. 383–430.

[19] Malki Y, Martinez J, Masurier N. 1,3-Diazepine: a privileged scaffold in medicinal chemistry. Med Res Rev. 2021. 10.1002/med.21795. 33645848 10.1002/med.21795

[20] Klein-Júnior LC, Cretton S, Vander Heyden Y, Gasper AL, Nejad-Ebrahimi S, Christen P, et al. Bioactive azepine-indole alkaloids from *Psychotria nemorosa*. J Nat Prod. 2020. 10.1021/acs.jnatprod.9b00469. 32150413 10.1021/acs.jnatprod.9b00469

[21] Xu Y, Liang J, Yan Y, Sun D, Li H, Chen L. The structure and bioactivities of *Stemona* alkaloids and alkaloids with [1,2-α] azepine nucleus (2009–2021). Phytochem Rev. 2024. 10.1007/s11101-023-09900-0.

