# Supplementary material for: Isolation and structural characterization of polycyclic diazepine alkaloids from Pandanus yvanii
Source: Nat Prod Bioprospect. 2026 Jul 13;16(1):79. doi: 10.1007/s13659-026-00632-0 (PMC13357470; doi:10.1007/s13659-026-00632-0)
Supplement: Supplementary file 1 — Additional file 1. [file 13659_2026_632_MOESM1_ESM.pdf]

Supplementary Material

Isolation and structural characterization of polycyclic diazepine alkaloids from *Pandanus yvanii*

Jin-Ning Chu[1], Premanand Krishnan[1], Kien-Thai Yong[3], Yun-Yee Low[2], and Kuan-Hon Lim[1,*]

[1] *School of Pharmacy, University of Nottingham Malaysia, Jalan Broga, 43500 Semenyih, Selangor, Malaysia.*

[2] *Department of Chemistry, Faculty of Science, Universiti Malaya, 50603 Kuala Lumpur, Malaysia.*

[3] *Institute of Biological Sciences, Faculty of Science, Universiti Malaya, 50603 Kuala Lumpur, Malaysia.*

* Corresponding author.

E-mail address: KuanHon.Lim@nottingham.edu.my (Lim K-H)

Table of Contents

Figure S1. ^{1}H NMR Spectrum of Pandazepine A (**1**) (CD_3OD, 600 MHz)................................4

Figure S2. ^{13}C NMR Spectrum of Pandazepine A (**1**) (CD_3OD, 150 MHz)...............................5

Figure S3. HSQC Spectrum of Pandazepine A (**1**) CD_3OD, 600 MHz)....................................6

Figure S4. COSY Spectrum of Pandazepine A (**1**) (CD_3OD, 600 MHz) 7

Figure S5. HMBC Spectrum of Pandazepine A (**1**) (CD_3OD, 600 MHz) 8

Figure S6. NOESY Spectrum of Pandazepine A (**1**) (CD_3OD, 600 MHz).................................9

Figure S7. NOESY Spectrum of Pandazepine A (**1**) (CD_3OD, 600 MHz).................................10

Figure S8. NOESY Spectrum of Pandazepine A (**1**) (CD_3OD, 600 MHz).................................11

Figure S9. HRDARTMS of Pandazepine A (**1**)...12

Figure S10. IR and UV Spectra of Pandazepine A (**1**) 13

Figure S11. ^{1}H NMR Spectrum of Pandazepine B (**2**) ($CDCl_3$, 600 MHz)................................14

Figure S12. ^{13}C NMR Spectrum of Pandazepine B (**2**) ($CDCl_3$, 150 MHz)...............................15

Figure S13. HSQC Spectrum of Pandazepine B (**2**) ($CDCl_3$, 600 MHz)....................................16

Figure S14. COSY Spectrum of Pandazepine B (**2**) ($CDCl_3$, 600 MHz)....................................17

Figure S15. HMBC Spectrum of Pandazepine B (**2**) ($CDCl_3$, 600 MHz) 18

Figure S16. NOESY Spectrum of Pandazepine B (**2**) ($CDCl_3$, 600 MHz).................................19

Figure S17. NOESY Spectrum of Pandazepine B (**2**) ($CDCl_3$, 600 MHz).................................20

Figure S18. HRDARTMS of Pandazepine B (**2**) 21

Figure S19. IR and UV Spectra of Pandazepine B (**2**)...22

Figure S20. ^{1}H NMR Spectrum of Pandazepine C (**3**) ($CDCl_3$, 600 MHz)................................23

Figure S21. ^{13}C NMR Spectrum of Pandazepine C (**3**) ($CDCl_3$, 150 MHz)...............................24

Figure S22. HSQC Spectrum of Pandazepine C (**3**) ($CDCl_3$, 600 MHz)....................................25

Figure S23. COSY Spectrum of Pandazepine C (**3**) ($CDCl_3$, 600 MHz)....................................26

Figure S24. HMBC Spectrum of Pandazepine C (**3**) ($CDCl_3$, 600 MHz) 27

Figure S25. NOESY Spectrum of Pandazepine C (**3**) ($CDCl_3$, 600 MHz).................................28

Figure S26. NOESY Spectrum of Pandazepine C (**3**) ($CDCl_3$, 600 MHz).................................29–30

Figure S27. NOESY Spectrum of Pandazepine C (**3**) ($CDCl_3$, 600 MHz).................................31

Figure S28. 1D NOESY Spectrum of Pandazepine C (**3**) ($CDCl_3$, 600 MHz)............................32

Figure S29. 1D NOESY Spectrum of Pandazepine C (**3**) ($CDCl_3$, 600 MHz)............................33

Figure S30. HRDARTMS of Pandazepine C (**3**)...34

Figure S31. IR Spectrum of Pandazepine C (**3**)...35

Figure S32. ^{1}H NMR Spectrum of Pandazepine D (**4**) ($CDCl_3$, 600 MHz)................................36

Figure S33. ^{13}C NMR Spectrum of Pandazepine D (**4**) ($CDCl_3$, 150 MHz)...............................37

Figure S34. HSQC Spectrum of Pandazepine D (**4**) ($CDCl_3$, 600 MHz) 38
Figure S35. COSY Spectrum of Pandazepine D (**4**) ($CDCl_3$, 600 MHz) 39
Figure S36. HMBC Spectrum of Pandazepine D (**4**) ($CDCl_3$, 600 MHz) 40
Figure S37. HMBC Spectrum of Pandazepine D (**4**) ($CDCl_3$, 600 MHz) 41
Figure S38. NOESY Spectrum of Pandazepine D (**4**) ($CDCl_3$, 600 MHz)..................................42
Figure S39. NOESY Spectrum of Pandazepine D (**4**) ($CDCl_3$, 600 MHz)..................................43
Figure S40. HRDARTMS of Pandazepine D (**4**)..44
Figure S41. IR Spectrum of Pandazepine D (**4**)...45
Figure S42. Three Lowest-energy Conformers Calculated at the B3LYP/6-31G(d) Level for (3*R*,4*S*)–**1** 46
Table S1. DFT B3LYP/6-31G(d) Atomic Cartesian Coordinates (Å) for the Lowest-Energy Conformer of (3*R*,4*S*)–**1**..47
Figure S43. Three Lowest-energy Conformers Calculated at the B3LYP/6-31G(d) Level for (3*R*,4*S*)–**2** 48
Table S2. DFT B3LYP/6-31G(d) Atomic Cartesian Coordinates (Å) for the Lowest-Energy Conformer of (3*R*,4*S*)–**2**..49–50
Table S3. DFT B3LYP/6-31+G(d,p) Atomic Cartesian Coordinates (Å) for the Lowest-energy Conformer of **3a** (3*R*,4*S*,12*R*,13*S*) 51–52
Table S4. DFT B3LYP/6-31+G(d,p) Atomic Cartesian Coordinates (Å) for the Lowest-energy Conformer of **3b** (3*S*,4*S*,12*R*,13*S*) 53
Table S5. Experimental and calculated ^{1}H NMR chemical shift (in ppm) of **3**, **3a**, and **3b**........54
Table S6. Experimental and Calculated ^{13}C NMR Chemical Shift (in ppm) of **3**, **3a**, and **3b** 55
Figure S44. DP4+ probability analysis of ^{1}H and ^{13}C NMR chemical shifts of isomers **3a** and **3b** 55
Table S7. DFT B3LYP/6-31+G(d,p) Atomic Cartesian Coordinates (Å) for the Lowest-energy Conformer of **4a** (3*R*,4*S*,12*S*,13*S*)...56–57
Table S8. DFT B3LYP/6-31+G(d,p) Atomic Cartesian Coordinates (Å) for the Lowest-energy Conformer of **4b** (3*R*,4*S*,12*R*,13*S*)...58
Table S9. Experimental and Calculated ^{1}H NMR Chemical Shift (in ppm) **4**, **4a**, and **4b**..........59
Table S10. Experimental and Calculated ^{13}C NMR Chemical Shift (in ppm) **4**, **4a**, and **4b**.......60
Figure S45. DP4+ probability analysis of ^{1}H and ^{13}C NMR chemical shifts of isomers **4a** and **4b** 60

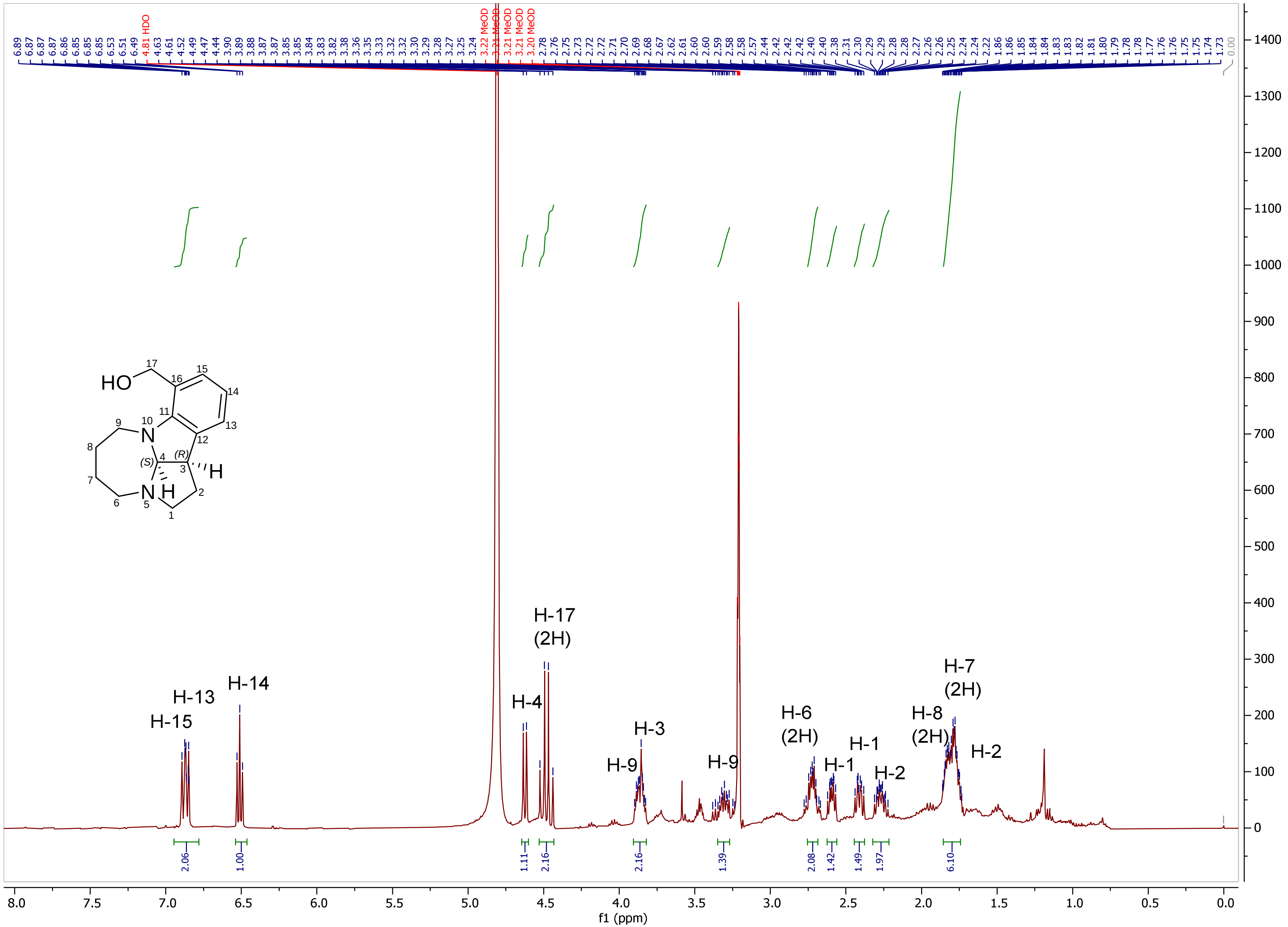

Figure S1. ^{1}H NMR Spectrum of Pandazepine A (**1**) (CD_3OD, 600 MHz)

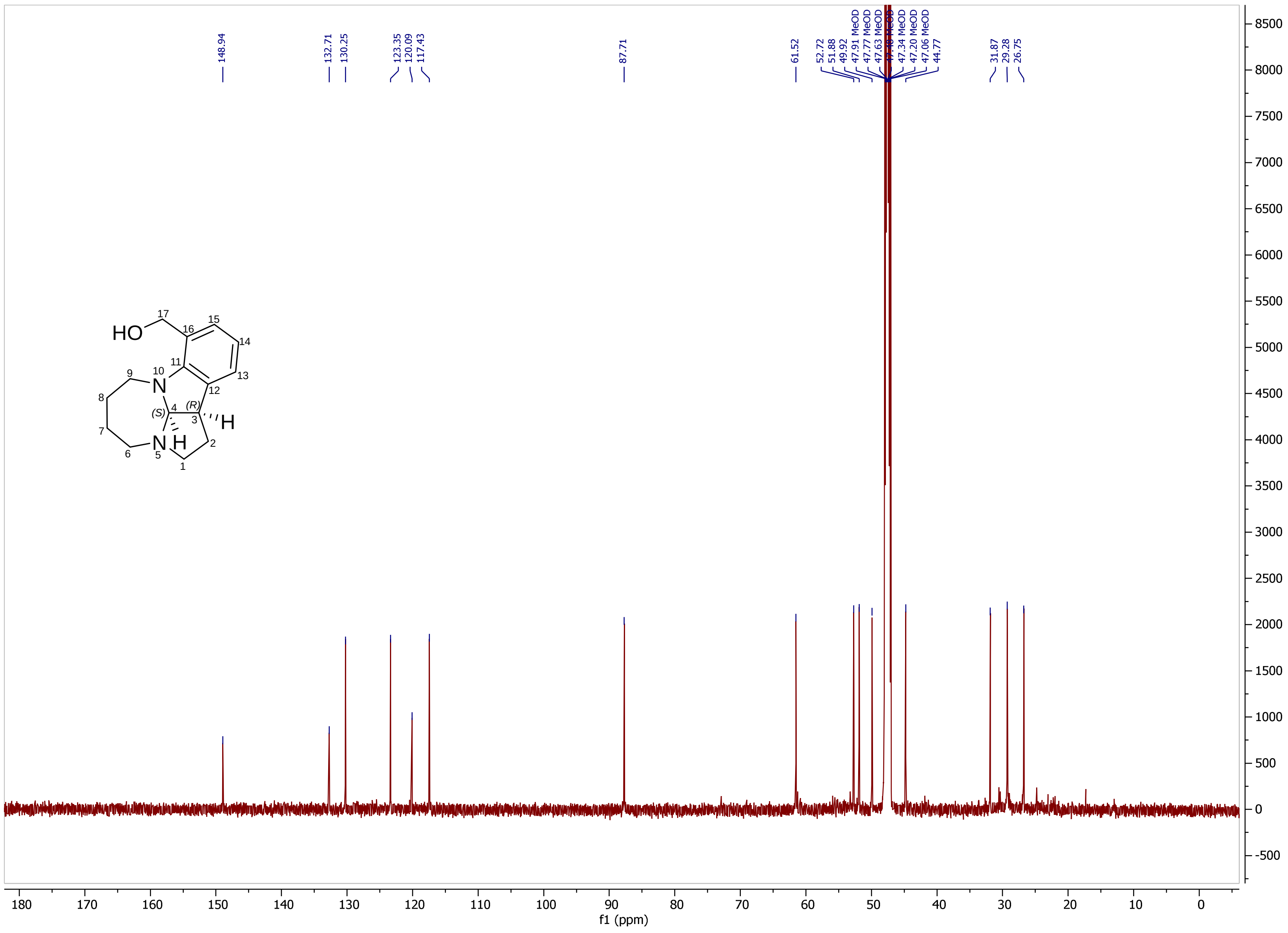

Figure S2. ^{13}C NMR Spectrum of Pandazepine A (**1**) (CD_3OD, 150 MHz)

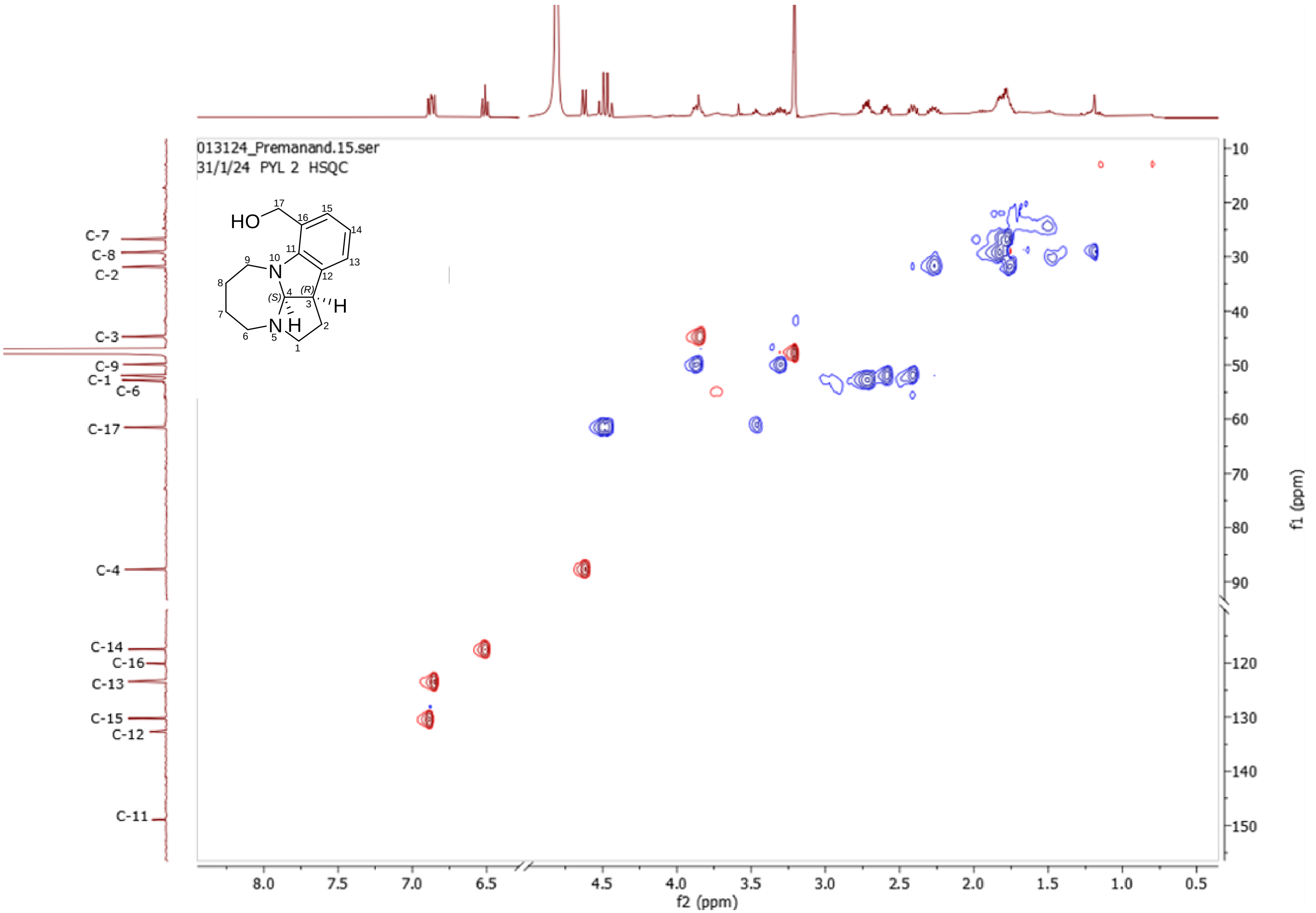

Figure S3. HSQC Spectrum of Pandazepine A (**1**) (CD_3OD, 600 MHz)

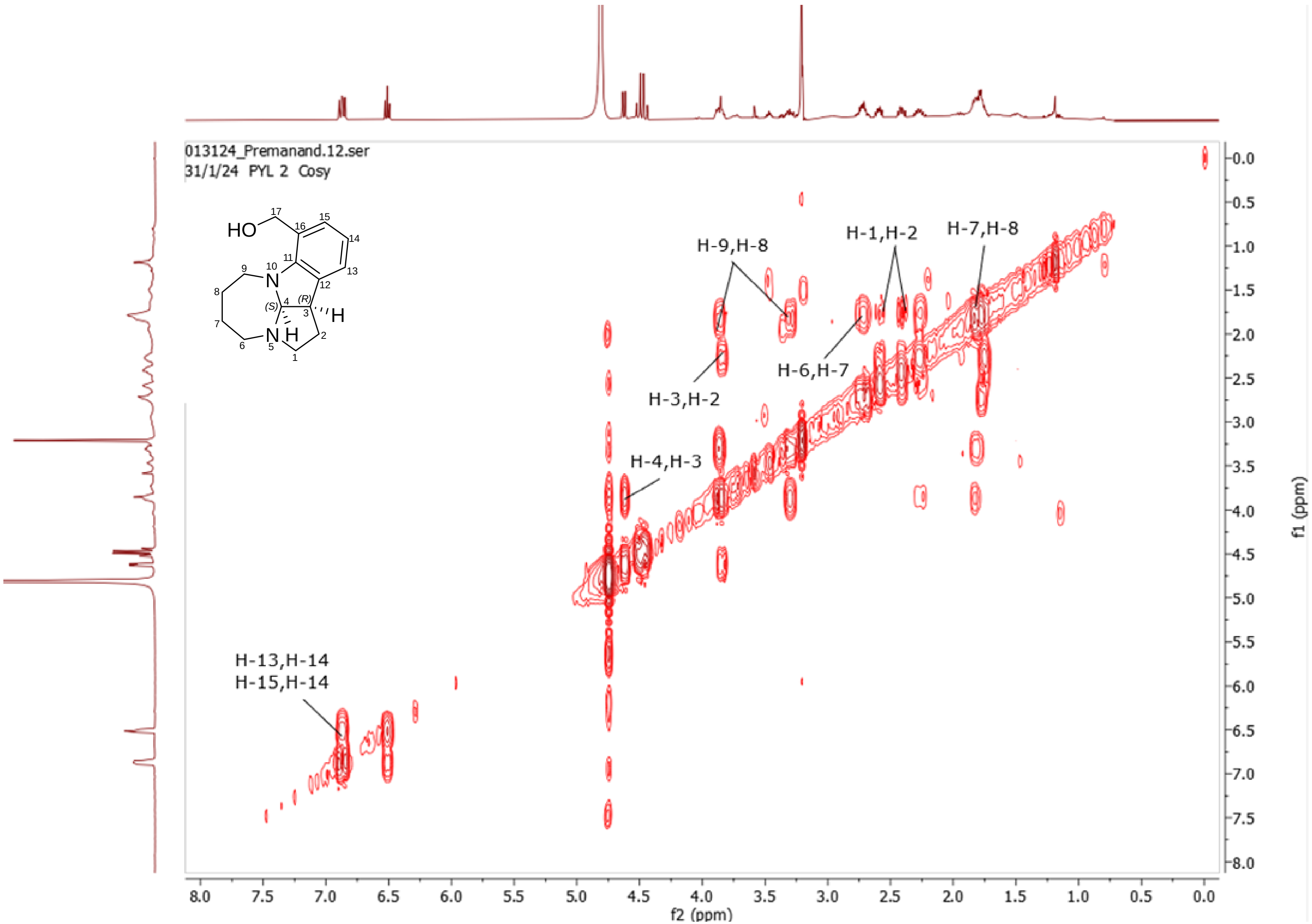

Figure S4. COSY Spectrum of Pandazepine A (**1**) (CD_3OD, 600 MHz)

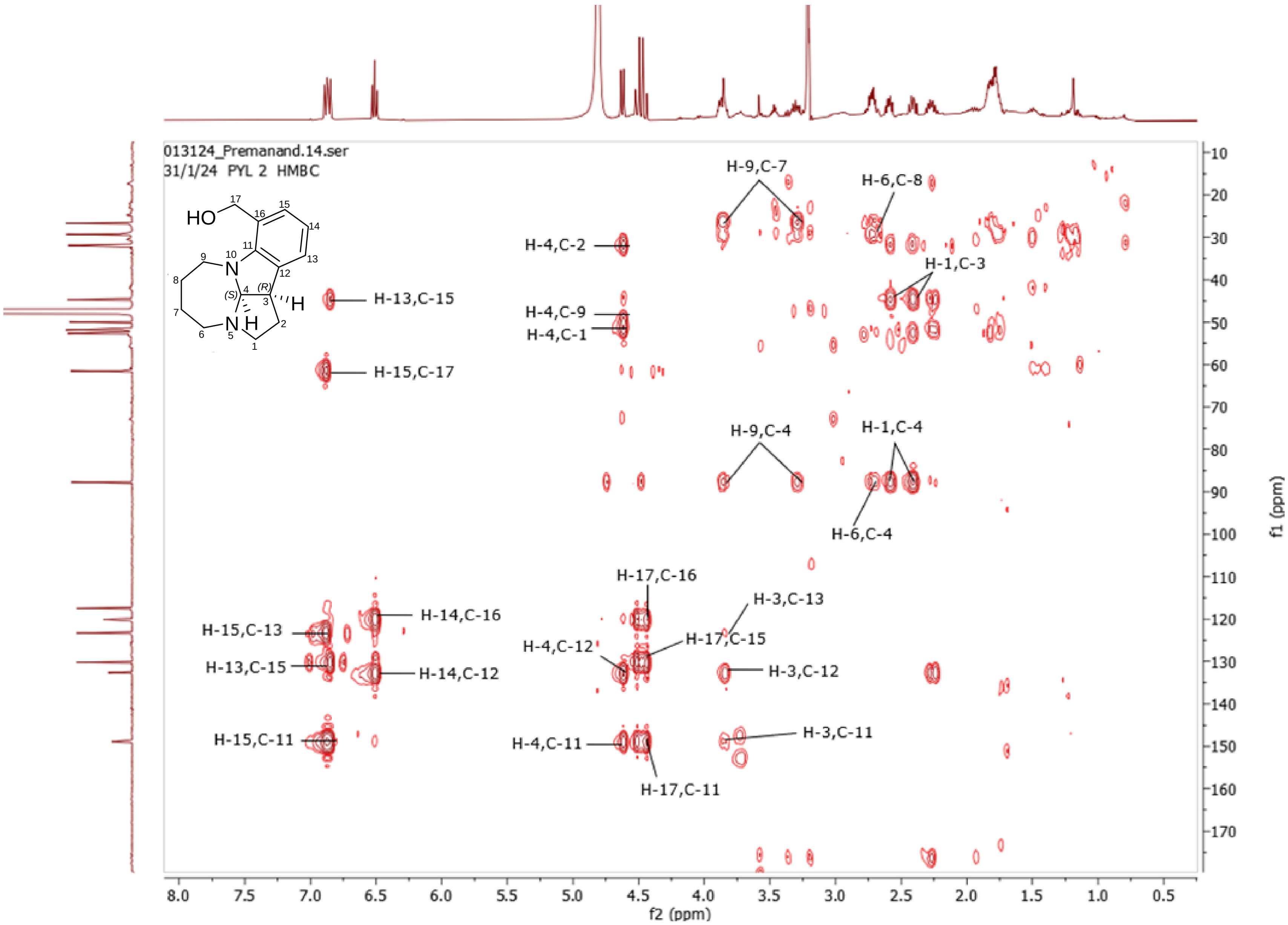

Figure S5. HMBC Spectrum of Pandazepine A (**1**) (CD_3OD, 600 MHz)

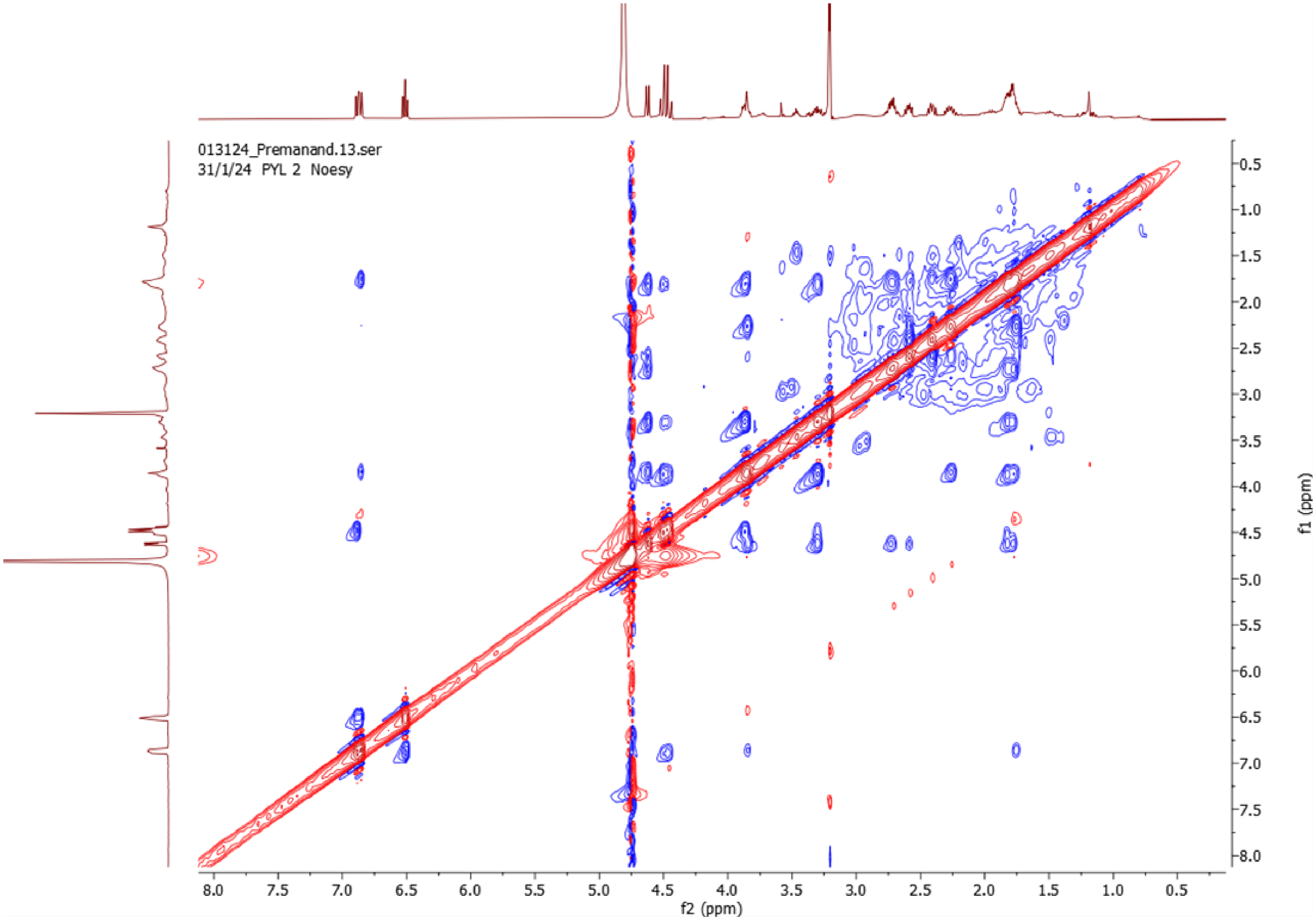

Figure S6. NOESY Spectrum of Pandazepine A (**1**) (CD_3OD, 600 MHz)

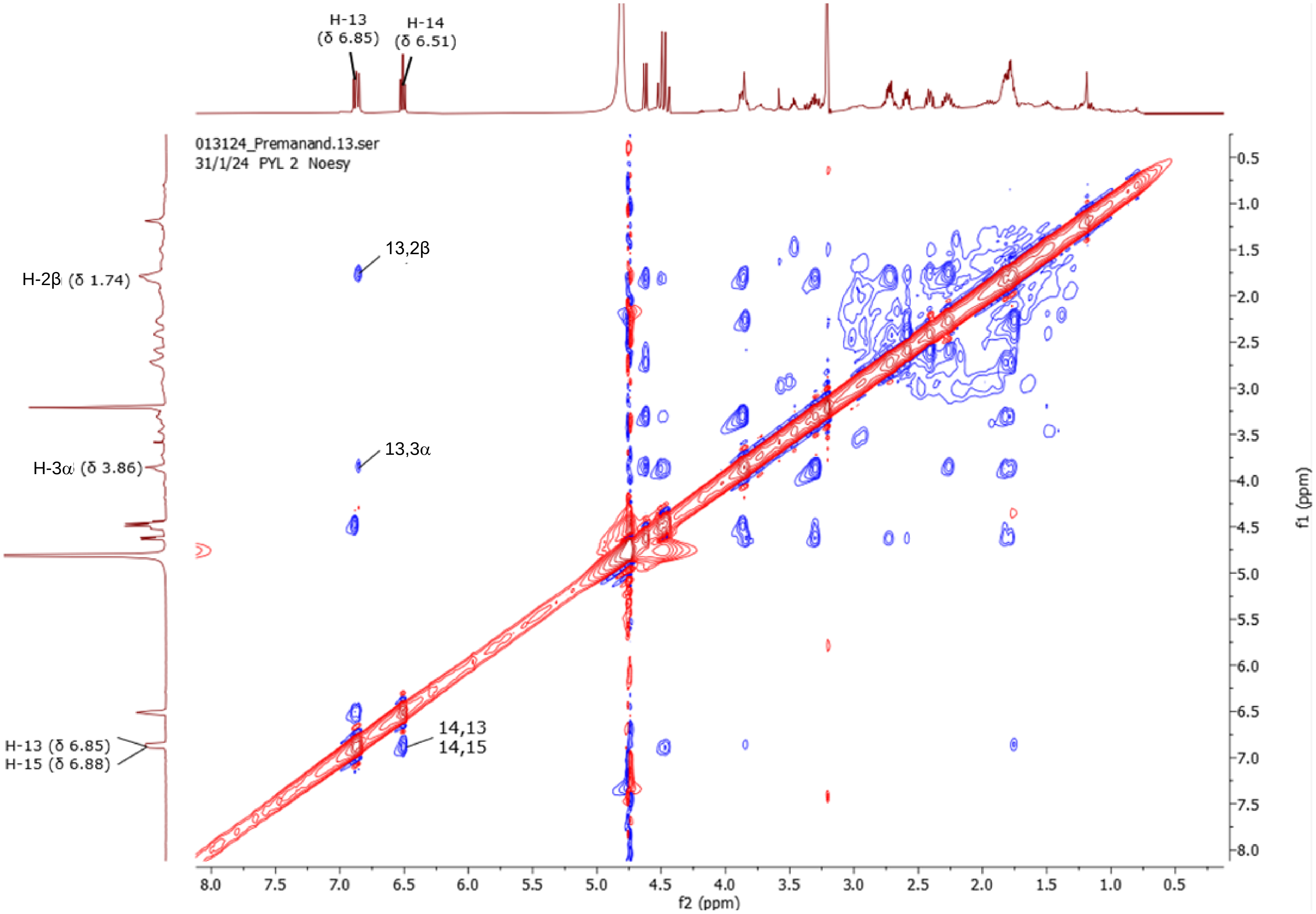

Figure S7. NOESY Spectrum of Pandazepine A (**1**) (CD_3OD, 600 MHz)

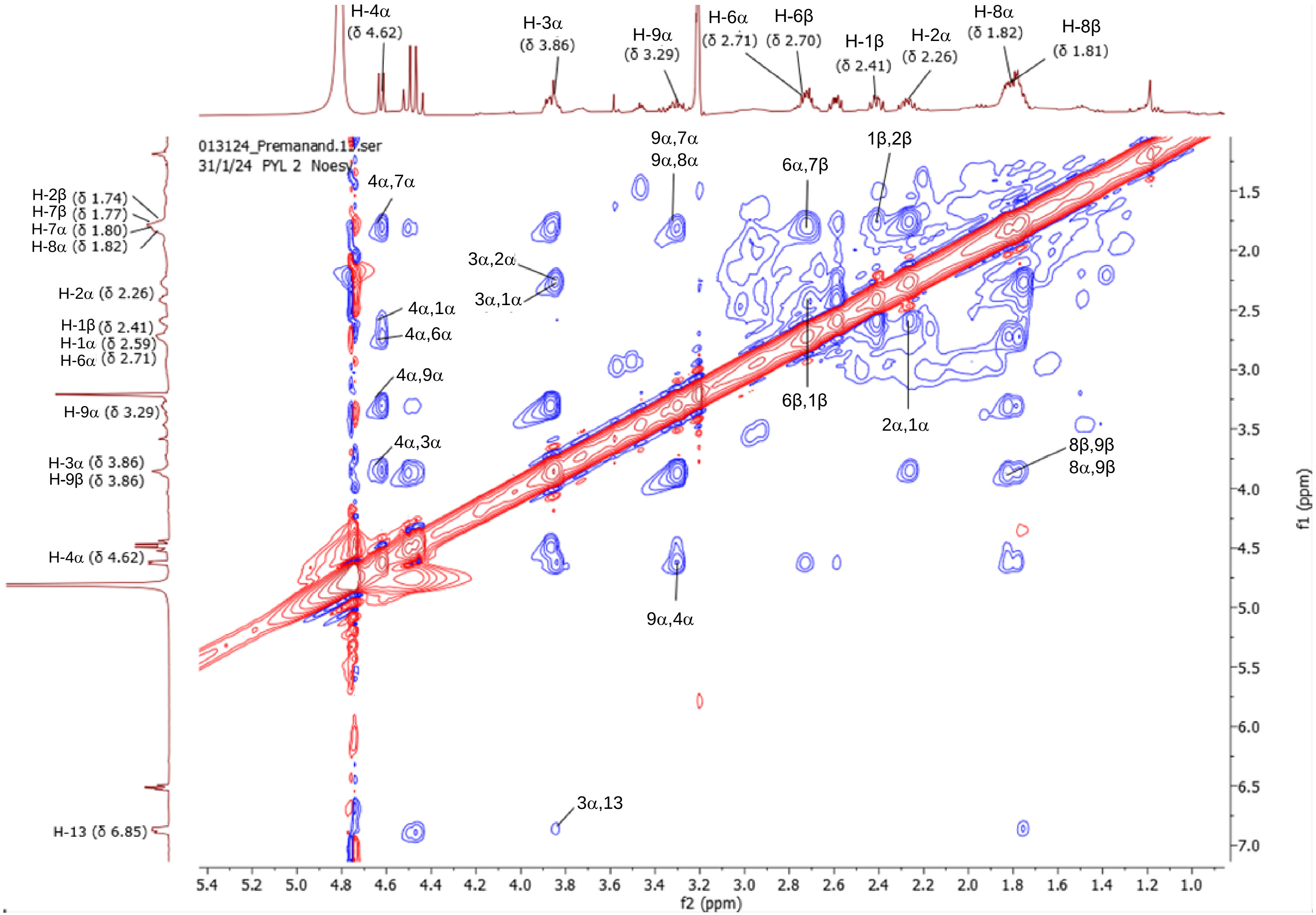

Figure S8. NOESY Spectrum of Pandazepine A (**1**) (CD_3OD, 600 MHz)

Sample Name:
Description:
Ionization Mode:ESI+
History:Determine m/z[Peak Detect[Centroid,30,Area];Correct Base[0.5%]];Correct Ba...

Operator:AccuTOF
Mass Calibration data:xCalib
Created:5/24/2024 10:18:17 AM
Created by:AccuTOF

Charge number:1
Tolerance:20.00(ppm), 0.00 .. 30.00(mmu)
Unsaturation Number:0.0 .. 25.0 (Fractio...
Element:^{12}C:5 .. 30, ^{1}H:10 .. 40, ^{14}N:0 .. 5, ^{16}O:0 .. 10

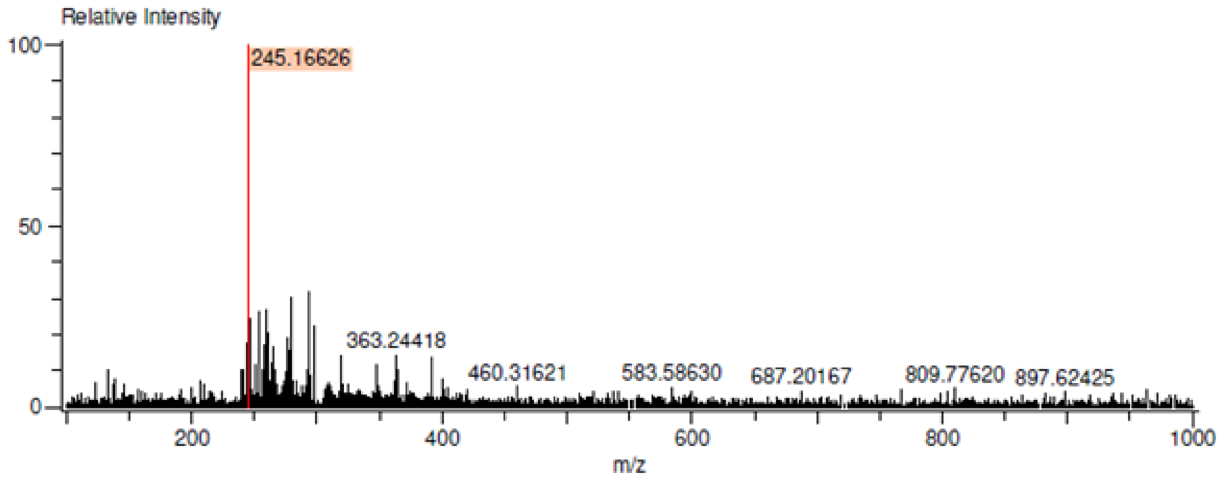

Mass	Intensity	Calc. Mass	Mass Difference (mmu)	Mass Difference (ppm)	Possible Formula
245.16626	15484.00	245.16539	0.88	3.57	$^{12}C_{15}{}^{1}H_{21}{}^{14}N_{2}{}^{16}O_{1}$
		245.16405	2.22	9.05	$^{12}C_{13}{}^{1}H_{19}{}^{14}N_{5}$
		245.16271	3.56	14.51	$^{12}C_{12}{}^{1}H_{23}{}^{14}N_{1}{}^{16}O_{4}$
		245.16136	4.90	19.98	$^{12}C_{10}{}^{1}H_{21}{}^{14}N_{4}{}^{16}O_{3}$

Figure S9. HRDARTMS of Pandazepine A (**1**)

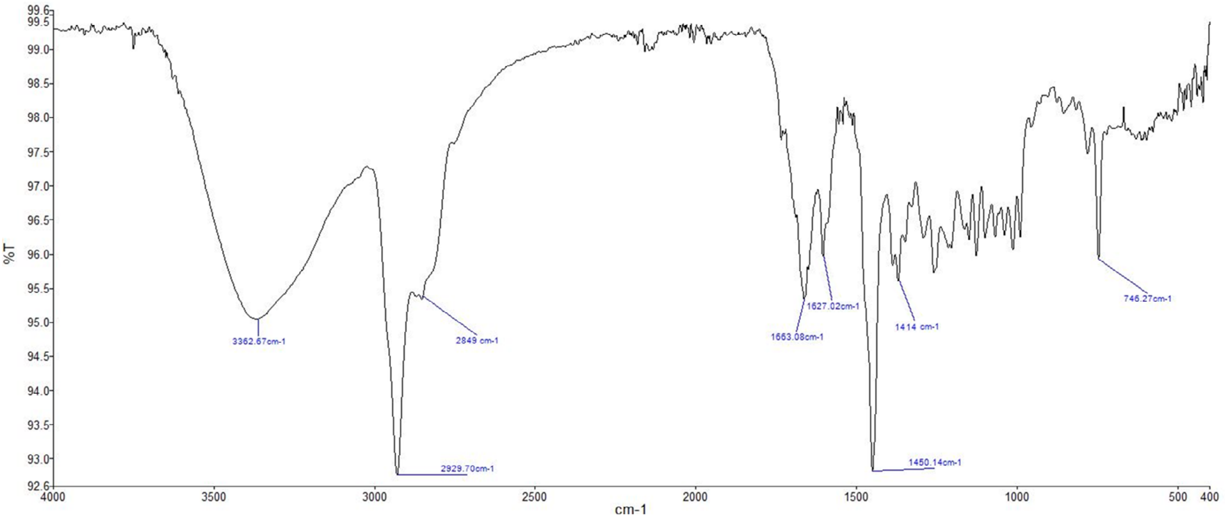

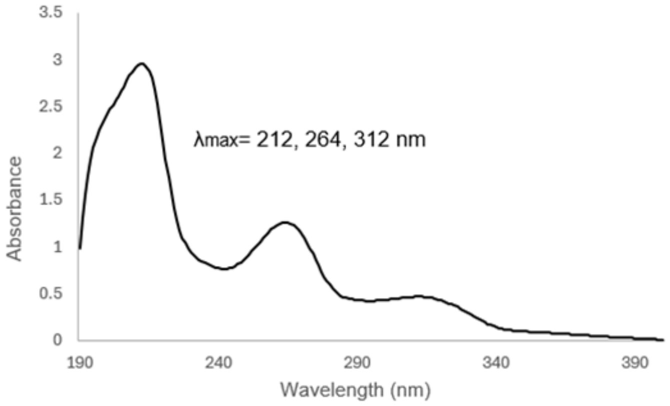

Figure S10. IR and UV spectra of Pandazepine A (**1**)

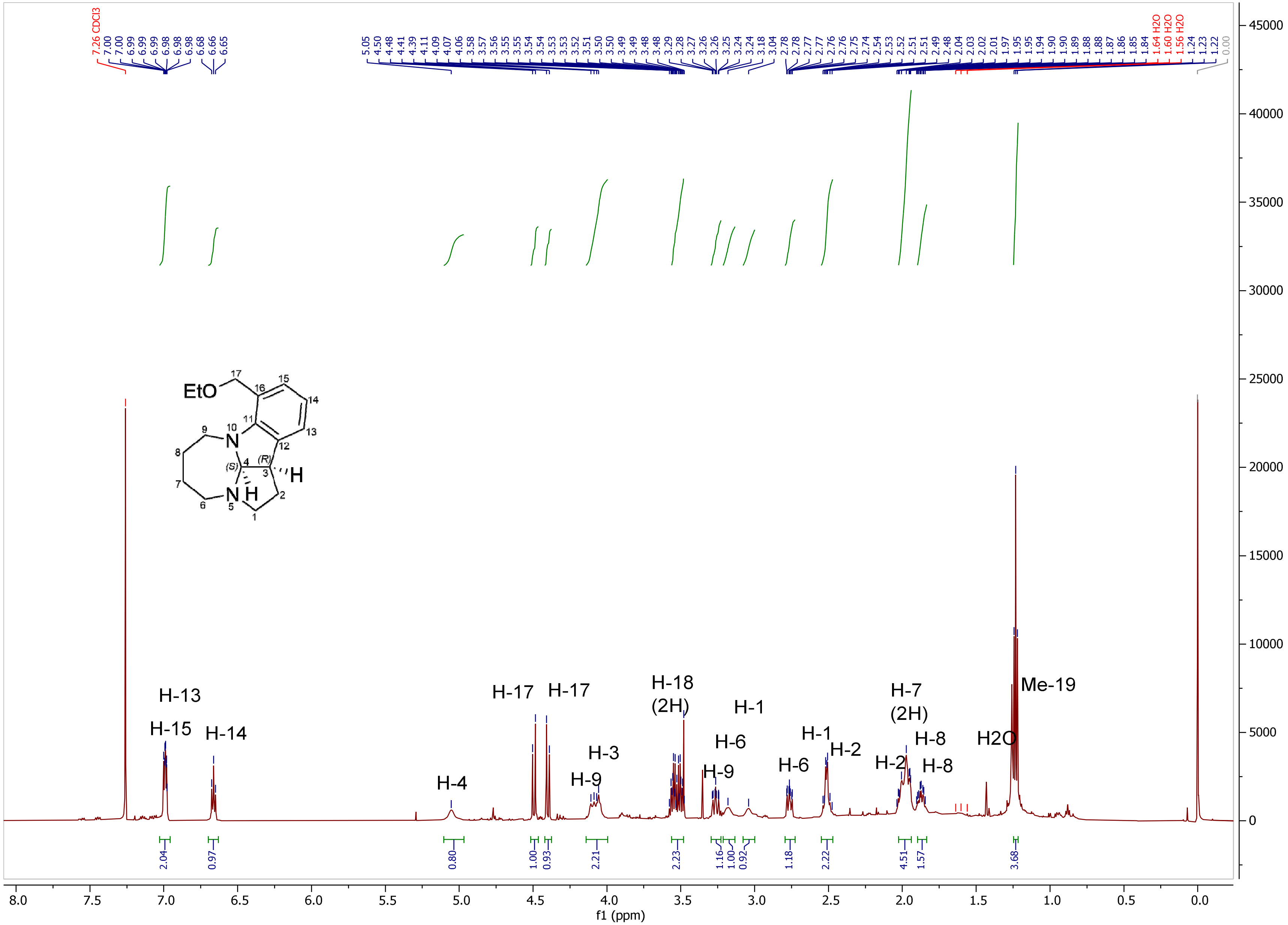

Figure S11. ^{1}H NMR Spectrum of Pandazepine B (**2**) ($CDCl_3$, 600 MHz)

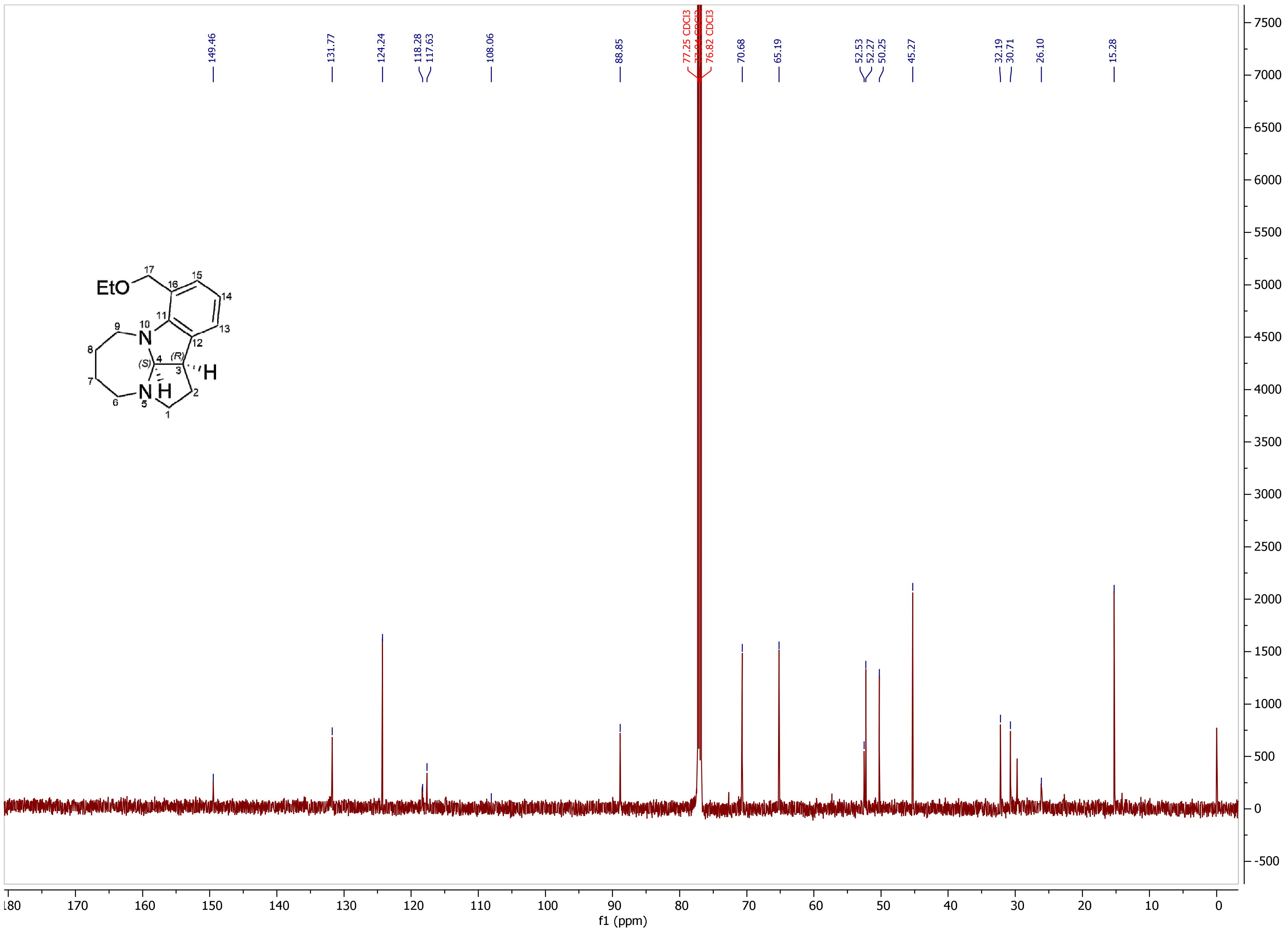

Figure S12. ^{13}C NMR Spectrum of Pandazepine B (**2**) ($CDCl_3$, 150 MHz)

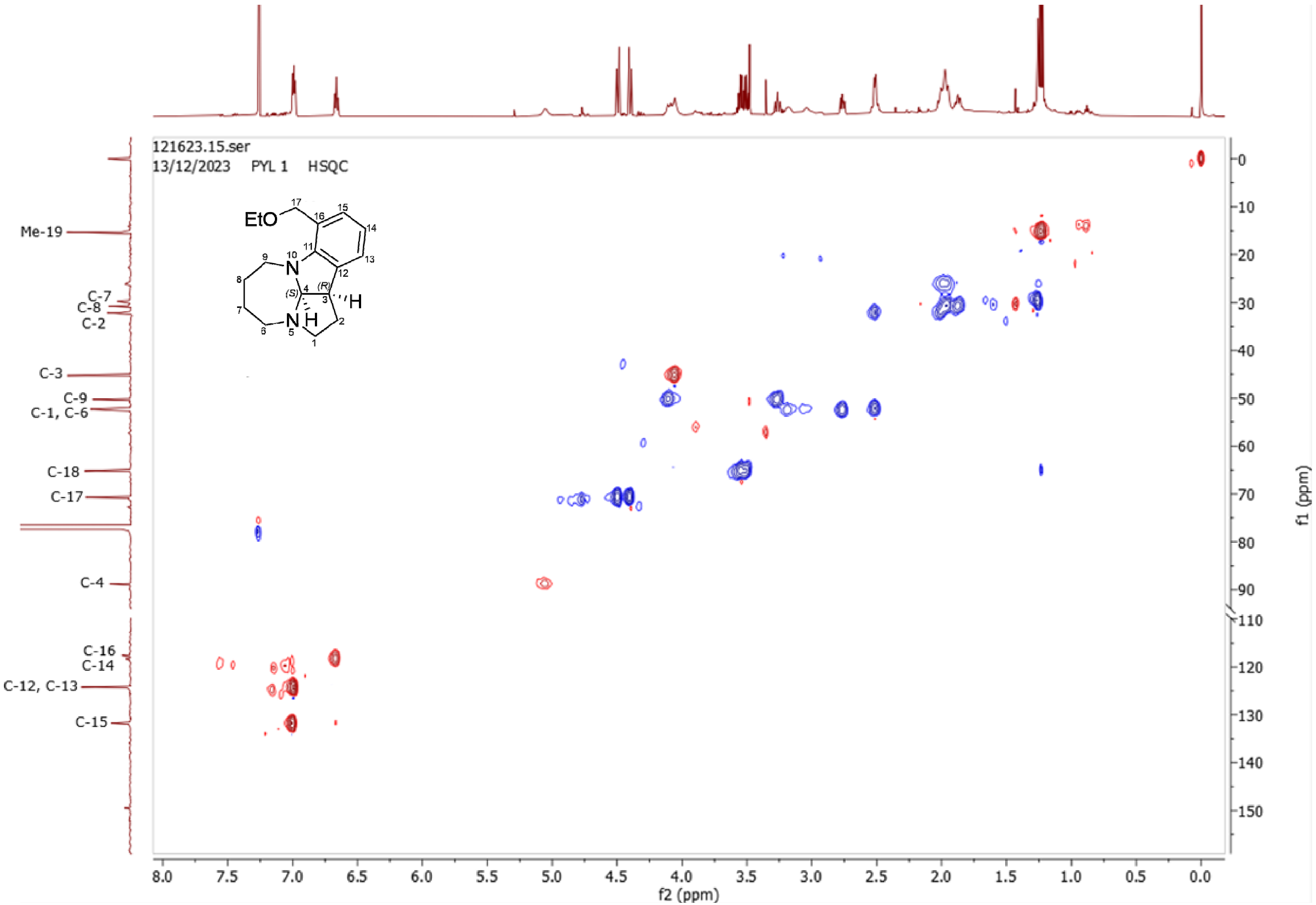

Figure S13. HSQC Spectrum of Pandazepine B (**2**) ($CDCl_3$, 600 MHz)

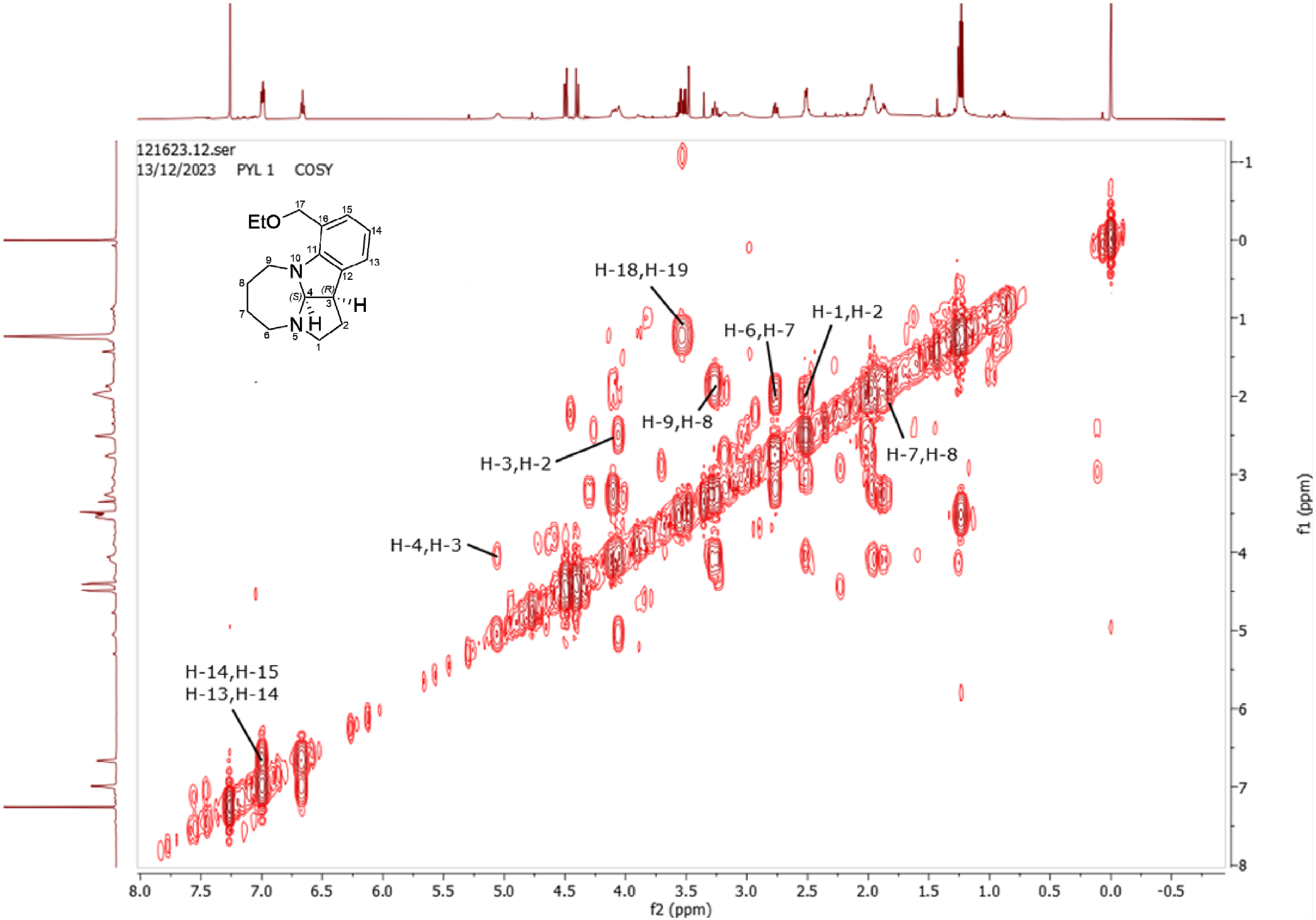

Figure S14. COSY Spectrum of Pandazepine B (**2**) ($CDCl_3$, 600 MHz)

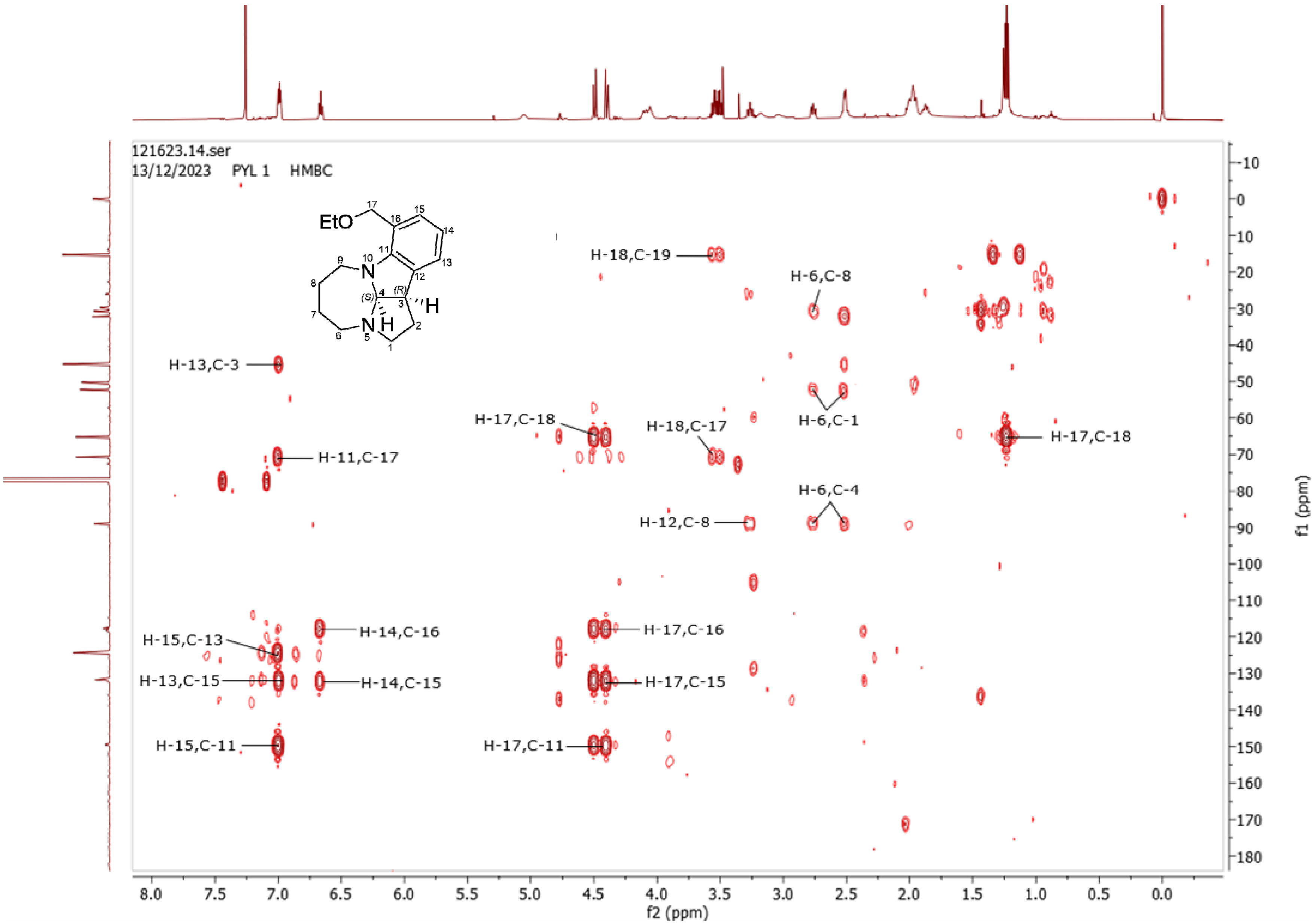

Figure S15. HMBC Spectrum of Pandazepine B (**2**) ($CDCl_3$, 600 MHz)

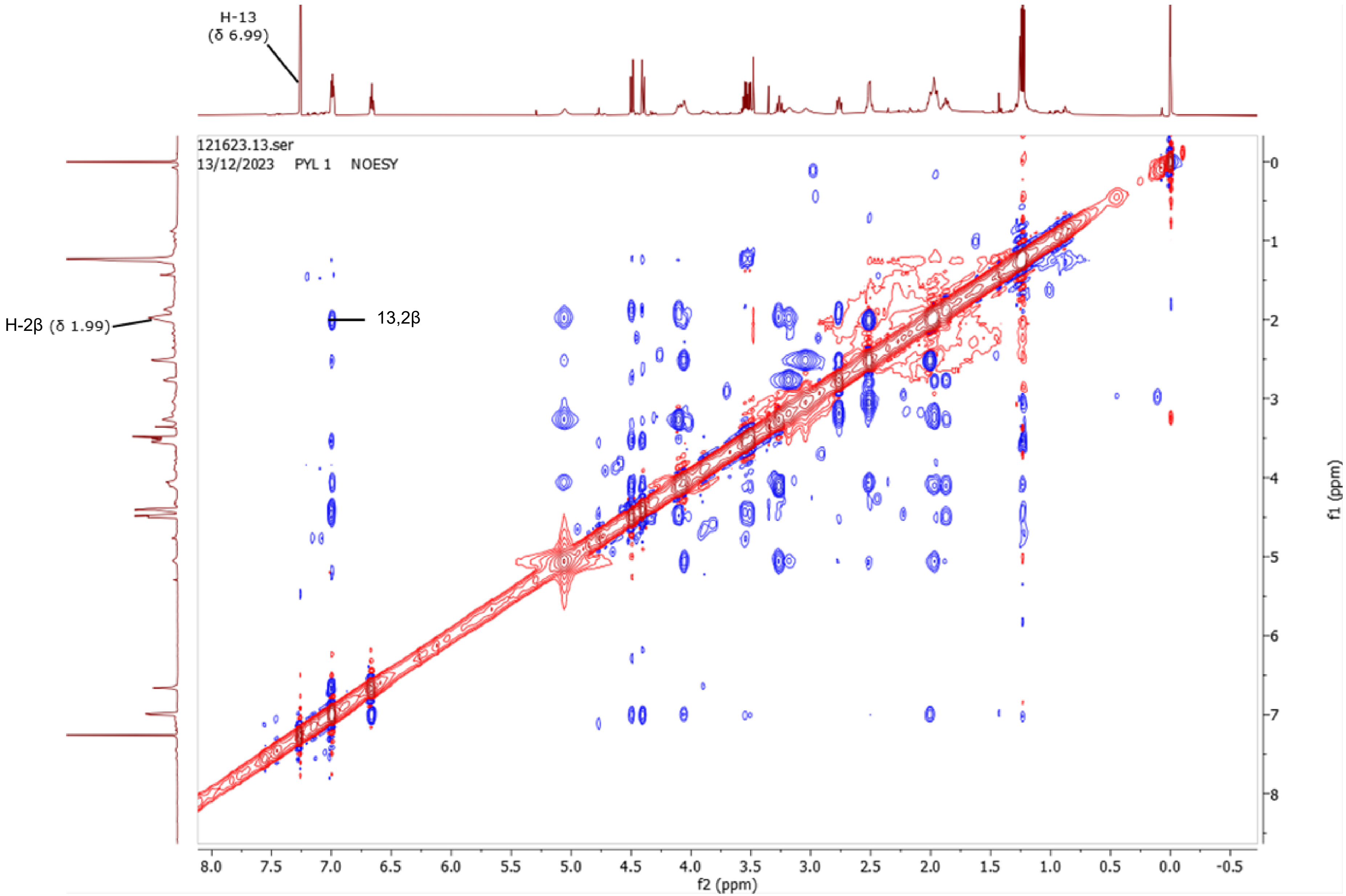

Figure S16. NOESY Spectrum of Pandazepine B (**2**) ($CDCl_3$, 600 MHz)

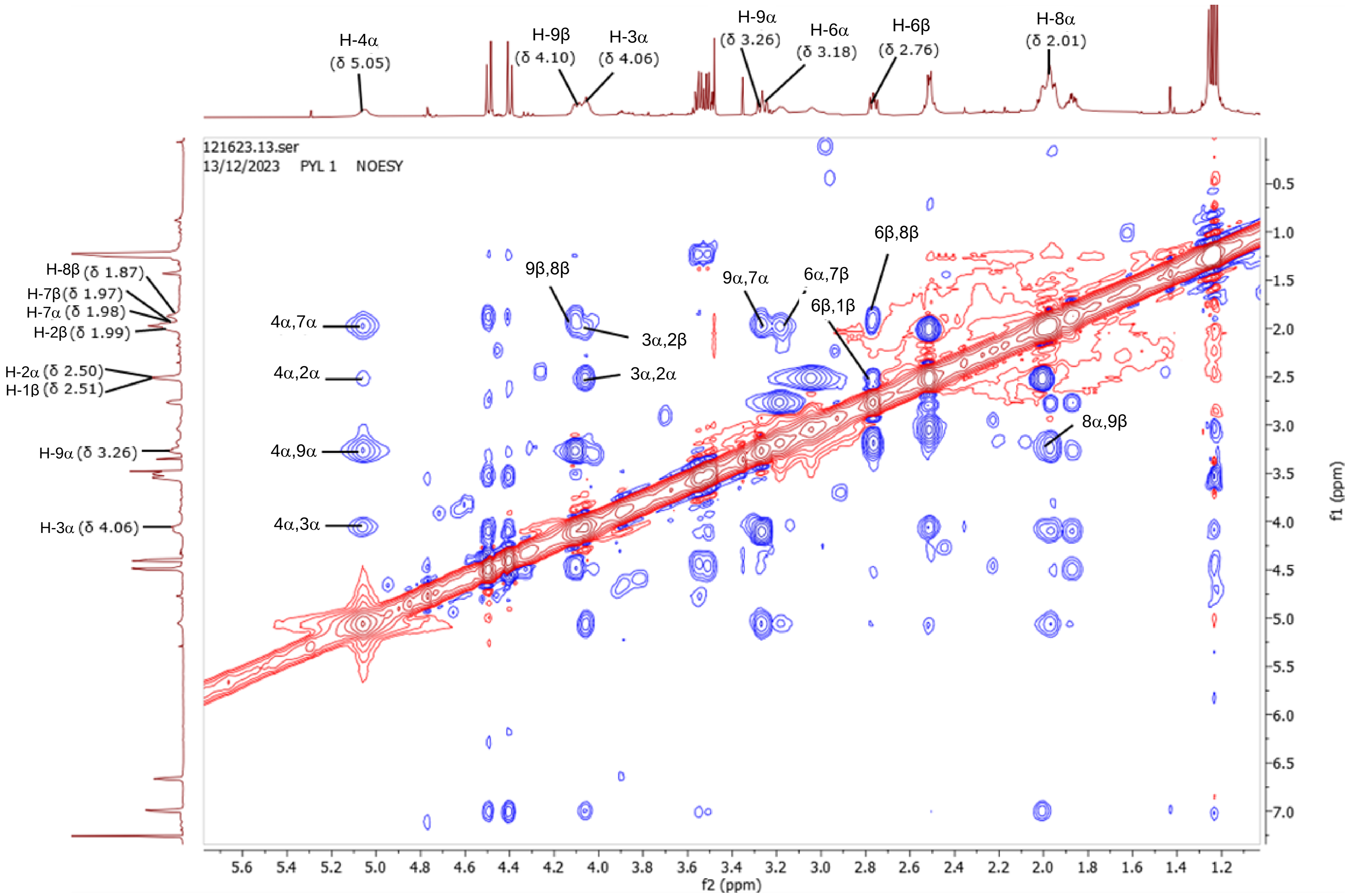

Figure S17. NOESY Spectrum of Pandazepine B (**2**) ($CDCl_3$, 600 MHz)

Sample Name:
Description:
Ionization Mode:ESI+
History:Determine m/z[Peak Detect[Centroid,30,Area];Correct Base[0.5%]];Correct Ba...

Operator:AccuTOF
Mass Calibration data:xCalib
Created:2/14/2024 2:47:04 PM
Created by:AccuTOF

Charge number:1
Tolerance:20.00(ppm), 0.00 .. 30.00(mmu)
Unsaturation Number:0.0 .. 25.0 (Fractio...
Element:^{12}C:0 .. 50, ^{1}H:0 .. 35, ^{14}N:0 .. 5, ^{16}O:0 .. 5

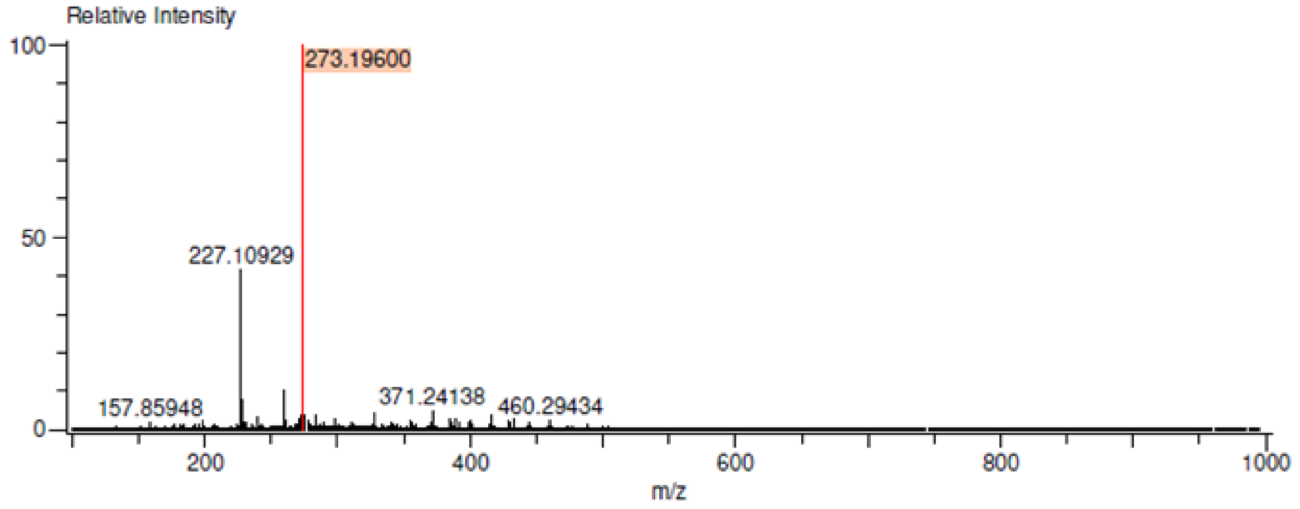

Mass	Intensity	Calc. Mass	Mass Difference (mmu)	Mass Difference (ppm)	Possible Formula
273.19600	263339.00	273.19535	0.65	2.39	$^{12}C_{15}{}^{1}H_{23}{}^{14}N_{5}$
		273.19669	-0.69	-2.53	$^{12}C_{17}{}^{1}H_{25}{}^{14}N_{2}{}^{16}O_{1}$
		273.19401	1.99	7.29	$^{12}C_{14}{}^{1}H_{27}{}^{14}N_{1}{}^{16}O_{4}$
		273.19266	3.33	12.20	$^{12}C_{12}{}^{1}H_{25}{}^{14}N_{4}{}^{16}O_{3}$

Figure S18. HRDARTMS of Pandazepine B (**2**)

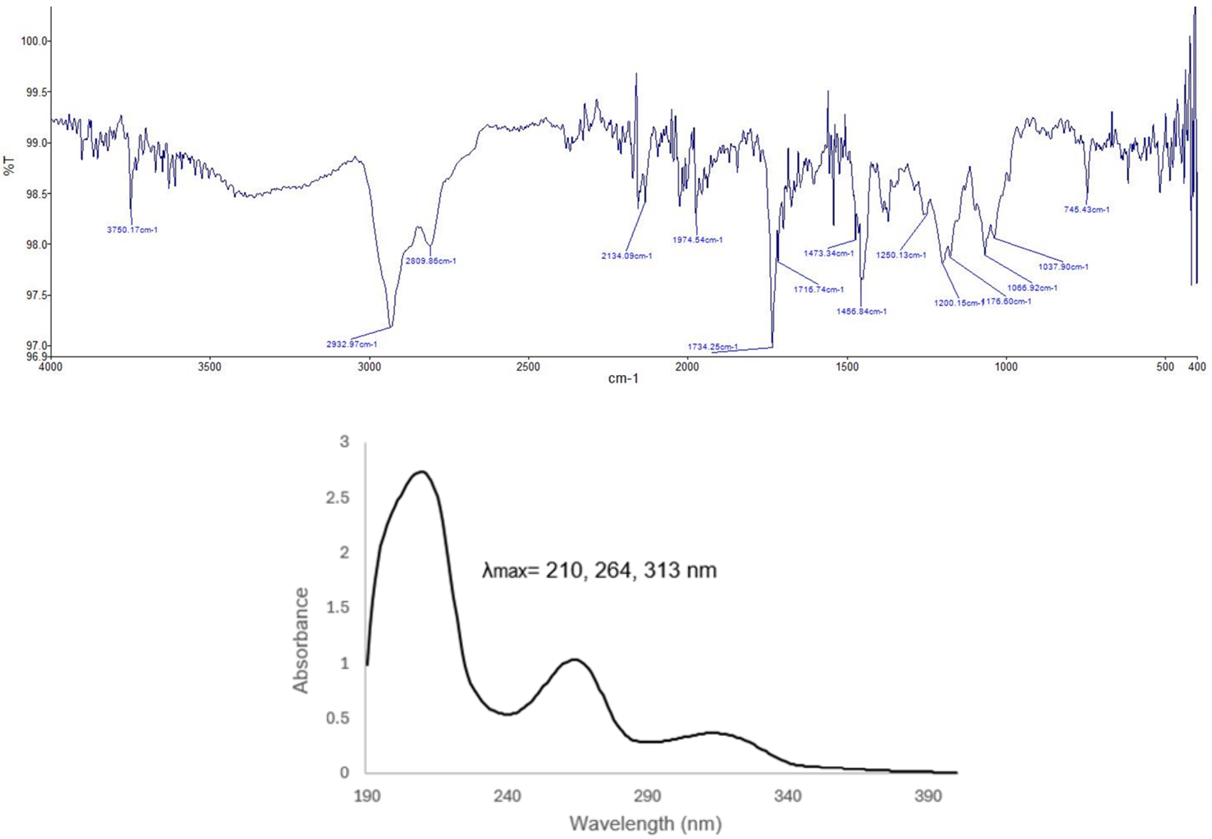

Figure S19. IR and UV Spectra of Pandazepine B (**2**)

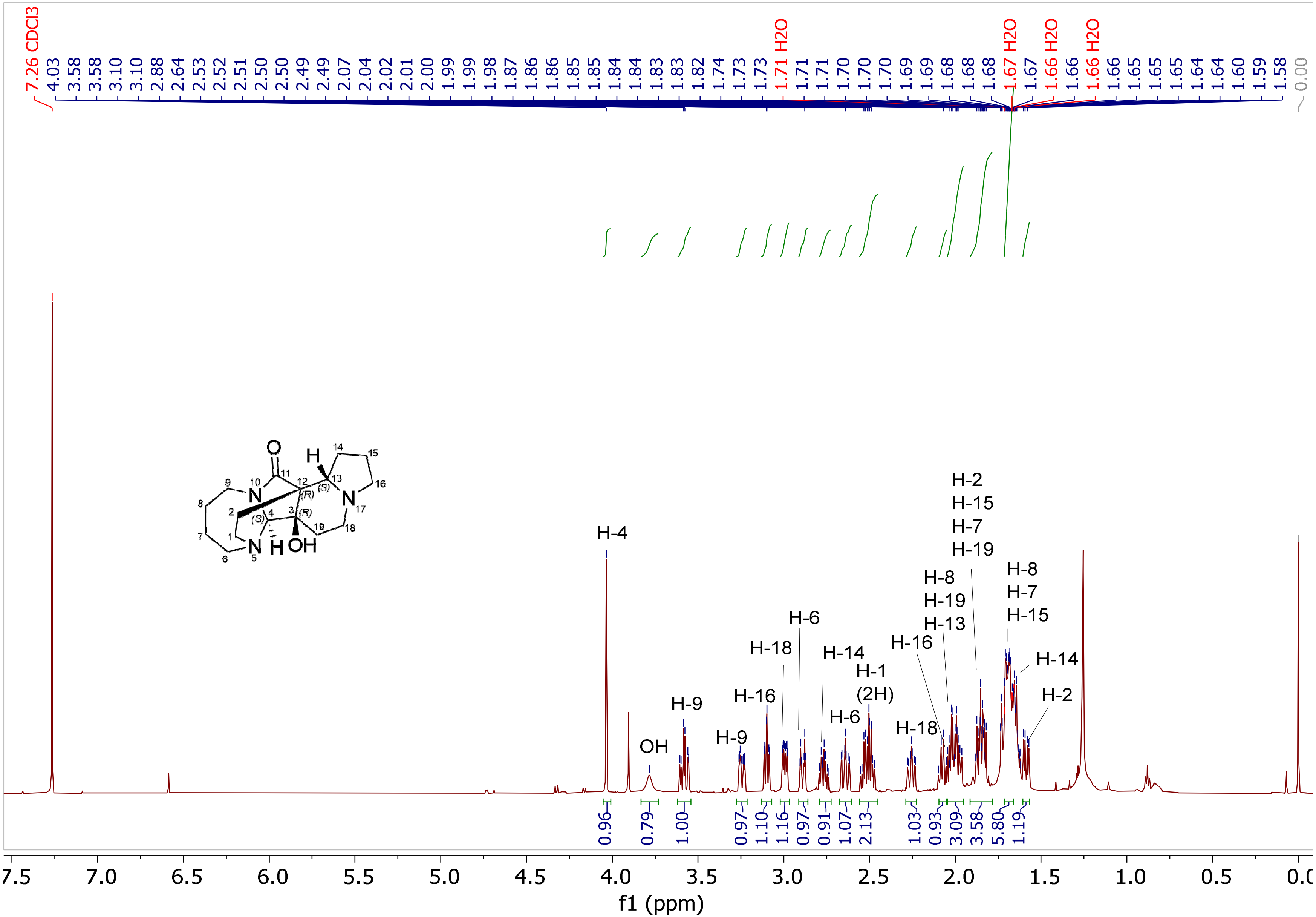

Figure S20. ^{1}H NMR Spectrum of Pandazepine C (**3**) ($CDCl_3$, 600 MHz)

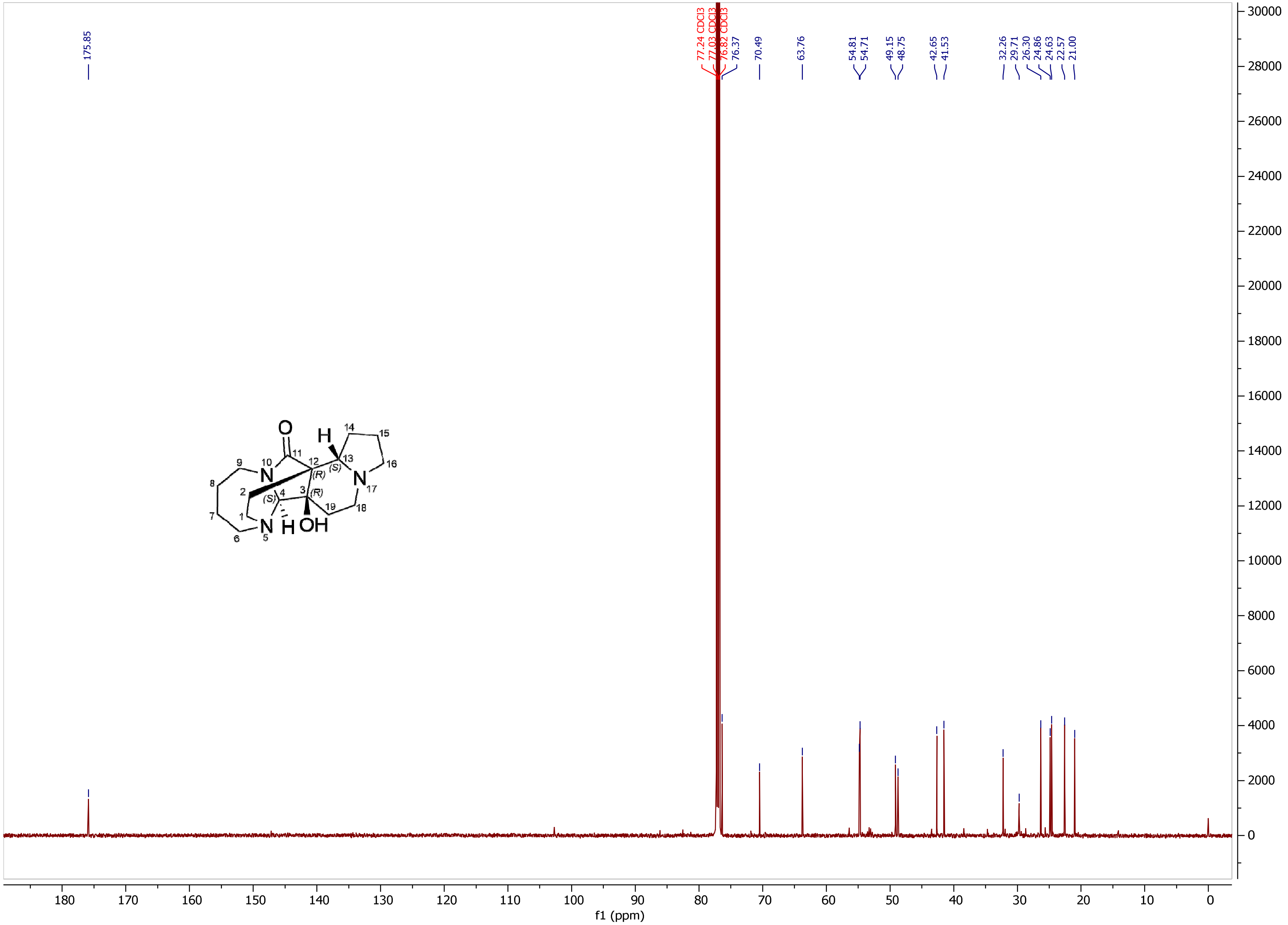

Figure S21. ^{13}C NMR Spectrum of Pandazepine C (**3**) ($CDCl_3$, 150 MHz)

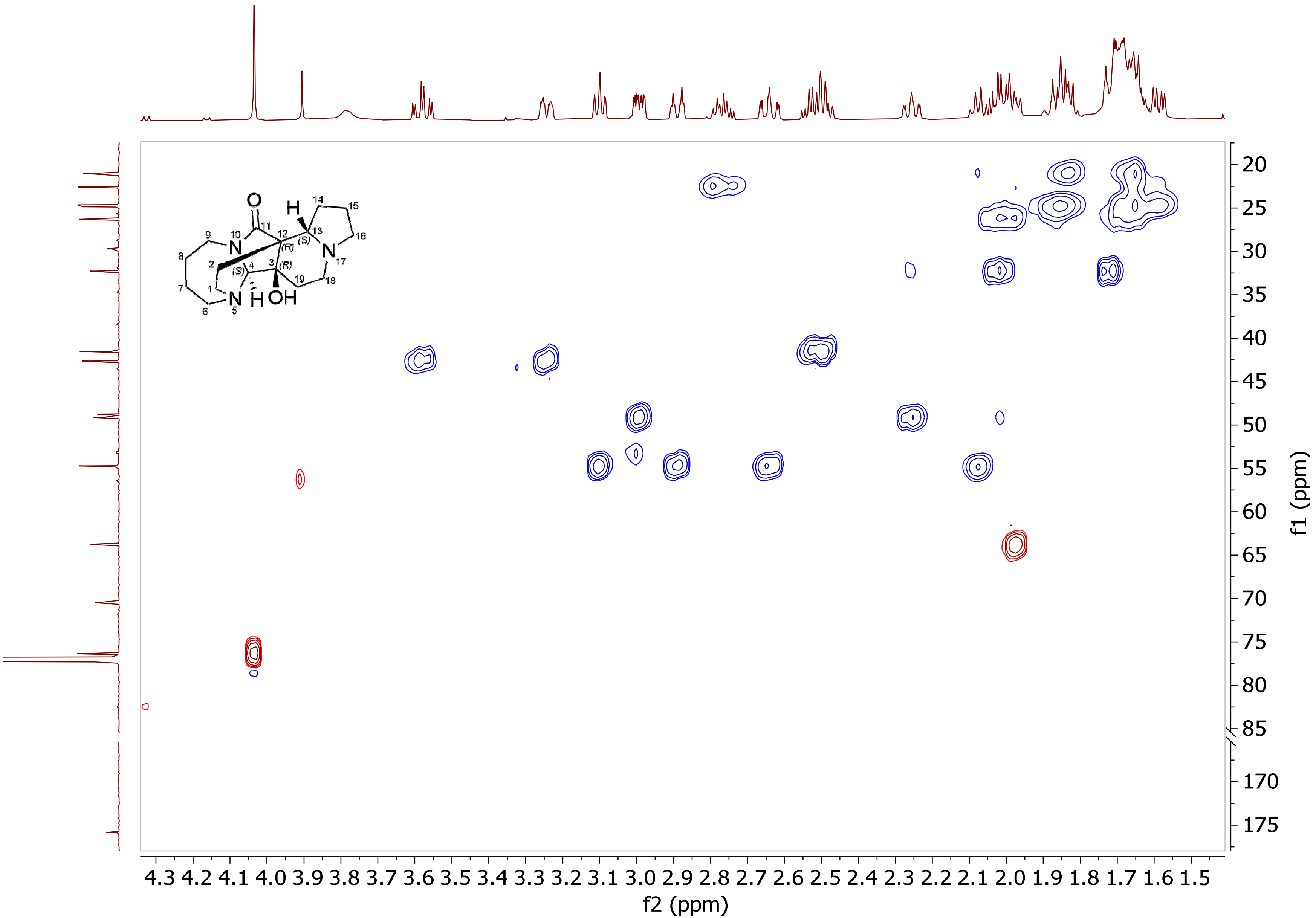

Figure S22. HSQC Spectrum of Pandazepine C (**3**) ($CDCl_3$, 600 MHz)

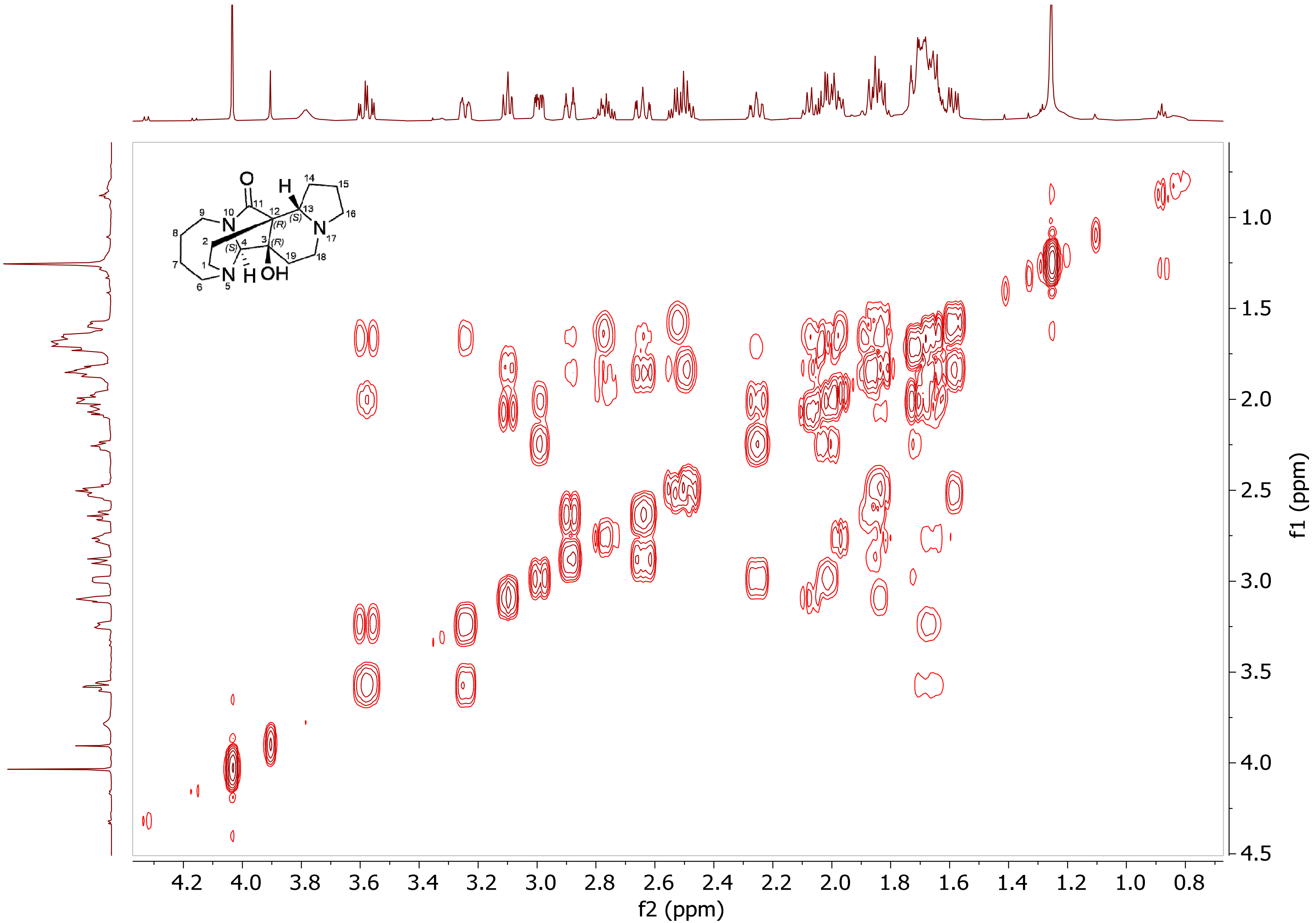

Figure S23. COSY Spectrum of Pandazepine C (**3**) ($CDCl_3$, 600 MHz)

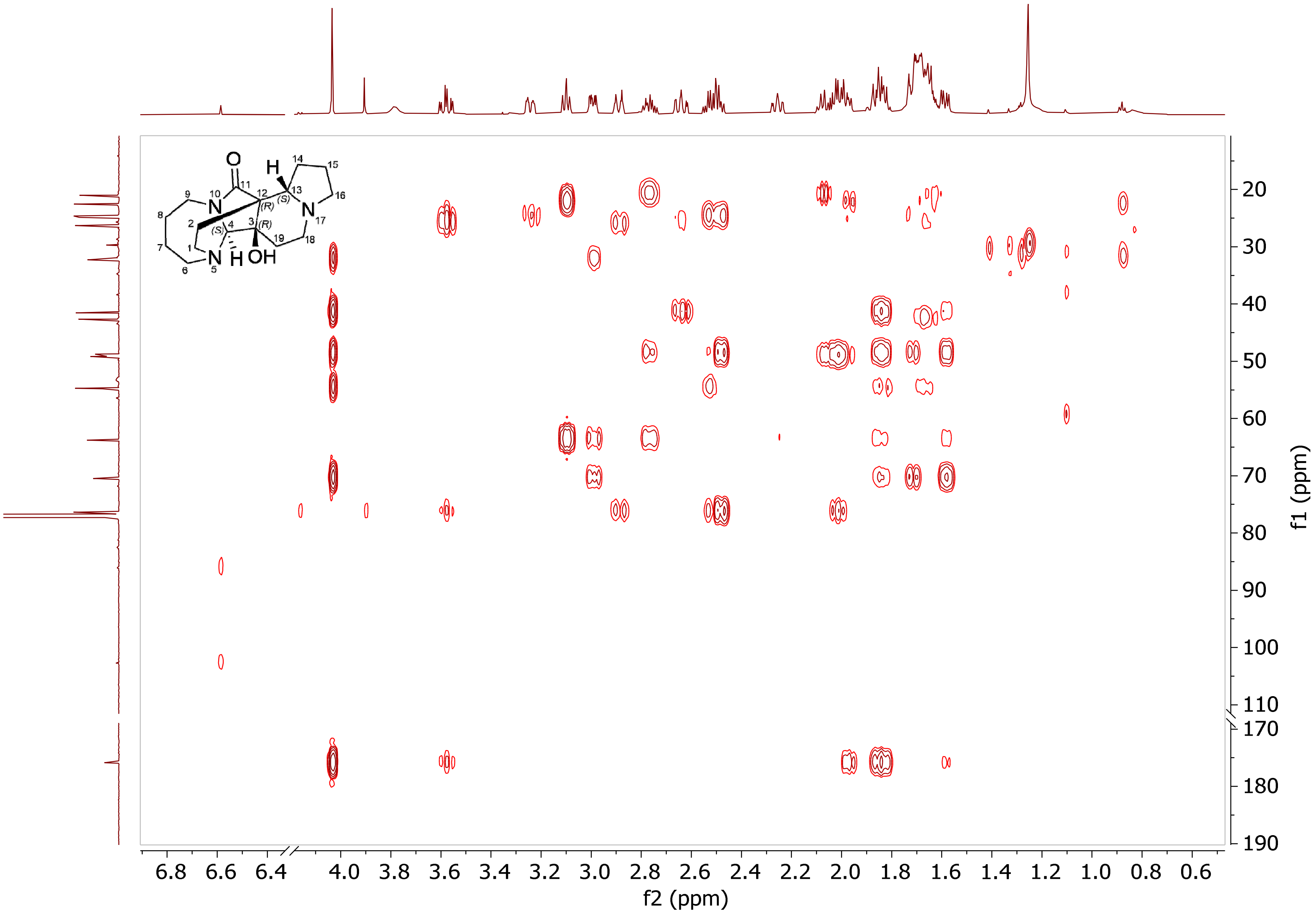

Figure S24. HMBC Spectrum of Pandazepine C (**3**) ($CDCl_3$, 600 MHz)

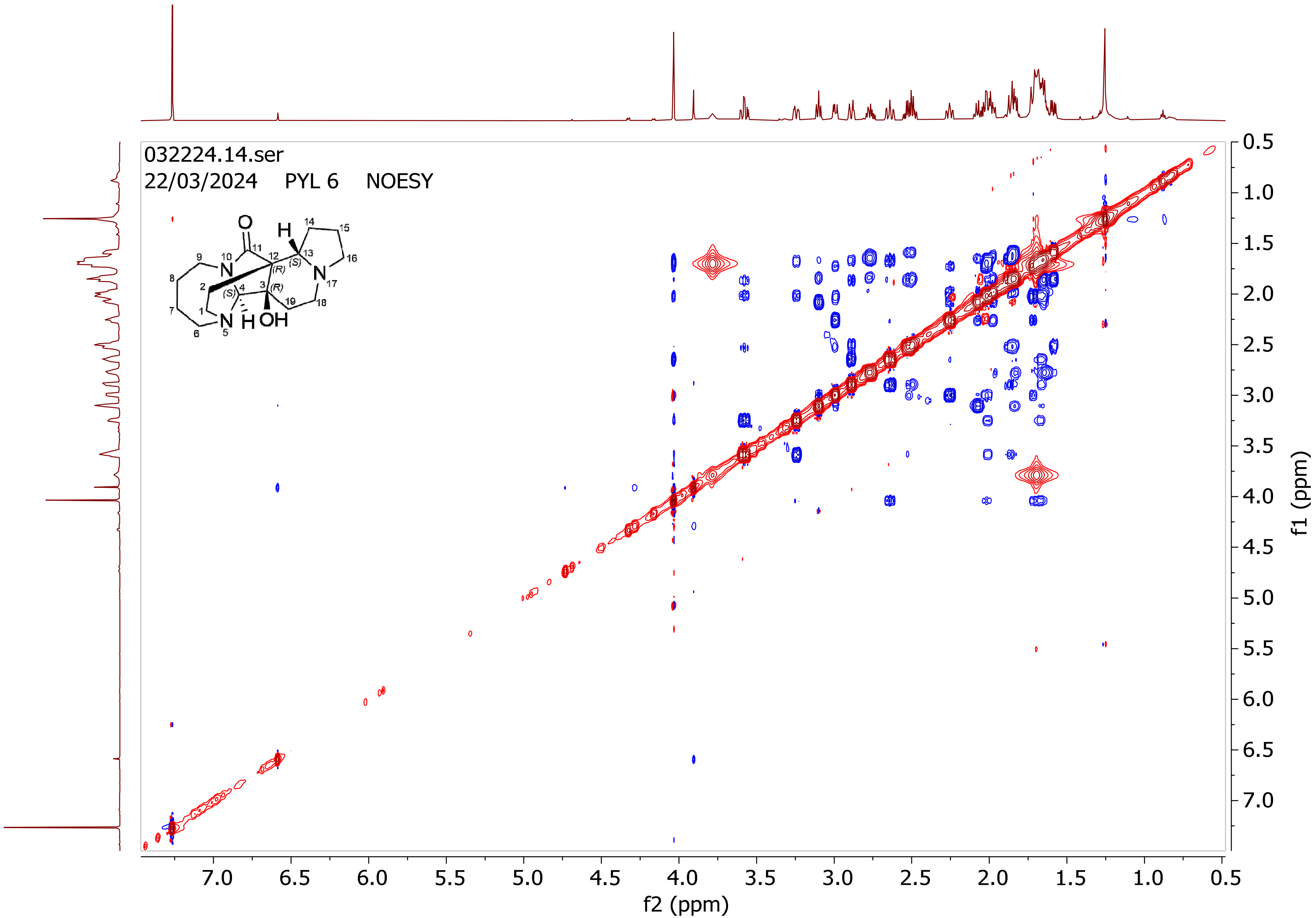

Figure S25. NOESY Spectrum of Pandazepine C (**3**) ($CDCl_3$, 600 MHz)

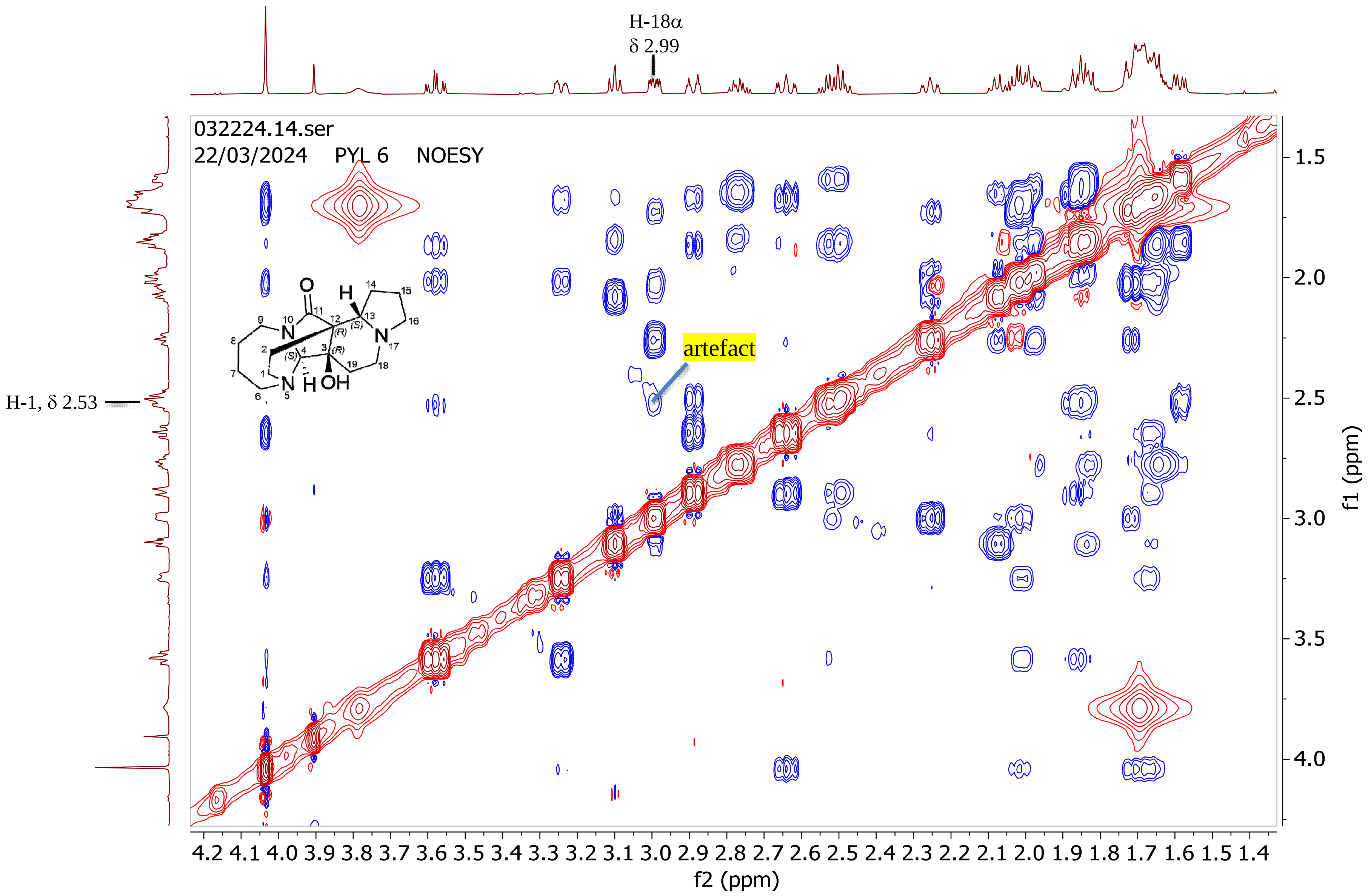

Figure S26. NOESY Spectrum of Pandazepine C (**3**) ($CDCl_3$, 600 MHz)

To clarify an ambiguous NOE correlation observed between H-1 (δ_H 2.53) and H-18α (δ_H 2.99), targeted 1D NOESY experiments were performed (**Fig. S28** and **S29**). Irradiation of H-18α (δ_H 2.99) led to the expected enhancement of H-18β (δ_H 2.26), H-16α (δ_H 3.10), and H-19 (δ_H 1.72 and 2.01). However, irradiation of H-1 (δ_H 2.53) enhanced H-6β (δ_H 2.89) and H-2 (δ_H 1.58 and 1.85) but showed no observable enhancement of H-18α. This lack of reciprocal enhancement proves the initial H-1/H-18α correlation was an artefact, thus confirming the proposed relative configuration of **3**.

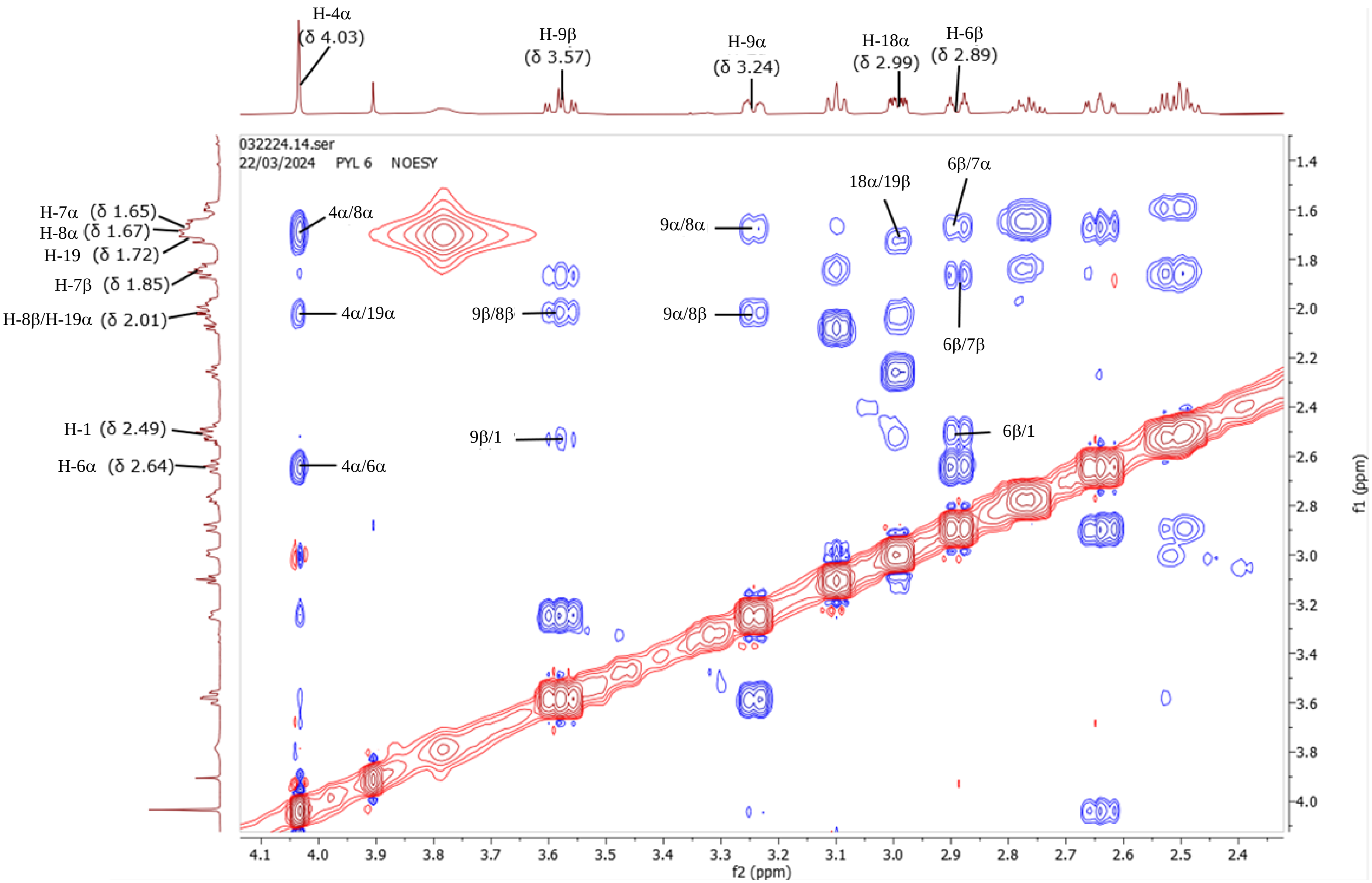

Figure S27. NOESY Spectrum of Pandazepine C (**3**) ($CDCl_3$, 600 MHz)

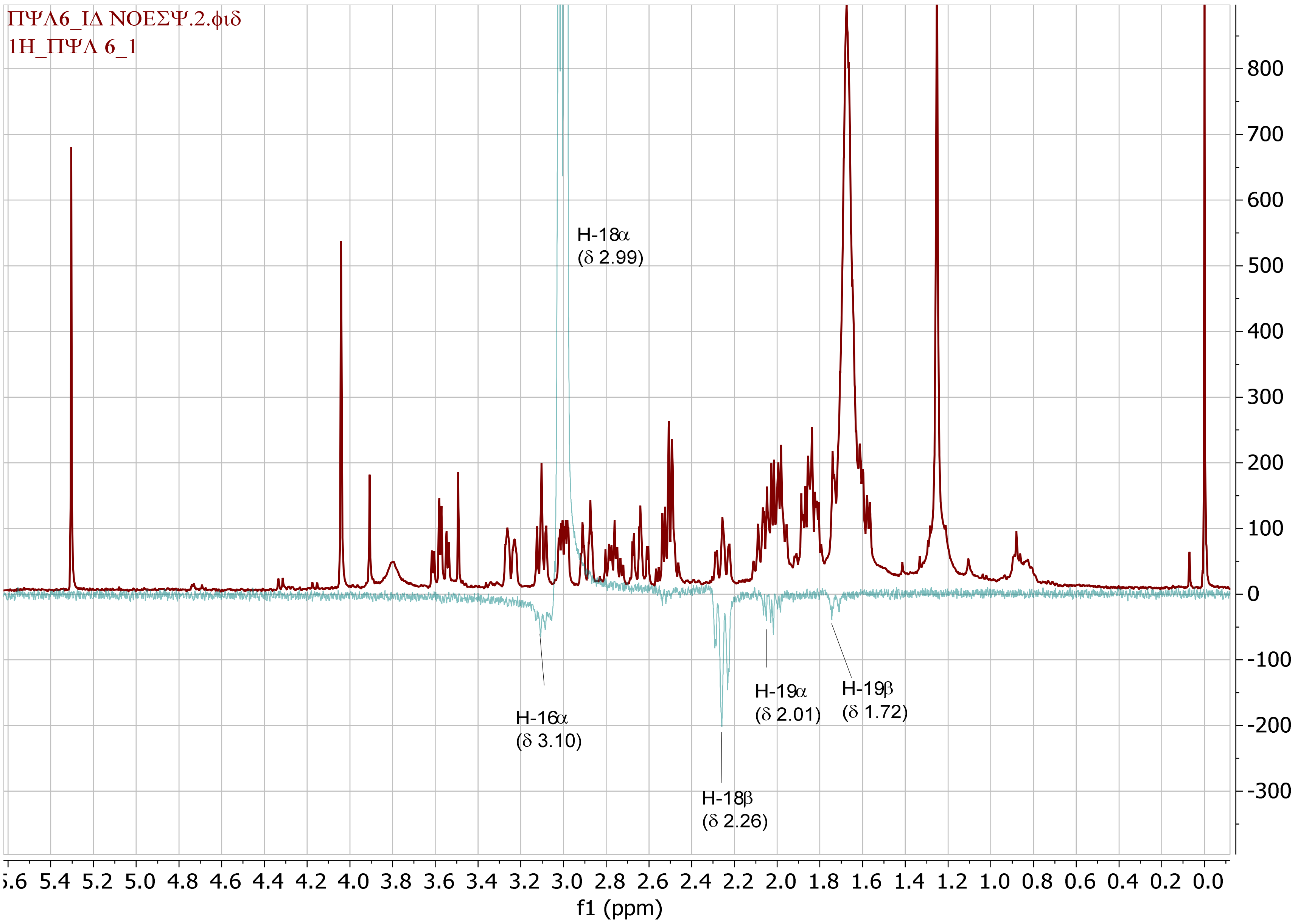

Figure S28. 1D NOESY Spectrum of Pandazepine C (**3**) ($CDCl_3$, 600 MHz)

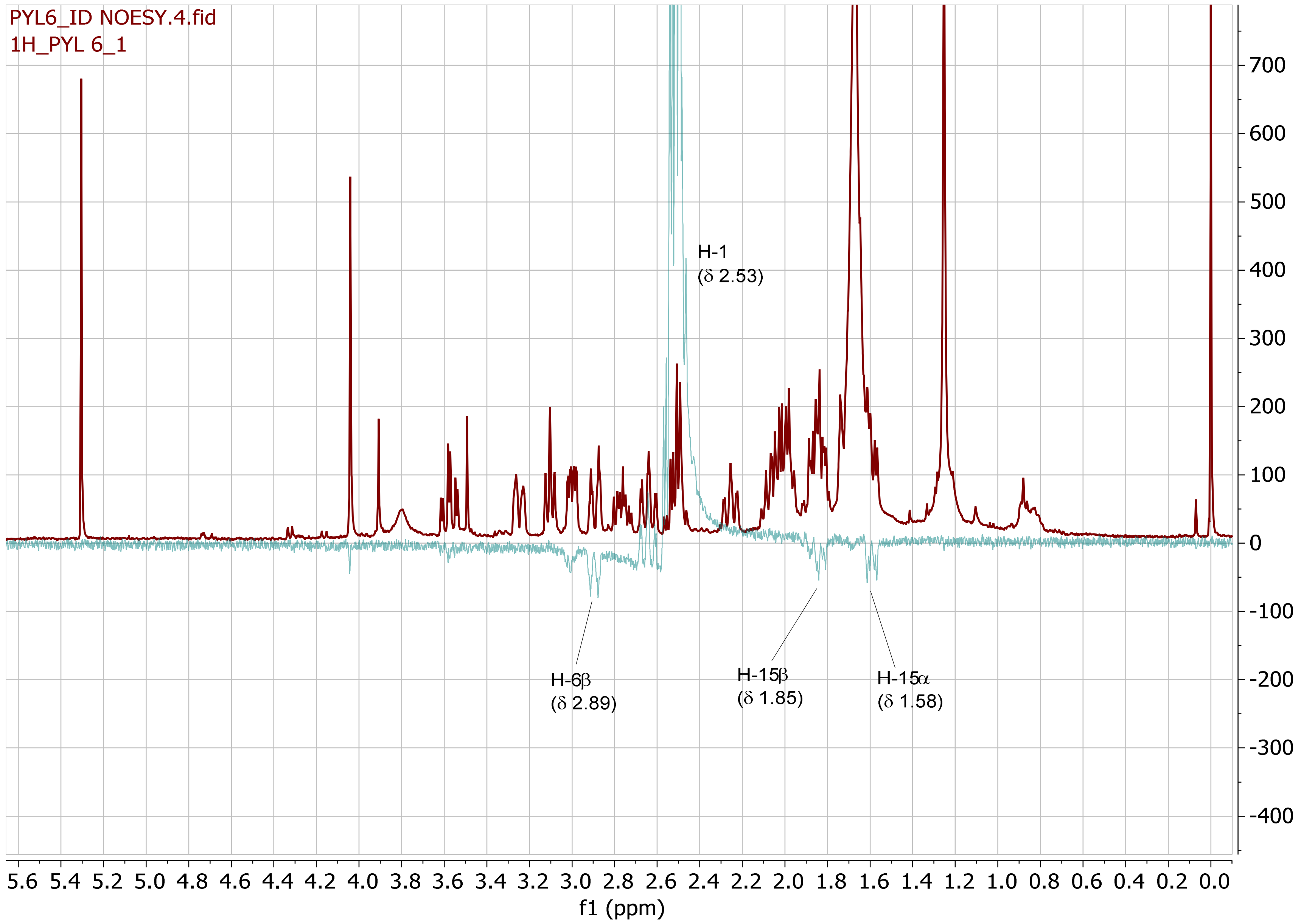

Figure S29. 1D NOESY Spectrum of Pandazepine C (**3**) ($CDCl_3$, 600 MHz)

Sample Name:
Description:
Ionization Mode:ESI+
History:Determine m/z[Peak Detect[Centroid,30,Area];Correct Base[0.5%]];Correct Ba...

Operator:AccuTOF
Mass Calibration data:iCalib
Created:10/23/2024 1:31:50 PM
Created by:AccuTOF

Charge number:1
Element:^{12}C:10 .. 30, ^{1}H:10 .. 50, ^{14}N:0 .. 5, ^{16}O:0 .. 5

Tolerance:20.00(ppm), 0.00 .. 30.00(mmu)

Unsaturation Number:0.0 .. 25.0 (Fractio...

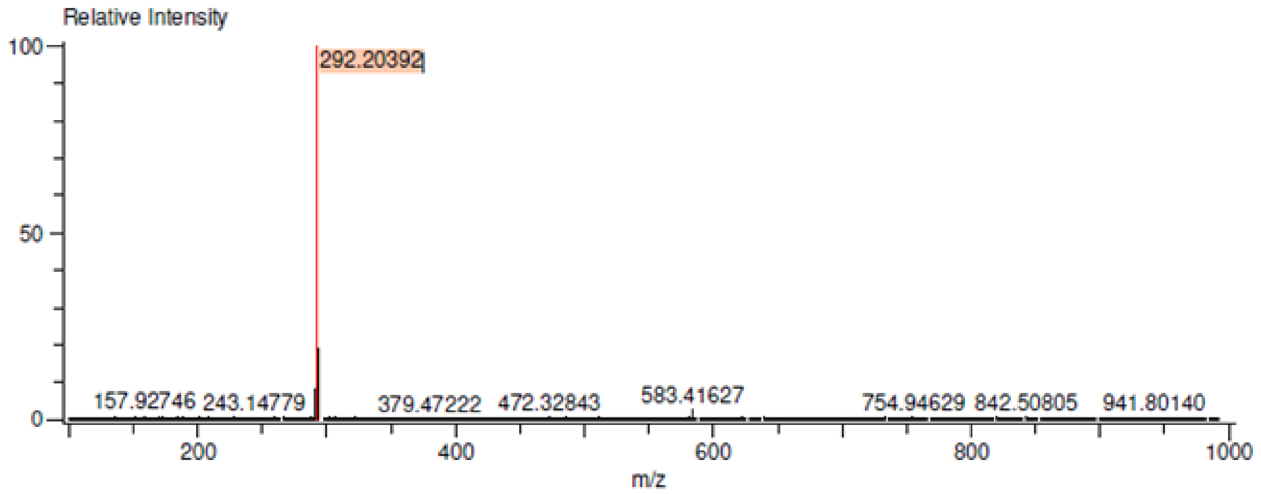

Mass	Intensity	Calc. Mass	Mass Difference (mmu)	Mass Difference (ppm)	Possible Formula
292.20392	171683.25	292.20384	0.07	0.25	$^{12}C_{18}{}^{1}H_{28}{}^{16}O_{3}$
		292.20250	1.42	4.85	$^{12}C_{16}{}^{1}H_{26}{}^{14}N_{3}{}^{16}O_{2}$
		292.20652	-2.61	-8.92	$^{12}C_{21}{}^{1}H_{26}{}^{14}N_{1}$
		292.19982	4.10	14.02	$^{12}C_{13}{}^{1}H_{28}{}^{14}N_{2}{}^{16}O_{5}$
		292.19848	5.44	18.61	$^{12}C_{11}{}^{1}H_{26}{}^{14}N_{5}{}^{16}O_{4}$

Figure S30. HRDARTMS of Pandazepine C (**3**)

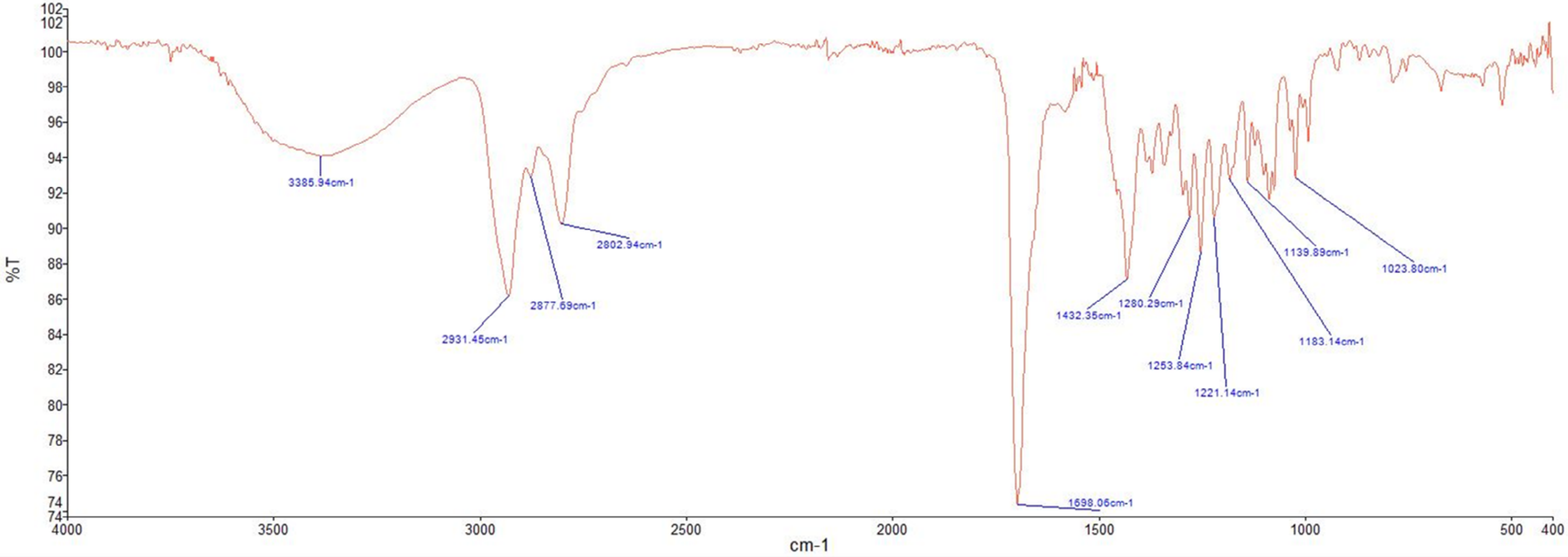

Figure S31. IR Spectrum of Pandazepine C (**3**)

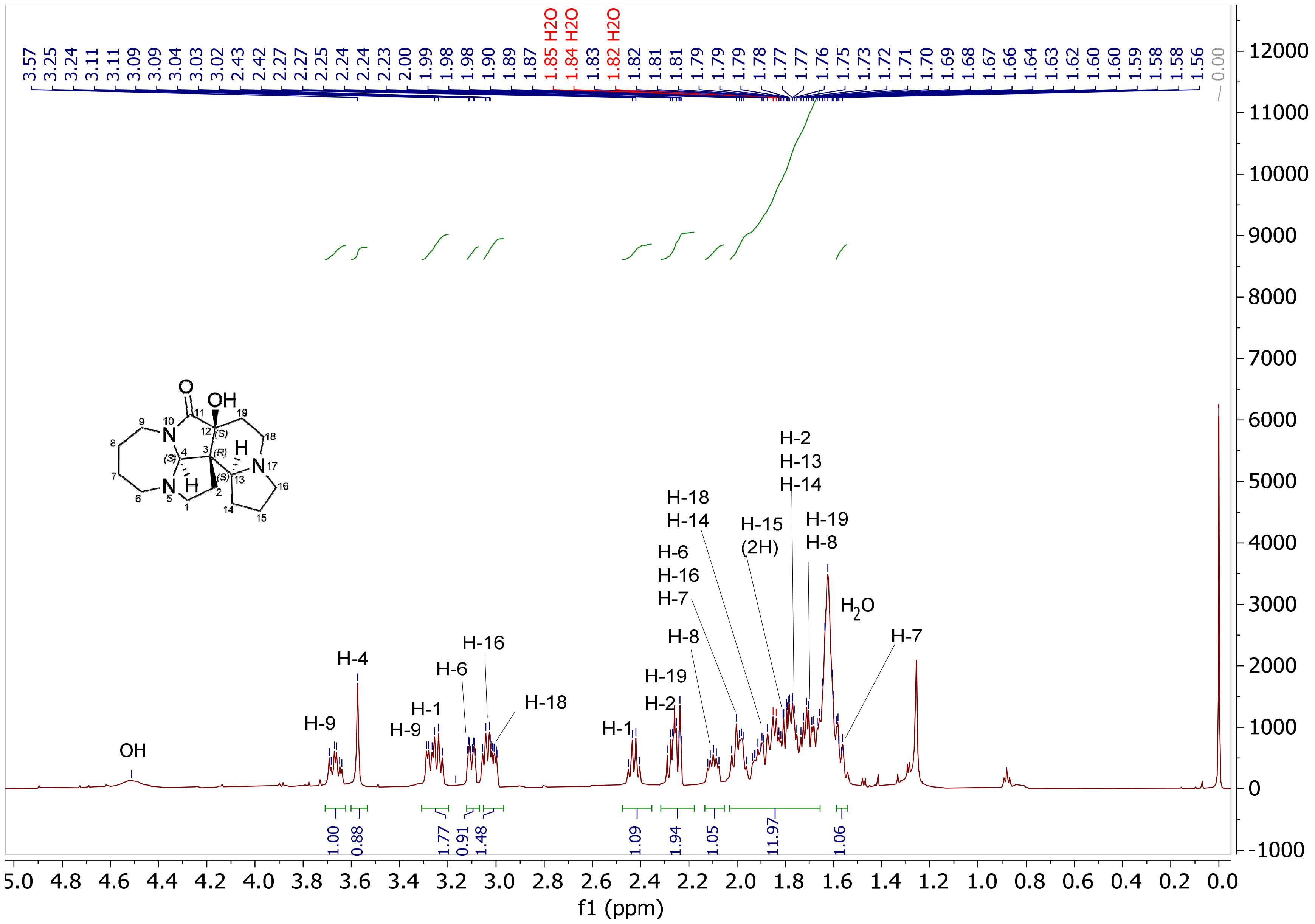

Figure S32. ^{1}H NMR Spectrum of Pandazepine D (**4**) ($CDCl_3$, 600 MHz)

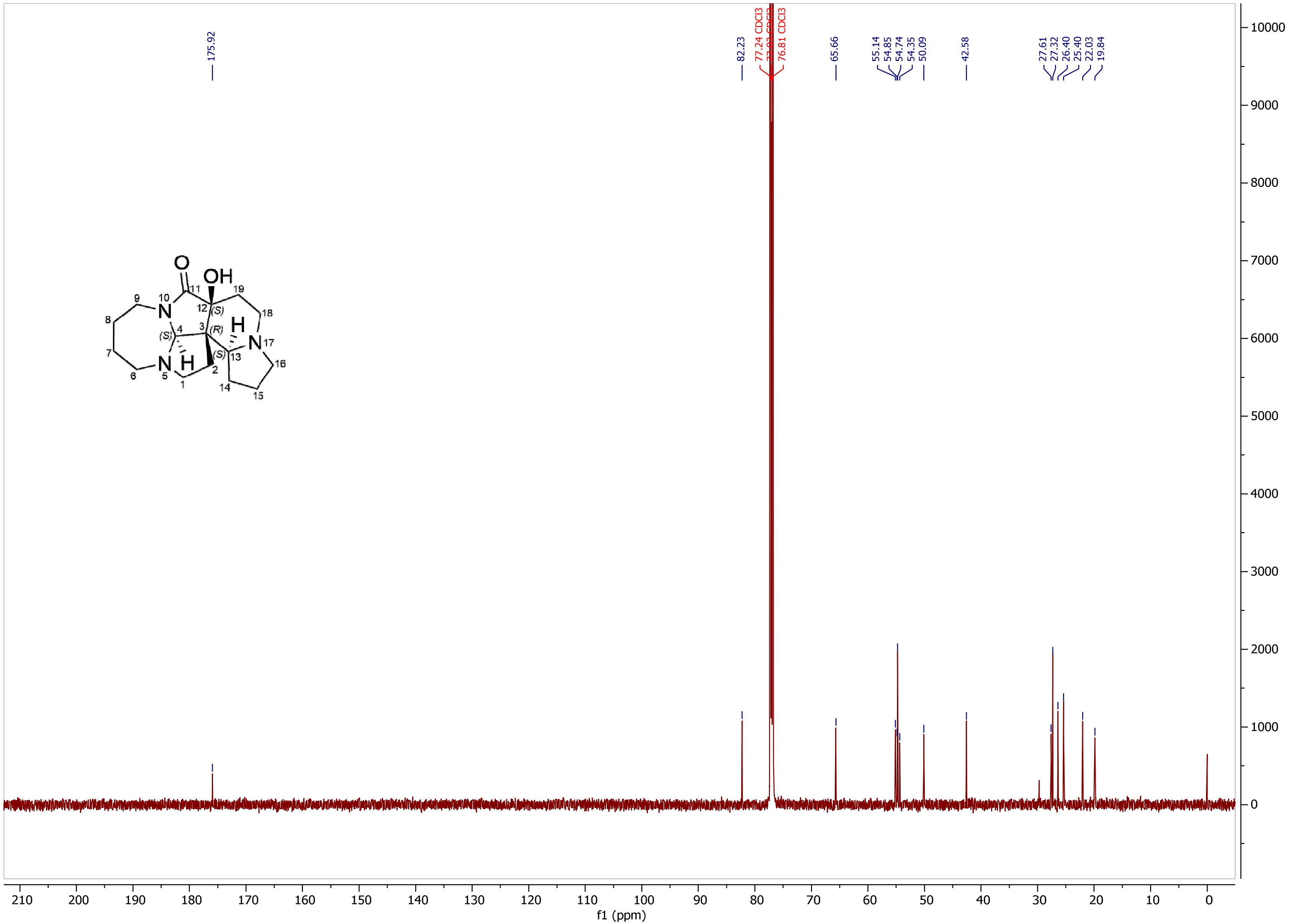

Figure S33. ^{13}C NMR Spectrum of Pandazepine D (**4**) ($CDCl_3$, 150 MHz)

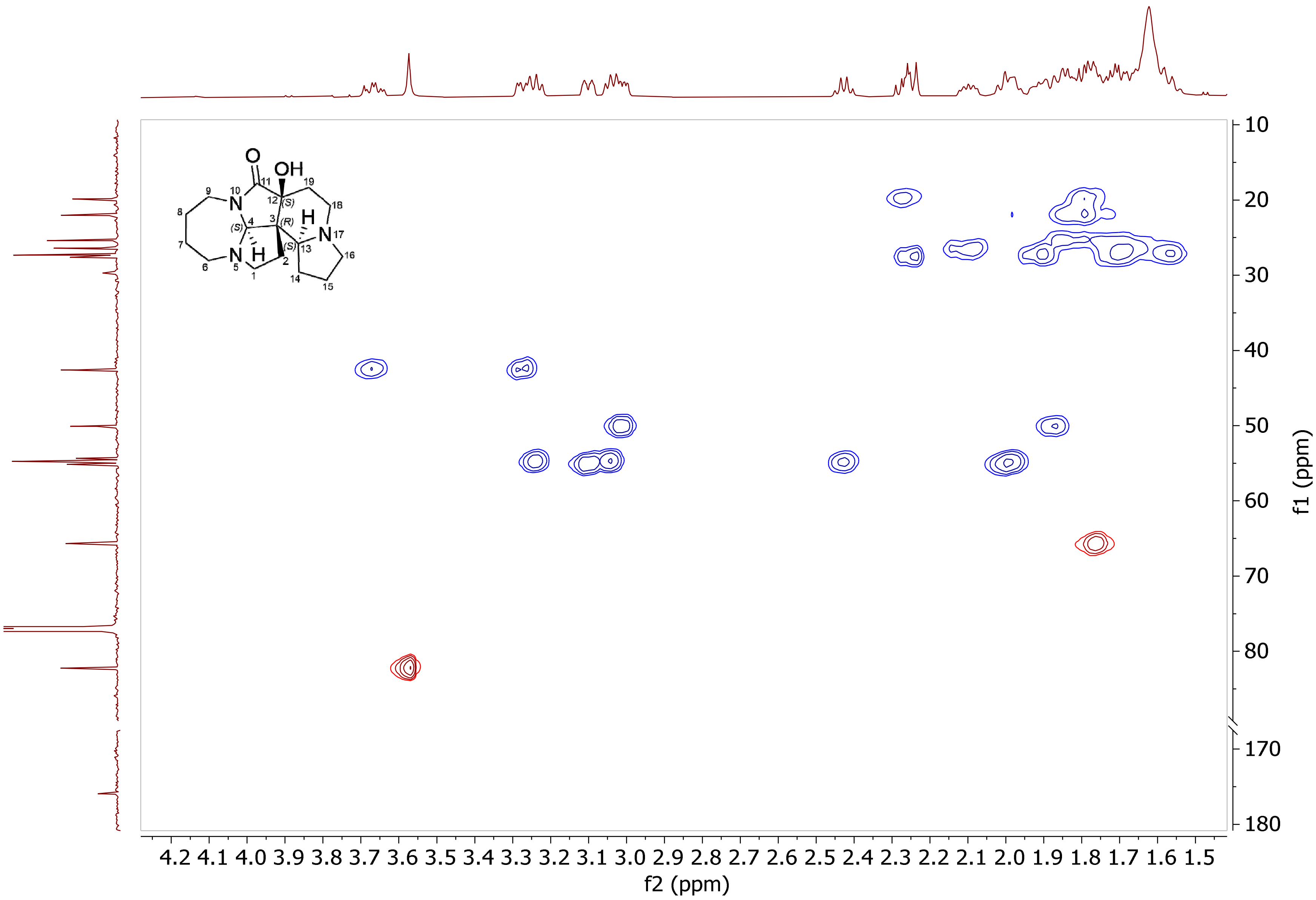

Figure S34. HSQC Spectrum of Pandazepine D (**4**) ($CDCl_3$, 600 MHz)

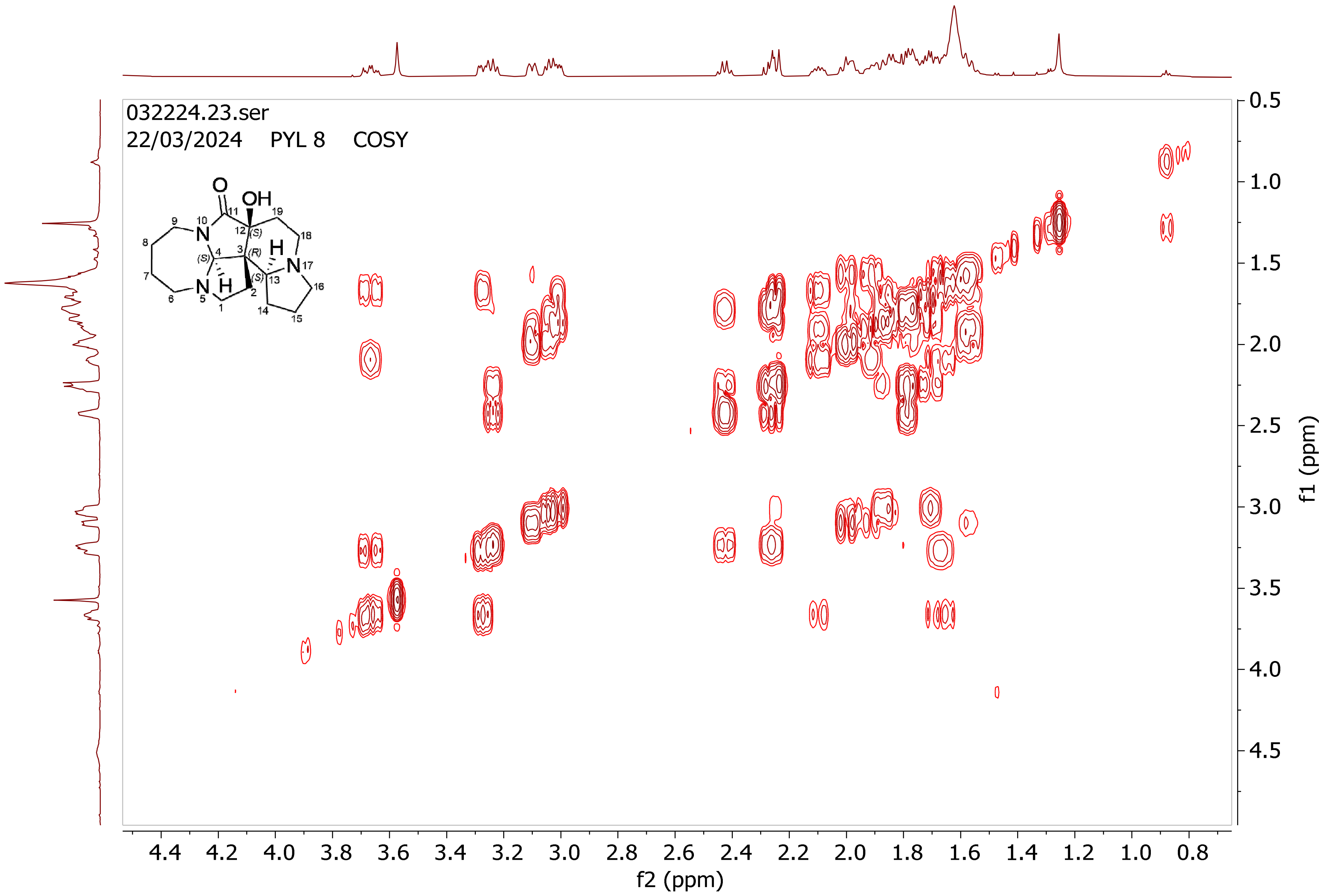

Figure S35. COSY Spectrum of Pandazepine D (**4**) ($CDCl_3$, 600 MHz)

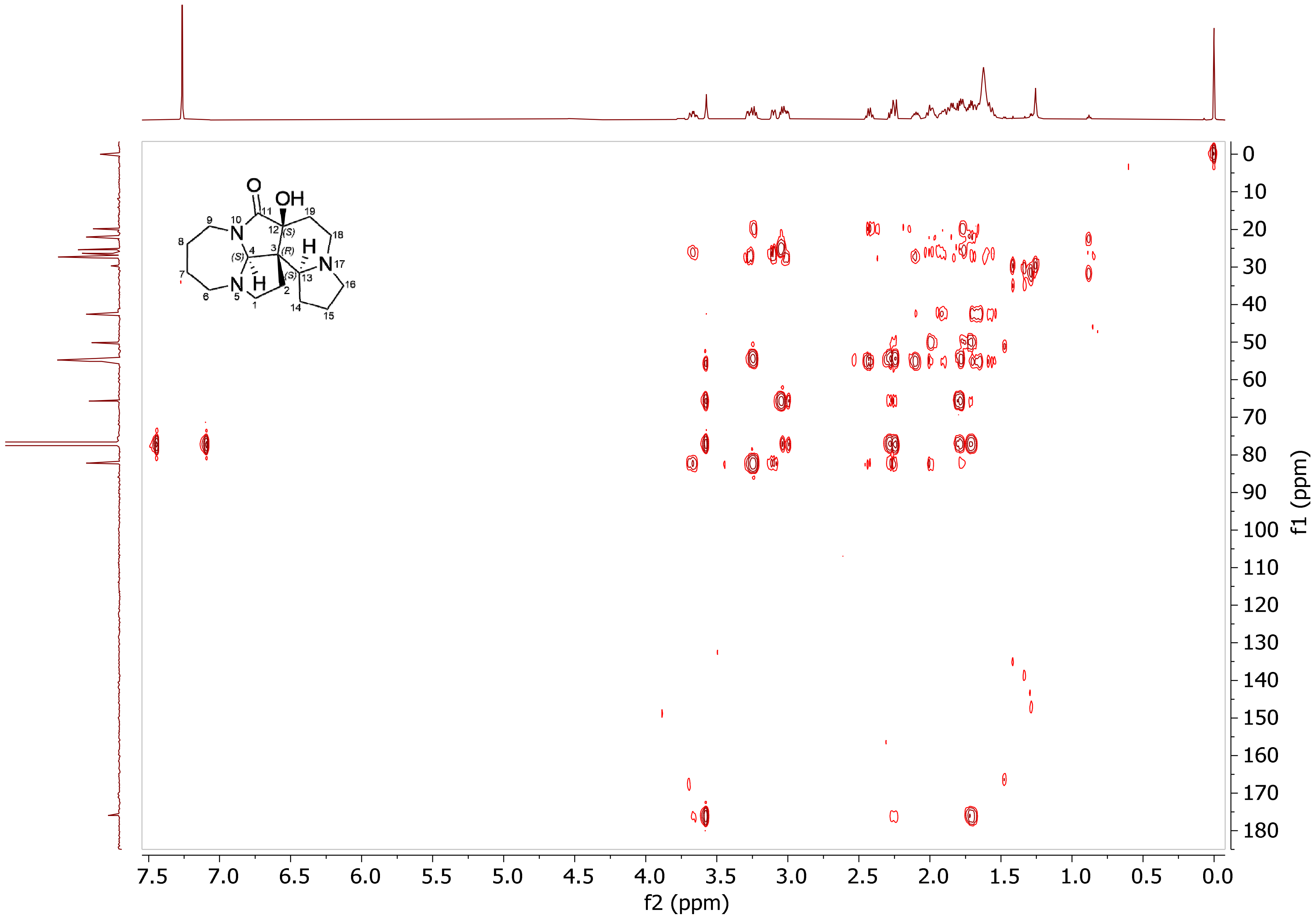

Figure S36. HMBC Spectrum of Pandazepine D (**4**) ($CDCl_3$, 600 MHz)

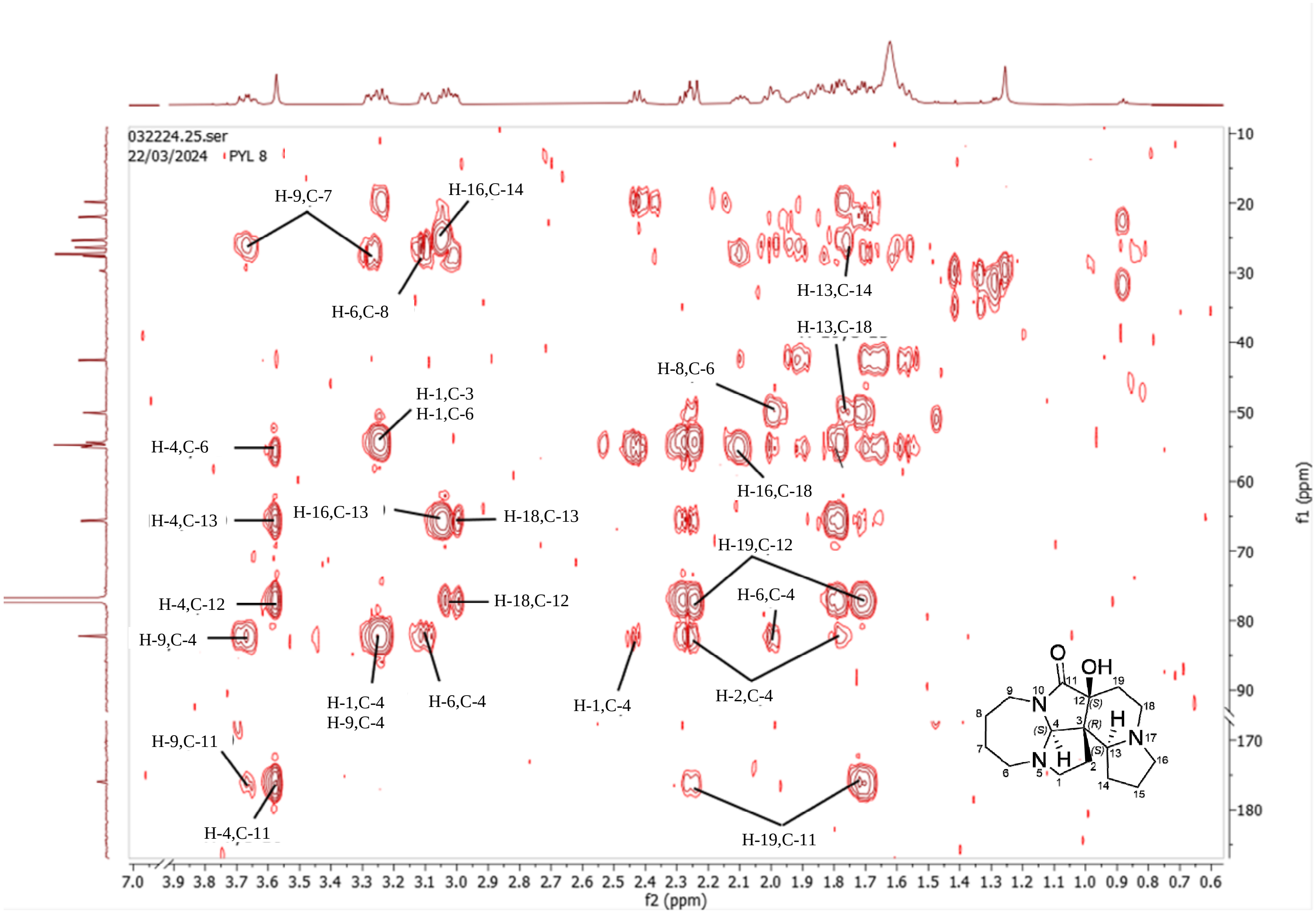

Figure S37. HMBC Spectrum of Pandazepine D (**4**) ($CDCl_3$, 600 MHz)

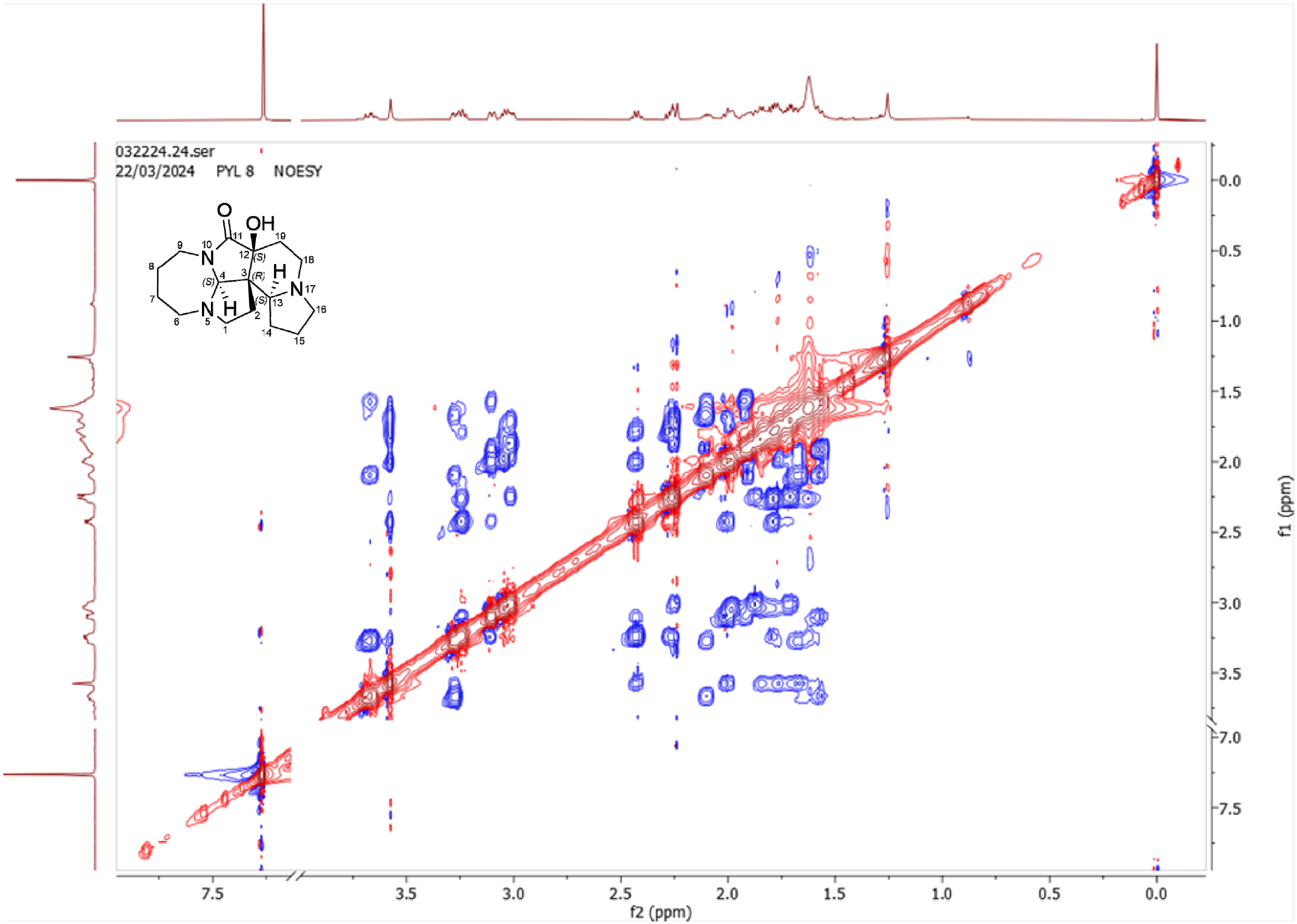

Figure S38. NOESY Spectrum of Pandazepine D (**4**) ($CDCl_3$, 600 MHz)

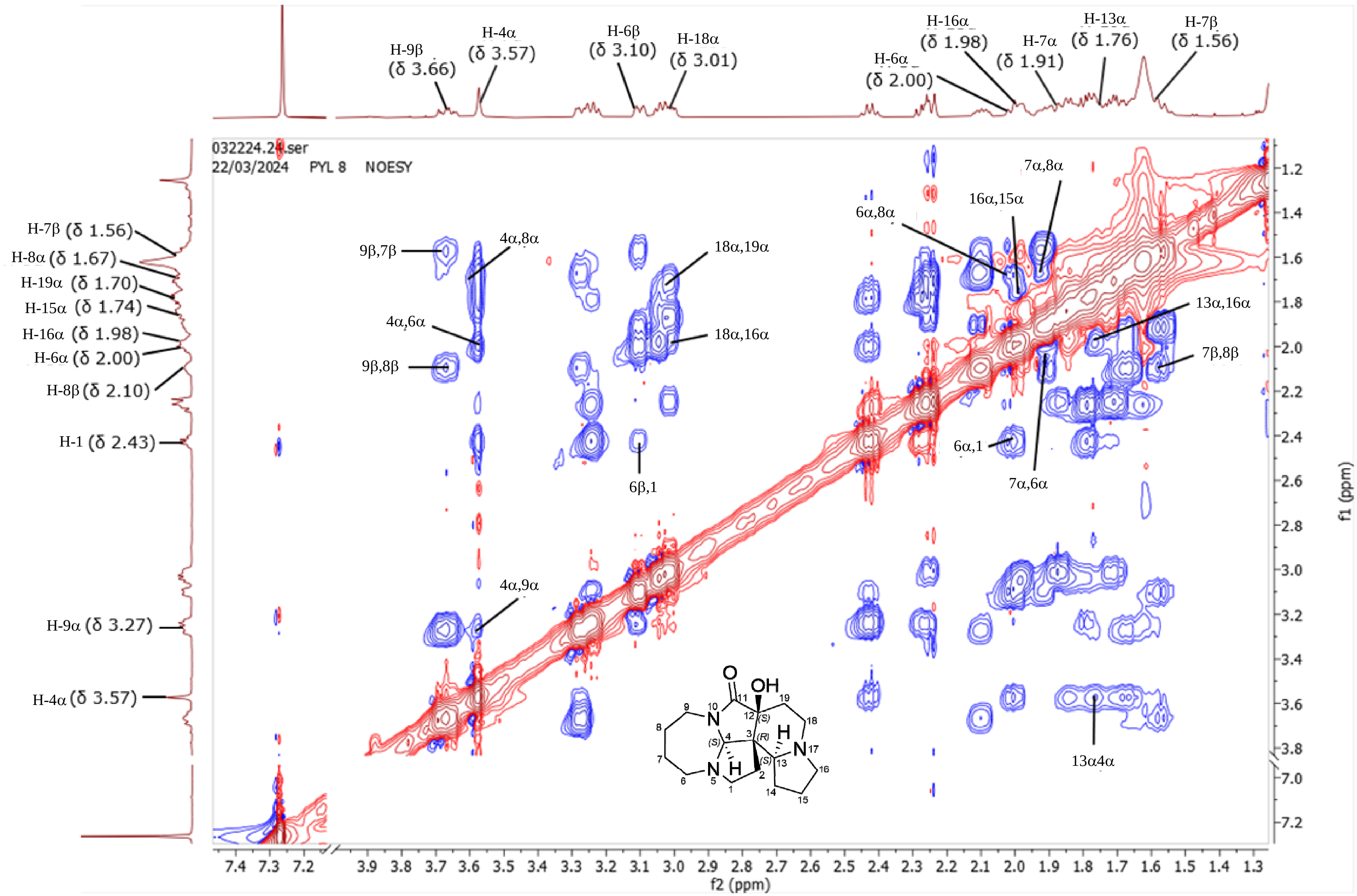

Figure S39. NOESY Spectrum of Pandazepine D (**4**) ($CDCl_3$, 600 MHz)

Sample Name:
Description:
Ionization Mode:ESI+
History:Determine m/z[Peak Detect[Centroid,30,Area];Correct Base[0.5%]];Correct Ba...

Operator:AccuTOF
Mass Calibration data:iCalib
Created:10/23/2024 1:36:26 PM
Created by:AccuTOF

Charge number:1
Tolerance:20.00(ppm), 0.00 .. 30.00(mmu)
Unsaturation Number:0.0 .. 25.0 (Fractio...
Element:^{12}C:10 .. 30, ^{1}H:10 .. 50, ^{14}N:0 .. 5, ^{16}O:0 .. 5

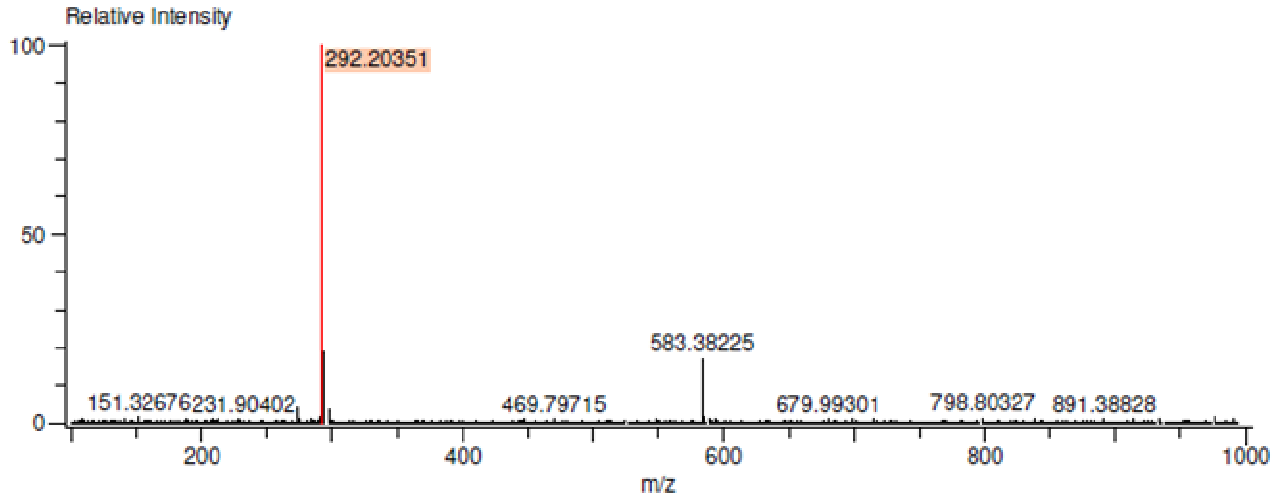

Mass	Intensity	Calc. Mass	Mass Difference (mmu)	Mass Difference (ppm)	Possible Formula
292.20351	64033.29	292.20384	-0.33	-1.13	$^{12}C_{18}{}^{1}H_{28}{}^{16}O_{3}$
		292.20250	1.01	3.46	$^{12}C_{16}{}^{1}H_{26}{}^{14}N_{3}{}^{16}O_{2}$
		292.20652	-3.01	-10.30	$^{12}C_{21}{}^{1}H_{26}{}^{14}N_{1}$
		292.19982	3.69	12.64	$^{12}C_{13}{}^{1}H_{28}{}^{14}N_{2}{}^{16}O_{5}$
		292.19848	5.03	17.23	$^{12}C_{11}{}^{1}H_{26}{}^{14}N_{5}{}^{16}O_{4}$

Figure S40. HRDARTMS of Pandazepine D (**4**)

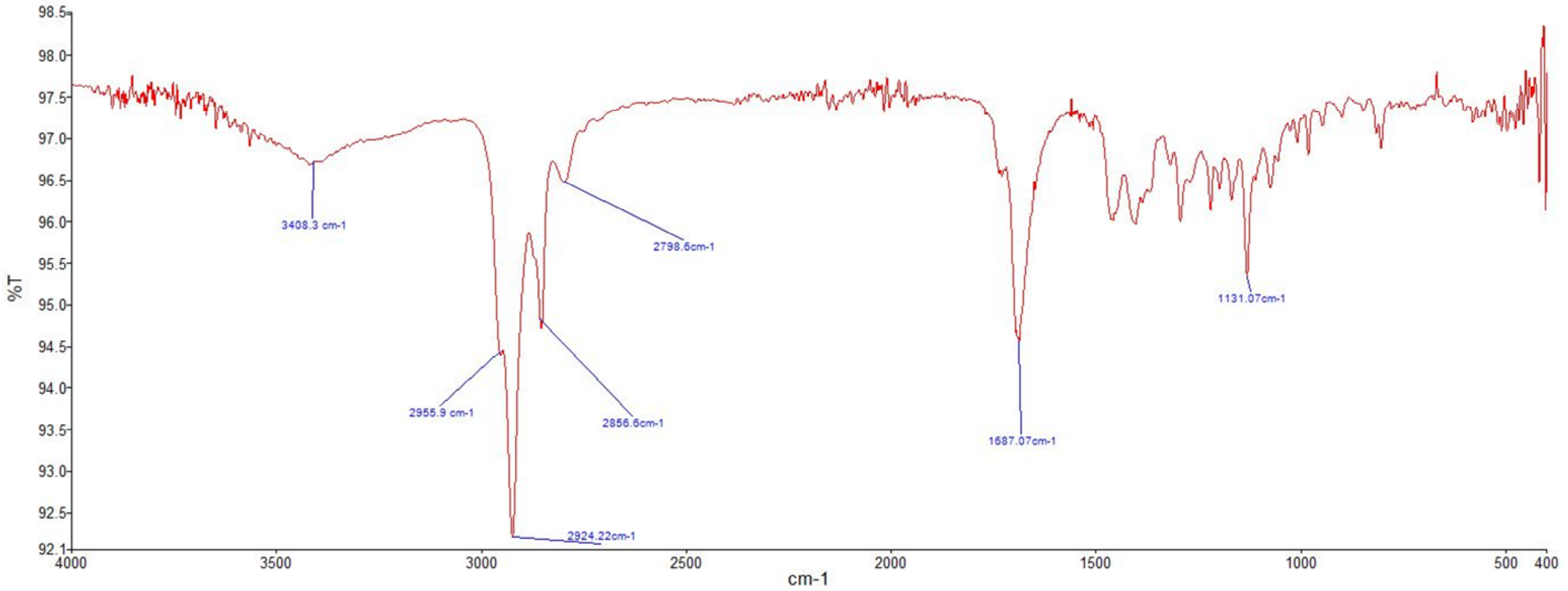

Figure S41. IR Spectrum of Pandazepine D (**4**)

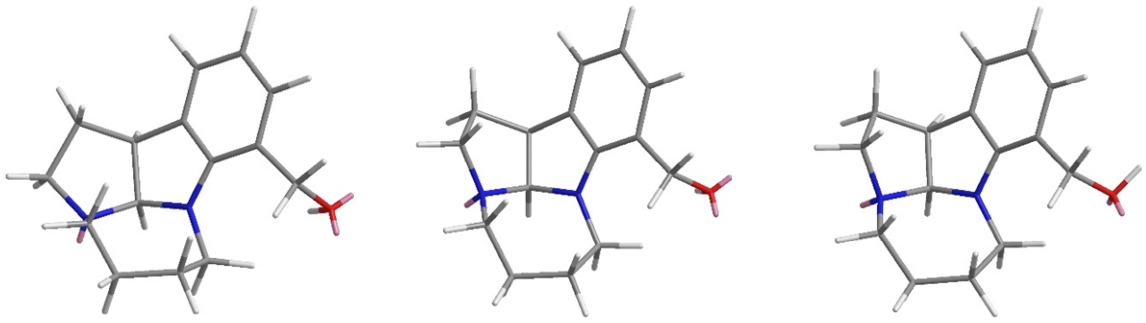

46.4% 46.3% 2.1%

Figure S42. Three Lowest-energy Conformers Calculated at the B3LYP/6-31G(d) Level for (3*R*,4*S*)–**1**. Conformer populations were determined using the Gibbs free energy.

Table S1. DFT B3LYP/6-31G(d) Atomic Cartesian Coordinates (Å) for the Lowest-energy Conformer of (3*R*,4*S*)–**1**.

**Atom**	**X**	**Y**	**Z**
C	-1.05832	1.48926	0.57466
C	-1.16543	0.14681	0.18062
N	0.07132	-0.46703	0.18463
C	1.05854	0.45389	0.79952
C	0.41155	1.84681	0.7859
N	2.39583	0.63192	0.23196
C	2.45054	1.95101	-0.41421
C	1.03975	2.50912	-0.41606
C	-2.14482	2.34891	0.71529
C	-3.39747	1.86002	0.38077
C	-3.5238	0.5588	-0.10777
C	-2.41327	-0.30086	-0.24823
C	0.23447	-1.84647	0.62886
C	2.79342	-0.44244	-0.67737
C	1.34273	-2.51736	-0.18097
C	2.71926	-1.85203	-0.06833
C	-2.6448	-1.63312	-0.90349
O	-3.20074	-2.57329	0.00966
H	-3.22535	-2.15533	0.88718
H	0.59782	2.39907	1.71272
H	1.18182	0.17373	1.85507
H	3.0976	2.59588	0.19233
H	2.87138	1.93809	-1.42626
H	0.51669	2.2259	-1.33735
H	1.01912	3.60034	-0.3418
H	-1.99978	3.36529	1.06373
H	-4.27124	2.49874	0.46879
H	-4.51109	0.21219	-0.41291
H	0.47414	-1.88891	1.69927
H	-0.67255	-2.44174	0.51724
H	2.21861	-0.41084	-1.61257
H	3.84205	-0.26718	-0.95109
H	1.43405	-3.55726	0.15601
H	1.04947	-2.55731	-1.23783
H	3.05197	-1.83939	0.97698
H	3.43411	-2.48716	-0.60744
H	-3.34685	-1.52182	-1.73806
H	-1.73074	-2.04622	-1.33944

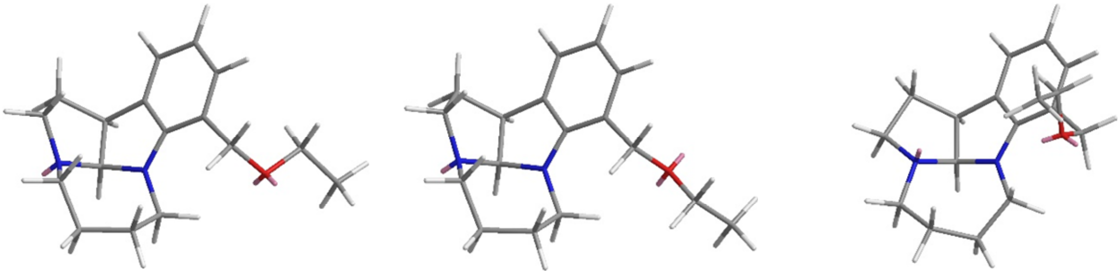

39.0% 33.1% 7.0%

Figure S43. Three Lowest-energy Conformers Calculated at the B3LYP/6-31G(d) Level for (3*R*,4*S*)–**2**. Conformer populations were determined using the Gibbs free energy.

Table S2. DFT B3LYP/6-31G(d) Atomic Cartesian Coordinates (Å) for the Lowest-energy Conformer of (3*R*,4*S*)-**2**.

**Atom**	**X**	**Y**	**Z**
C	1.43220	1.44292	-0.28507
C	0.43090	0.61778	0.25134
N	-0.05946	-0.24380	-0.70570
C	0.73344	-0.10576	-1.94453
C	1.68261	1.09248	-1.75618
N	0.05107	0.16300	-3.20548
C	-0.13196	1.61751	-3.23693
C	1.19756	2.15113	-2.72979
C	2.14055	2.38687	0.45590
C	1.76537	2.58631	1.77308
C	0.70766	1.85044	2.30723
C	0.01805	0.86946	1.56073
C	-0.38886	-1.63050	-0.40142
C	-1.22355	-0.54419	-3.34336
C	-1.50492	-2.12542	-1.32790
C	-1.23698	-1.99754	-2.83571
C	-1.17664	0.20956	2.20088
O	-0.85492	-1.01213	2.86848
C	-0.31962	-0.83351	4.17503
C	-0.15912	-2.20055	4.81167
H	2.73266	0.83324	-1.92822
H	1.36654	-0.99376	-2.07820
H	-0.93673	1.94723	-2.56699
H	-0.35059	1.97955	-4.24655
H	1.90394	2.22863	-3.56521
H	1.09747	3.14022	-2.27394
H	2.94614	2.94989	-0.00186
H	2.27914	3.32681	2.37827
H	0.40271	2.05995	3.33133
H	0.49678	-2.27078	-0.50435
H	-0.73378	-1.77203	0.62072
H	-2.04133	-0.00109	-2.85090
H	-1.46774	-0.55809	-4.41288
H	-1.68954	-3.18279	-1.10066
H	-2.43363	-1.59232	-1.08647
H	-0.31310	-2.52008	-3.11118
H	-2.04901	-2.52168	-3.35629
H	-1.68217	0.89700	2.89176
H	-1.92491	-0.01598	1.43254
H	0.65725	-0.34441	4.11933
H	-0.99815	-0.22228	4.77984
H	-1.12351	-2.71650	4.86533

H	0.50598	-2.82984	4.21098
H	0.25221	-2.11495	5.82132

Table S3. DFT B3LYP/6-31+G(d,p) Atomic Cartesian Coordinates (Å) for the Lowest-energy Conformer of **3a** (3*R*,4*S*,12*R*,13*S*).

**Atom**	**X**	**Y**	**Z**
C	3.62002	-0.97196	-1.27728
C	4.11572	-0.53296	0.11475
C	3.57452	0.82589	0.61516
N	2.12011	0.94701	0.78113
C	1.38624	0.73649	-0.47796
N	1.17167	-0.6686	-0.80402
C	2.20259	-1.56121	-1.33157
C	-0.05866	1.24709	-0.27479
C	-0.61233	0.03832	0.55544
C	0.03864	-1.15538	-0.18095
O	-0.28516	-2.33355	-0.16378
C	0.05115	0.12233	1.95313
C	1.59121	0.09363	1.87104
C	-0.82593	1.44293	-1.59129
C	-2.34335	1.42212	-1.37337
N	-2.71318	0.17646	-0.71934
C	-2.14037	0.05384	0.6252
O	-0.06749	2.48	0.42035
C	-4.14194	-0.05798	-0.54582
C	-4.18572	-1.27144	0.40217
C	-2.86796	-1.17287	1.22435
H	-2.40801	0.95106	1.22428
H	3.7055	-0.13164	-1.98044
H	4.29102	-1.74943	-1.66398
H	5.20995	-0.44434	0.08398
H	3.9005	-1.32391	0.84504
H	2.15128	-2.4917	-0.7535
H	1.96541	-1.82712	-2.37057
H	-0.28915	1.04827	2.42808

H	-0.27709	-0.71407	2.58005
H	1.91535	-0.9511	1.74791
H	2.02567	0.45039	2.81203
H	-0.51332	2.39915	-2.02518
H	-0.56818	0.64325	-2.29325
H	-2.65514	2.30501	-0.78035
H	-2.85581	1.48112	-2.34118
H	0.7818	2.49602	0.90786
H	-4.6451	0.81503	-0.08134
H	-4.6244	-0.24155	-1.51289
H	-4.20049	-2.20011	-0.1759
H	-5.08046	-1.25963	1.03235
H	-3.05794	-1.02955	2.2929
H	-2.26373	-2.07199	1.09906
H	1.91367	1.26496	-1.27698
H	4.03825	1.06183	1.58101
H	3.88851	1.61137	-0.08516

Table S4. DFT B3LYP/6-31+G(d,p) Atomic Cartesian Coordinates (Å) for the Lowest-energy Conformer of **3b** (3*S*,4*S*,12*R*,13*S*).

Atom	X	Y	Z
C	-3.9353	0.82962	-0.73598
C	-4.20572	-0.29749	0.27882
C	-3.3141	-1.55166	0.12882
N	-1.86545	-1.37092	0.26532
C	-1.30416	-0.49076	-0.74022
N	-1.44454	0.96302	-0.41169
C	-2.70688	1.70504	-0.45246
C	0.2358	-0.55579	-0.82002
C	0.57317	0.19764	0.48885
C	-0.38633	1.39365	0.37891
O	-0.30659	2.48728	0.9156
C	0.04653	-0.64649	1.69786
C	-1.45095	-0.98306	1.62241
H	-1.75949	-0.71148	-1.70833
C	0.98174	-1.87824	-0.89999
C	2.48662	-1.57979	-0.75468
N	2.80531	-0.84783	0.49021
C	2.07264	0.4349	0.67537
O	0.63633	0.1568	-2.00203
C	4.20815	-0.40871	0.51846
C	4.27581	0.96911	-0.21119
C	2.8145	1.49533	-0.1824
H	-4.79766	1.50823	-0.75086
H	-3.87124	0.40905	-1.74927
H	-5.24608	-0.63062	0.16411
H	-4.13186	0.10577	1.29737
H	-3.62337	-2.29876	0.87051
H	-3.49549	-1.99776	-0.85849
H	-2.81983	2.2199	0.5099
H	-2.64539	2.49093	-1.21731
H	0.6523	-1.55171	1.76988
H	0.22418	-0.07213	2.61448
H	-2.02555	-0.11406	1.9849
H	-1.68093	-1.81367	2.30021
H	0.79815	-2.34668	-1.87354
H	0.63734	-2.56877	-0.12787
H	3.0407	-2.52523	-0.71815
H	2.81712	-1.04455	-1.65934
H	2.2339	0.71216	1.72699
H	0.14409	0.99476	-2.01115
H	4.51023	-0.29046	1.56736
H	4.85766	-1.16913	0.07381
H	4.62859	0.85436	-1.24135
H	4.97438	1.64631	0.29131
H	2.72392	2.48959	0.26425
H	2.40006	1.54161	-1.19013

Table S5. Experimental and Calculated ^{1}H NMR Chemical Shift (in ppm) of **3**, **3a**, and **3b**.

**Position**	**Exp. 3**	**3a**	**3b**
H-1 (ax)	2.52	2.48369	2.48642
H-1 (eq)	2.49	2.39151	2.42489
H-2 (eq)	1.58	1.35399	1.46640
H-2 (ax)	1.85	1.79327	2.75921
H-4α (pseudoax)	4.03	3.90066	4.27212
H-6α (pseudoax)	2.63	2.69736	2.72522
H-6β (pseudoeq)	2.88	2.80428	2.79734
H-7α (pseudoeq)	1.65	1.49359	1.42313
H-7β (pseudoax)	1.85	1.91398	1.96966
H-8α (pseudoax)	1.67	1.75067	1.76281
H-8β (pseudoeq)	2.01	1.91235	1.87207
H-9α (pseudoax)	3.24	3.17182	3.30361
H-9β (pseudoeq)	3.57	3.65430	3.53793
H-13β (ax)	1.97	1.95508	2.93578
H-14α	2.76	3.14946	2.34228
H-14β	1.64	1.53946	1.83467
H-15α	1.83	1.75423	1.99999
H-15β	1.65	1.45362	1.50109
H-16α	3.09	2.91936	2.80146
H-16β	2.07	2.11115	2.78932
H-18α	2.99	2.75129	3.14414
H-18β	2.25	2.21333	2.53761
H-19α	2.01	1.88845	1.47419
H-19β	1.72	1.58762	2.36284
**MAE**		**0.12**	**0.28**

Table S6. Experimental and Calculated ^{13}C NMR Chemical Shift (in ppm) of **3**, **3a**, and **3b**.

Position	Exp. 3	3a	3b
C-1	41.5	43.68046	43.65351
C-2	24.6	29.59336	36.99355
C-3	70.5	73.78103	78.87975
C-4	76.4	79.11902	83.51832
C-6	54.8	55.69347	54.69979
C-7	24.9	28.57819	28.93605
C-8	26.3	29.80549	29.29253
C-9	42.7	44.92474	46.30421
C-11	175.9	174.2638	173.3008
C-12	48.8	52.41719	54.44676
C-13	63.8	64.83599	66.20692
C-14	22.6	24.21575	26.77780
C-15	21.0	24.73238	25.51469
C-16	54.7	56.43049	55.79569
C-18	49.2	50.49036	46.75795
C-19	32.3	35.87706	30.21243
**MAE**		**2.60**	**4.10**

Functional	Solvent?		Basis Set		Type of Data	
mPW1PW91	PCM		6-31+G(d,p)		Unscaled Shifts	
	3a	3b				
	Isomer 1	Isomer 2	Isomer 3	Isomer 4	Isomer 5	Isomer 6
sDP4+ (H data)	100.00%	0.00%	-	-	-	-
sDP4+ (C data)	100.00%	0.00%	-	-	-	-
sDP4+ (all data)	100.00%	0.00%	-	-	-	-
uDP4+ (H data)	0.02%	99.98%	-	-	-	-
uDP4+ (C data)	100.00%	0.00%	-	-	-	-
uDP4+ (all data)	94.53%	5.47%	-	-	-	-
DP4+ (H data)	100.00%	0.00%	-	-	-	-
DP4+ (C data)	100.00%	0.00%	-	-	-	-
DP4+ (all data)	100.00%	0.00%	-	-	-	-

Figure S44. DP4+ Probability Analysis of ^{1}H and ^{13}C NMR Chemical Shifts of Isomers **3a** and **3b**.

Table S7. DFT B3LYP/6-31+G(d,p) Atomic Cartesian Coordinates (Å) for the Lowest-energy Conformer of **4a** (3*R*,4*S*,12*S*,13*S*).

**Atom**	**X**	**Y**	**Z**
N	1.29642	0.51345	-1.08312
C	0.96789	-0.03476	0.19709
C	-0.48558	-0.49595	0.07209
C	-0.60755	-0.80841	-1.45128
C	0.30745	0.27557	-2.03953
C	-1.44734	0.64991	0.4246
N	-2.81325	0.30201	0.02586
C	-2.92362	0.28064	-1.43357
C	-2.03435	-0.82694	-1.99488
C	2.4232	1.39008	-1.29621
C	3.58826	1.1627	-0.33408
C	3.97548	-0.30961	-0.16004
C	3.08171	-1.08115	0.82654
N	1.70639	-1.27547	0.36725
O	0.22344	0.80167	-3.13692
C	-0.55195	-1.76284	0.94125
C	0.90295	-2.04647	1.32809
C	-1.59059	1.06413	1.89058
C	-3.66446	1.30722	0.65799
C	-3.07852	1.37539	2.06127
O	-0.00041	-2.0684	-1.80843
H	1.11274	0.69141	1.00665
H	-1.13973	1.56045	-0.11442
H	-3.96102	0.0797	-1.72618
H	-2.64518	1.24685	-1.8737
H	-2.02622	-0.76882	-3.09043
H	-2.4904	-1.79814	-1.75698
H	2.75741	1.2640	-2.33273
H	2.06225	2.42083	-1.20333

H	3.35603	1.59873	0.64537
H	4.45402	1.71701	-0.71674
H	4.99984	-0.34427	0.23229
H	3.99862	-0.8171	-1.13227
H	3.53798	-2.0683	0.97365
H	3.10296	-0.57704	1.80186
H	-1.16169	-1.64752	1.84093
H	-0.98107	-2.60905	0.39297
H	1.12898	-3.11614	1.27472
H	1.07393	-1.70678	2.3578
H	-0.98978	1.95884	2.09162
H	-1.28056	0.30312	2.61059
H	-4.71713	1.00903	0.67314
H	-3.57985	2.28448	0.1661
H	-3.5395	0.61441	2.70284
H	-3.24228	2.35196	2.52722
H	0.89406	-2.06044	-1.40552

Table S8. DFT B3LYP/6-31+G(d,p) Atomic Cartesian Coordinates (Å) for the Lowest-energy Conformer of **4b** (3*R*,4*S*,12*R*,13*S*).

Atom	X	Y	Z
N	-1.65613	1.13897	-0.72702
C	-0.99966	-0.11369	-0.40297
C	0.36321	0.27436	0.18074
C	0.5785	1.67682	-0.41791
C	-0.82975	2.23586	-0.46079
C	1.6218	-0.51655	-0.20685
N	2.79435	0.11992	0.44381
C	3.00674	1.52436	0.0706
C	1.734	2.38625	0.25485
C	-3.09241	1.25752	-0.90163
C	-3.77396	-0.02043	-1.40425
C	-3.99027	-1.07287	-0.30671
C	-2.72163	-1.69004	0.29868
N	-1.79069	-0.65559	0.69129
O	-1.14111	3.41403	-0.41439
C	0.08377	0.20944	1.70401
C	-0.93574	-0.91693	1.82679
C	1.81599	-1.94979	0.26196
C	3.93141	-0.75293	0.15186
C	3.32379	-2.15701	0.10994
O	0.89586	1.60078	-1.84241
H	-0.91948	-0.74664	-1.29385
H	1.74989	-0.519	-1.29903
H	3.80644	1.94872	0.68882
H	3.33964	1.5956	-0.97305
H	1.904	3.37101	-0.19615
H	1.53348	2.55174	1.3193
H	-3.52564	1.58726	0.05037
H	-3.25923	2.07062	-1.61801
H	-4.7624	0.25553	-1.7922
H	-3.21599	-0.45589	-2.24151
H	-4.58323	-0.61915	0.49863
H	-4.60351	-1.88463	-0.71715
H	-3.03285	-2.27528	1.17337
H	-2.26386	-2.40119	-0.39983
H	0.96644	0.01715	2.32081
H	-0.35908	1.14783	2.06356
H	-0.47654	-1.90635	1.76605
H	-1.48249	-0.85042	2.77263
H	1.23922	-2.66875	-0.32726
H	1.56666	-2.08922	1.31765
H	4.70148	-0.67264	0.92541
H	4.38921	-0.52509	-0.81848
H	3.71248	-2.79611	0.90952
H	3.54816	-2.63726	-0.84929
H	0.80915	2.50963	-2.18989

Table S9. Experimental and Calculated ^{1}H NMR Chemical Shift (in ppm) of **4**, **4a**, and **4b**.

**Position**	**Exp. 4**	**4a**	**4b**
H-1 (ax)	2.42	2.43533	2.96358
H-1 (eq)	3.24	3.10162	2.75653
H-2 (ax)	1.79	1.64339	1.23160
H-2 (eq)	2.26	2.38766	2.35929
H-4α (pseudoax)	3.57	3.51918	4.88005
H-6α (pseudoax)	2.00	2.06219	3.02418
H-6β (pseudoeq)	3.10	2.99632	3.00837
H-7α (pseudoeq)	1.91	1.74202	1.58411
H-7β (pseudoax)	1.56	1.64769	1.95029
H-8α (pseudoax)	1.67	1.69269	1.71839
H-8β (pseudoeq)	2.10	1.96518	1.81573
H-9α (pseudoax)	3.27	3.11781	3.45959
H-9β (pseudoeq)	3.66	3.65368	3.16360
H-13α (ax)	1.76	1.61193	2.95012
H-14α	1.84	1.80639	1.53428
H-14β	1.72	1.64803	1.47519
H-15α	1.80	1.62885	1.57451
H-15β	1.74	1.78371	1.75659
H-16α	1.98	1.97962	2.16465
H-16β	3.04	2.84269	2.86492
H-18α	1.87	1.77272	2.54701
H-18β	3.01	2.79929	2.75566
H-19α	2.24	1.98763	1.51979
H-19β	1.70	1.51923	1.79576
**MAE**		**0.11**	**0.42**

Table S10. Experimental and Calculated ^{13}C NMR Chemical Shift (in ppm) **4**, **4a**, and **4b**.

Position	Exp. 4	4a	4b
C-1	54.7	56.57194	47.81842
C-2	19.8	23.57845	31.36023
C-3	54.4	60.10603	58.63423
C-4	82.2	81.26554	82.0908
C-6	55.1	56.05827	50.81845
C-7	27.3	30.63619	33.88263
C-8	26.4	30.34642	29.01951
C-9	42.6	44.33416	47.78547
C-11	175.9	173.4135	171.3913
C-12	77.1	77.87141	79.92398
C-13	65.7	66.78291	62.34106
C-14	25.4	28.46095	28.6924
C-15	22.0	26.67265	25.32004
C-16	54.9	56.25943	54.88507
C-18	50.1	50.97930	48.01696
C-19	27.6	29.94683	31.1155
**MAE**		**2.40**	**4.00**

Functional	Solvent?		Basis Set		Type of Data	
mPW1PW91	PCM		6-31+G(d,p)		Unscaled Shifts	
	4a	4b				
	Isomer 1	Isomer 2	Isomer 3	Isomer 4	Isomer 5	Isomer 6
sDP4+ (H data)	100.00%	0.00%	-	-	-	-
sDP4+ (C data)	100.00%	0.00%	-	-	-	-
sDP4+ (all data)	100.00%	0.00%	-	-	-	-
uDP4+ (H data)	100.00%	0.00%	-	-	-	-
uDP4+ (C data)	100.00%	0.00%	-	-	-	-
uDP4+ (all data)	100.00%	0.00%	-	-	-	-
DP4+ (H data)	100.00%	0.00%	-	-	-	-
DP4+ (C data)	100.00%	0.00%	-	-	-	-
DP4+ (all data)	100.00%	0.00%	-	-	-	-

Figure S45. DP4+ Probability Analysis of ^{1}H and ^{13}C NMR Chemical Shifts of Isomers **4a** and **4b**.
